# Comprehensive Glycomic and Proteomic Analysis of Mouse Striatum and Lateral Hypothalamus Following Repeated Exposures to Cocaine or Methamphetamine

**DOI:** 10.1016/j.mcpro.2024.100803

**Published:** 2024-06-15

**Authors:** Manveen K. Sethi, Riccardo Maccioni, John D. Hogan, Tomoya Kawamura, Vez Repunte-Canonigo, Jihuan Chen, Joseph Zaia, Pietro Paolo Sanna

**Affiliations:** 1 Center for Biomedical Mass Spectrometry, Department of Biochemistry and Cell Biology, Boston University School of Medicine, Boston, Massachusetts, USA; 2 Department of Immunology and Microbiology, The Scripps Research Institute, La Jolla, California, USA; 3 Bioinformatics Program, Boston University, Boston, Massachusetts, USA

**Keywords:** addiction, neurobiology, drug abuse, proteomics, glycomics, mass spectrometry, glycosaminoglycans, proteoglycans, cocaine, methamphetamine, lateral hypothalamus, striatum, heparan sulfate, chondroitin sulfate

## Abstract

Substance use disorder is a major concern, with few therapeutic options. Heparan sulfate (HS) and chondroitin sulfate (CS) interact with a plethora of growth factors and their receptors and have profound effects on cellular signaling. Thus, targeting these dynamic interactions might represent a potential novel therapeutic modality. In the present study, we performed mass spectrometry–based glycomic and proteomic analysis to understand the effects of cocaine and methamphetamine (METH) on HS, CS, and the proteome of two brain regions critically involved in drug addiction: the lateral hypothalamus and the striatum. We observed that cocaine and METH significantly alter HS and CS abundances as well as sulfate contents and composition. In particular, repeated METH or cocaine treatments reduced CS 4-*O*-sulfation and increased CS 6-*O*-sulfation. Since C4S and C6S exercise differential effects on axon growth, regeneration, and plasticity, these changes likely contribute to drug-induced neural plasticity in these brain regions. Notably, we observed that restoring these alterations by increasing CS *4-0* levels in the lateral hypothalamus by adeno-associated virus delivery of an shRNA to arylsulfatase B (N-acetylgalactosamine-4-sulfatase) ameliorated anxiety and prevented the expression of preference for cocaine in a novelty induced conditioned place preference test during cocaine withdrawal. Finally, proteomics analyses revealed a number of aberrant proteins in METH- and cocaine-treated *versus* saline-treated mice, including myelin proteolipid protein, calcium/calmodulin-dependent protein kinase type II subunit alpha, synapsin-2, tenascin-R, calnexin, annexin A7, hepatoma-derived growth factor, neurocan, and CSPG5, and oxidative phosphorylation among the top perturbed pathway. Taken together, these data support the role of HS, CS, and associated proteins in stimulants abuse and suggest that manipulation of HSPGs can represent a novel therapeutic strategy.

## Highlights

• Cocaine and METH significantly alter HS and CS abundances and sulfate contents.
• METH/cocaine treatment reduced CS 4-O- and increased CS 6-O-sulfation *versus* control.
• Increasing CS 4-0 by silencing ARSB improved anxiety and prevented cocaine preference.
• Proteomics data showed a tight clustering of the drug-treated *versus* control samples.

## Graphical Abstract

**Figure:**
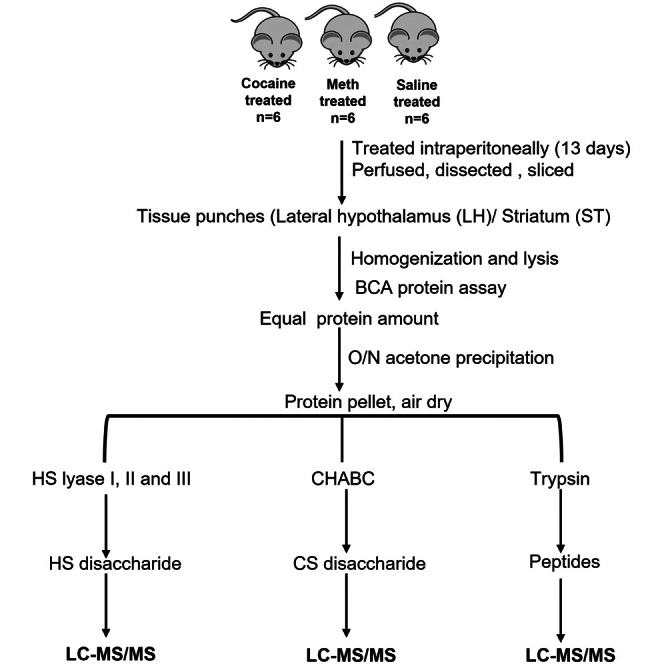

## In Brief

Drug abuse is a major concern with few therapeutic options. Heparan sulfate and chondroitin sulfate interact with growth factors and their receptors and have profound effects on cellular signaling. Thus, targeting these interactions represents a novel therapeutic modality. We performed glycomic and proteomic analysis of cocaine and methamphetamine-treated mouse brains *versus* control (saline-treated) from lateral hypothalamus and striatum and identified key alterations in chondroitin sulfate and heparan sulfate sulfation patterns and associated proteins and pathways, opening avenues for novel therapeutic strategies for stimulant abuse.

Drug abuse is a major concern, with few therapeutic options (1). Drug-related deaths have grown drastically between 1999 and 2015, and overdose deaths of any kind of drug for Americans 20 to 64 years old increased by 5.5 percent per year (2). About 20.4 million people were diagnosed with substance use disorder, and nearly 71,000 people died of overdose in 2019, with cocaine and methamphetamine (METH) deaths reaching about 15,000 in 2019 (3). This alarming trend drives the need to unravel the neurobiological mechanisms that contribute to drug abuse to develop new therapeutic targets and treatment strategies.

Heparan sulfate (HS) and chondroitin sulfate (CS) are unbranched polysaccharides composed of alternating uronic acid (either glucuronate or iduronate) and GalNAc or GlcNAc disaccharide units, respectively, attached to a serine or threonine residue of the core protein through a characteristic tetrasaccharide linker. HS and CS glycosaminoglycans (GAGs) with their disaccharide units, linkages, modifications, functions; and structural information, including Lawrence code and traditional nomenclature, chemical structures and *m/z* values are shown in Table 1 and Supplemental Table S1, (4, 5, 6, 7, 8). The interactions between GAGs polysaccharides, growth factors, and growth factor receptors regulate cellular signaling in a spatially and temporally defined manner (9). Thus, the dynamic regulation of HS, CS, and its interaction partners might have potential as a novel therapeutic modality (10).

**Table 1:** Type of glycosaminoglycans (GAGs), chondroitin sulfate (CS) and heparan sulfate (HS) unsaturated disaccharides disaccharide unit, linkage, functions, disaccharide Lawrence and traditional nomenclature, *m/z*, and other description used in the manuscript (4, 5, 6, 7, 8)

Type of GAG	Disaccharide unit with linkage	Modifications	Functions	Disaccharides			
Chondroitin sulfate (CS)	β-1,3 GalNAc and β-1,4 HexA (Glc or IdoA)	Sulfation at the C2 position of GlcA, C4, and/or C6 position of GalNAc	Cell migration, proliferation and signaling, and extracellular matrix–receptor interaction	Lawrence codes	Traditional names	*m/z* (z = 1)	Other description
				D0a0	ΔHexA-GalNAc	378	unsulfated
				D0a4	ΔHexA-GalNAc4S	458	4-*O* sulfated (4S)
				D0a6	ΔHexA-GalNAc6S	458	6-*O* sulfated (6S)
				D2a4	ΔHexA2S-GalNAc4S	538	2-*O 4-O* sulfated
				D2a6	ΔHexA2S-GalNAc6S	538	2-*O 6-O* sulfated
				D0a10	ΔHexA-GalNAc4S6S	538	4-*O* 6*-O* sulfated
				D2a10	ΔHexA2S-GalNAc4S6S	617	2-*O* 4-*O* 6*-O* sulfated
Heparin sulfate (HS)	β-1,4 GlcNAc and α-1,4 Hex A (Glc or IdoA)	Epimerization of GlcA to IdoA. Sulfation at 2*O*-position of IdoA, the N-, 6*O-* and 3*O*-position of GlcNS/GlcNAc	Developmental processes, angiogenesis, and blood coagulation	D0A0	ΔHexA-GlcNAc (IVA)	378	Unsulfated *N-*acetylated
				D0A6	ΔHexA-GlcNAc(6S) (IIA)	458	6*-O* sulfated *N-*acetylated
				D2A0	ΔHexA(2S)-GlcNAc (III-A)	458	2-*O* sulfated *N-*acetylated
				D2A6	ΔHexA(2S)-GlcNAc(6S) (IA)	538	2-*O, 6-O* sulfated *N-*acetylated
				D0S0	ΔHexA-GlcNS (IVS)	416	*N*-sulfated (n*o O-sulfation)*
				D0S6	ΔHexA-GlcNS(6S) (IIS)	496 (247.5; z = 2)	6*-O* sulfated *N-*sulfated
				D2S0	ΔHexA(2S)-GlcNS (IIIS)	496 (247.5; z = 2)	2-*O* sulfated *N-*sulfated
				D2S6	ΔHexA(2S)-GlcNS(6S) (IS)	576 (287.5; z = 2)	2-*O, 6-O* sulfated *N-s*ulfated

GlcA, glucuronic acid; Hex, hexuronic acid; IdoA, iduronic acid.

With the advent of high-throughput genomic and proteomic techniques, a number of studies have generated protein and gene expression profiles for neuroanatomical substrates in rodents, nonhuman primate models of substance use disorder, and human postmortem brain tissue from victims of drug abuse (11). In the present work, we sought to define the effects of drugs of abuse on HS and CS levels and structures.

We previously showed that stimulants like cocaine and METH greatly increase HS content and sulfation levels in the lateral hypothalamus (LH) and that HS contributes to the regulation of cocaine seeking and taking. Accordingly, our previous gene expression studies showcased that the rats with a history of excessive cocaine self-administration had increased expression of the HS proteoglycan syndecan-3 in the LH (12), a brain region involved in motivation for both natural rewards and drug abuse (13, 14). Moreover, syndecan-3 null mice self-administered higher quantities of cocaine than WT mice, and the glial cell line–derived neurotrophic factor, which binds syndecan-3 through its HS chains, significantly increased cocaine intake in syndecan-3 null mice relative to WT mice (12). We showed that the ability of HS-binding neuropeptide glial cell line–derived neurotrophic factor to increase cocaine intake was potentiated by the elimination of HS binding. Additionally, the delivery of heparanase, the endo-β-D-glucuronidase that cleaves HS chains, promoted cocaine self-administration in a manner distinct from the action of syndecan-3, indicating that HS is a resilience factor for cocaine abuse and a novel therapeutic target for cocaine addiction (15).

A fundamental question raised by these results is the mechanism of action of GAGs in drug abuse and its motivation. To address this question, we applied proteomic and glycomic analysis to measure changes in response to cocaine and METH, two widely used stimulants, on HS, CS, and protein levels, respectively, from each of the two brain regions associated with motivation for both natural rewards and drugs of abuse, the LH and the ST (13, 14, 16, 17, 18, 19, 20, 21, 22). We report significant changes in HS and CS disaccharide abundances and percent sulfate levels observed in the drug-treated mice compared to saline-treated mice for both LH and ST. In addition, we observed that both stimulants induced a reduction of CS 4-*O*-sulfation and an increase in 6-*O*-sulfation. Notably, adeno-associated virus (AAV) delivery of an shRNA to arylsulfatase B (N-acetylgalactosamine-4-sulfatase, ARSB), to increase CS *4-0* levels in the LH, reduced anxiety and prevented the expression of preference for cocaine in a novelty-induced conditioned place preference test during cocaine withdrawal. Finally, the proteomics data showed a tight and separate clustering of the cocaine and METH-treated samples from the saline-treated samples, indicating the similarity between the drug-treated groups in both brain regions. These data show a number of perturbed proteins and aberrant pathways in stimulant-treated mice. Our results also indicated qualitative changes in glycomic and proteomic maps between the two brain regions. Taken together, our study defines novel relationships among HS, CS, and associated proteins with drug abuse in a mouse model, providing a new focus for the investigation of therapeutics.

## Experimental Procedures

### Materials

Heparin lyase enzymes I, II, and III and chondroitinase ABC enzyme (CHABC) were purchased from New England Biolabs. Trypsin Gold, mass spectrometry (MS) grade, was purchased from Promega. Trizma base, sodium chloride, Triton X-100, calcium chloride, ammonium bicarbonate, 2,2,2-trifluoroethanol were all purchased from Sigma-Aldrich. EDTA was purchased from Fluka. Ammonium acetate and C-18 zip tips (100 μl), LC-MS grade acetonitrile (ACN), and water were all purchased from Thermo Fisher Scientific. Complete mini protease inhibitor cocktail tablet (EDTA-free) was purchased from Roche Diagnostics.

### Experimental Design and Statistical Rationale

Fresh frozen mouse tissue punches from LH and striatum (ST) were obtained from six cocaine-treated (C1, C2, C3, C4, C5, and C6), six METH-treated (M1, M2, M3, M4, M5, and M6), and six saline-treated (S1, S2, S3, S4, S5, and S6) mice, for a total of n = 18 samples per brain region. All the samples for each brain region were handled for sample processing and acquired on the instrument (for both glycomics and proteomics) at the same time to avoid any sample handling or instrument bias. The two brain regions were processed and acquired about a year apart. For all samples (glycomics and proteomics), two technical LC-MS/MS runs were acquired except for proteomics data for ST brain region. Standard disaccharides, peptide retention time mixtures, and blanks were run routinely in between the samples. In addition, the disaccharide and peptide samples were spiked with internal controls. The total ion current (TIC) levels for all proteomics data files are shown in the Supplemental PDF S1, all TICs were within 2-fold intensity range of 1.8 to 3.04 E9 (for LH; Supplemental Fig. S1*A*) and 2.0 to 4.00 E9 (For ST; Supplemental Fig. S1B). The spiked internal standard peptide (*m/z* 496.28; Supplemental Fig. S2) for proteomics and synthetic disaccharide for glycomics (*m/z* 552; Supplemental Fig. S3) was shown to be consistent concerning signal intensity and retention time for cocaine, METH, and saline samples for LH and ST. Each group cocaine, METH, and saline had n = 6 biological replicate and all the data were tested for multiple corrections using a Benjamin–Hochberg corrected *p* value cut-off of 0.2.

### Mice

Adult male C57BL/6J mice were housed in a climate-controlled vivarium on a 12 h/12 h reverse light/dark cycle (lights on 9:00 AM) with food and water available *ad libitum* throughout the study. The experimental protocols were performed in accordance with US National Institutes of Health guidelines on animal care and were approved by The Scripps Research Institute Animal Care and Use Committee (15). All reporting in the manuscript follows the recommendations in the ARRIVE guidelines for reporting research work involving live animals (23).

### Treatment and Tissue Collection

One cohort of adult male C57BL/6J mice was treated with either cocaine (20 mg/kg/10 ml once per day) or METH (2.5 mg/kg/10 ml twice per day) intraperitoneally for 13 days, as previously reported in (17). After 48 h from the last injection, mice were euthanized by decapitation under heavy isoflurane (5%) anesthesia, brains were immediately harvested, dissected, and brain regions were snap-frozen on dry ice. Another cohort of adult male C57BL/6J mice, previously microinjected with AAV (see below) in the LH, was treated intraperitoneally with cocaine (10 mg/kg/10 ml) for 14 days. After the behavioral experiments were performed, animals were euthanized as described above.

### Microinfusion of AAV

AAV vectors (AAV1 serotype) expressing either a bicistronic transcript including the ORFs of mARSB and GFP linked through an IRES (AAV1-mARSB) or expressing GFP alone (AAV1-GFP) from a CMV promoter and antisense to ARSB under the control of U6 promoter with titers in the range of 8.4 × 10^12^ to 1.0 × 10^13^ genomic particles per milliliter were prepared according to conventional protocols. Animals were anesthetized for intra-LH virus injections. Briefly, mice were immobilized in a stereotaxic frame in the flat-skull position (Kopf Instruments), and a bilateral stainless-steel injector (33 ga, extended 1 cm below the tip of a 26-ga sleeve tubing) was lowered into the LH (coordinates: −0.94 mm anterior to bregma, ±1.2 mm lateral, and −5.4 mm ventral to skull surface at bregma). The AAV solution was delivered slowly through the injector by a syringe pump (0.5 μl each at a flow rate of 0.05 μl per min, Kd Scientific). The injector remained in place for at least 10 min and then was withdrawn slowly to avoid backflow of the virus. The effects of the AAV microinjections on ARSB expression levels were confirmed by MS (Supplemental Fig. S4) based on CS disaccharide analysis, as indicated below.

### Si-RNA and Overexpressing ARSB Experiments Anxiety and Novelty Experiments

#### Elevated Plus Maze

After 10 days of withdrawal, AAV-microinjected mice were tested in the elevated plus maze (EPM). The EPM consisted of a central platform (5 × 5 cm, W × L) and two open arms (5 × 30 cm, W × L) aligned perpendicularly to two closed transparent arms (5 × 30 cm, W × L) at the height of 40 cm from the ground. Mice were tested individually by being placed in the center of the maze facing an open arm. The spontaneous activity of mice was automatically recorded for 5 min, during which the experimenter left the room. After each experiment, the apparatus was cleaned with 70% ethanol allowing time for evaporation before testing the following mouse. Data were calculated as time spent in the open arms (seconds) and, in agreement with Maccioni *et al*., 2021 (24), as the percentage of open arm entries (number of entries into open arms divided by the number of entries into open arms + number of entries into closed arms). The analysis was automatically performed by ANY-Maze software. Mice that fell from the maze were discarded from the analysis.

#### Novelty-Induced Place Conditioning

Competition between novelty and cocaine was tested using a place conditioning procedure. Briefly, mice were treated with cocaine (10 mg/kg) and immediately confined in the cocaine-associated compartment for 15 min daily for 14 days to induce place conditioning. After 14 days from the last cocaine administration, the cocaine-associated compartment was connected to an easily discriminable novel compartment which differed by visual and tactile clues (24, 25, 26) which was used as the novel stimulus (27). Mice were left free to explore the two connected compartments for 5 min. Preference was expressed in terms of time (s) spent in the cocaine-associated or in the novel compartment.

### Tissue Lysis of Mouse LH and ST

Fresh frozen mouse tissue punches: (LH and ST processed at 1 year apart) cocaine-treated; C1, C2, C3, C4, C5, and C6, METH-treated; M1, M2, M3, M4, M5, and M6, and saline-treated; S1, S2, S3, S4, S5, and S6) were lysed described (15) with buffer containing 50 mM Tris–HCl (pH = 7.4), 150 mM NaCl, 2 mM EDTA, protease inhibitor cocktail tablet, and 0.5% Triton X-100. Tissues were homogenized and incubated for 30 min on ice. This was followed by centrifugation at 12,000 RPM, 4 °C for 30 min. The supernatant was collected into separate tubes, and the protein concentration of each sample was determined using the Pierce bicinchoninic acid assay (Thermo Fisher Scientific). The samples were stored at −20 °C until further use. The methodology is shown in Supplemental Figure S5.

### Enzyme Digestion for HS and Size-Exclusion Chromatography

The HS release and size-exclusion chromatography (SEC) were carried out as previously described (15). Briefly, an equal amount of protein for each sample was taken, and a 5-fold volume of acetone was added and incubated at −20 °C overnight. The samples were centrifuged at 20,000×*g*, 4 °C for 30 min. The pellets were washed with equal volumes of acetone: water (6:1) and centrifuged again. The supernatant was discarded, and the pellet was allowed to dry in the air. The protein pellet from each sample was resuspended in a volume of 100 μl 20 mM Tris–HCl buffer pH 7.4, in the presence of 5 mM CaCl_2_, 5 mM ammonium acetate, 10 mU each of heparin lyase I, heparin lyase II, and heparin lyase III (NEB) (8) were added, and the mixture sonicated briefly. The mixture was incubated at 37 °C overnight using Eppendorf thermomixer at a speed of 400 RPM. The digested solution was added directly onto a 3 kDa cut-off filtration unit (Amicon, 0.5 ml) and centrifuged at 14,000*g* for 20 min. The filter was washed with 100 μl water, centrifuged again, and the HS disaccharides were collected as a flow-through. The flow-through was dried by vacuum centrifugation and desalted using an SEC column (Superdex peptide PC 3.2/30, GE Healthcare), using 25 mM ammonium acetate in 5% ACN (pH = 4.4) as the mobile phase at an isocratic flow 0.04 ml/min for 60 min. The HS disaccharides eluted between 35 to 45 min and were detected using UV absorbance at 232 nm. The cleaned HS disaccharide samples were dried by vacuum centrifugation and stored at −20 °C until analyzed using LC-MS/MS.

### Enzyme Digestion for CS and SEC

As indicated above, an equal amount of protein for each sample was taken, a 5-fold volume of acetone was added, and incubated at −20 °C overnight. The samples were centrifuged at 20,000×*g*, 4 °C for 30 min to obtain a protein pellet which was air dried. The protein pellet from each sample was resuspended in a volume of 100 μl 20 mM Tris–HCl buffer pH7.4 in the presence of 5 mM ammonium acetate and 20 mU of CHABC (NEB) (20) and sonicated briefly. The workup for CS disaccharides was the same as for HS disaccharides (above).

### Enzyme Digestion for Peptides and C-18 Zip-Tip Cleanup

As indicated above, an equal amount of protein for each sample was taken, and five times acetone was added and further incubated at −20 °C overnight and centrifuged at 20,000*g*, 4 °C, for 30 min, to obtain a protein pellet which was air dried. The protein pellet from each sample was resuspended in 100 μl of 50% 2,2,2-trifluoroethanol/50% 50 mM ammonium bicarbonate and sonicated briefly to dissolve the pellet and incubated at 60 °C for 2 h on an Eppendorf thermomixer at a speed of 400 RPM. After 2 h, the solution was cooled, and 100 μl of 50 mM ammonium bicarbonate and 5 mM DTT was added and incubated at 60 °C for 30 min on an Eppendorf thermomixer at a speed of 400 RPM. A volume of 5 μl of 200 mM iodoacetic acid (10 mM) was added to the solution and incubated in the dart at room temperature for 30 min. The solution was diluted to a total volume of 500 μl, and 1:30 trypsin enzyme by weight was added, and the solution was incubated overnight at 37 °C using an Eppendorf thermomixer at a speed of 400 RPM. The trypsinization was stopped with 1% formic acid, and the solution was dried by vacuum centrifugation (SPD1010 Speedvac system, Thermo Savant). The dried sample was resuspended in 2% ACN/water/0.1% TFA and passed through C-18 zip tips (Thermo Fisher Scientific); the cleaned peptides were eluted using 60% ACN/water/0.1% TFA and dried by vacuum centrifugation. The cleaned peptides were further stored at −20 °C until analyzed using LC-MS/MS.

### LC-MS/MS Analysis for HS and CS Disaccharides (Glycomics)

HS and CS disaccharides were analyzed using negative ionization mode electrospray LC-MS as previously described (15). Disaccharides were separated using a 1.9 μm, 0.3 × 150 mm GlycanPac AXH-1 (Thermo Fisher Scientific) column mounted on an Agilent 1200 LC (Agilent Technologies). A 20-min isocratic method was used (85% B) and a flow rate of 7 μl/min (HS) and 5 μl/min (CS). Solvent A was 50 mM ammonium formate pH 4.4 in 10% ACN, and solvent B was 95% ACN/5% water. MS analyses were performed using an Agilent 6520 Q-TOF (Agilent Technologies) using electrospray ionization. An 800 fmol quantity of ΔHexA2S-GlcNCoEt(6S) (Iduron) was added to all the samples as an internal standard before LC-MS analyses. The targeted tandem mass spectrometry (MS/MS) analysis was performed to differentiate between HS and CS disaccharide isoforms (Table 1 and Supplemental Table S1) with a collisional-induced dissociation. A fixed collision energy of 20 was used. The targeted list for HS was *m/z* 458 (charge; z = 1) and *m/z* 247.5 (z = 2), and for CS was *m/z* 458 (z = 1). The relative and absolute abundance were determined using standard curves as previously described (28, 29, 30).

### LC-MS/MS Analysis Peptide (Proteomics)

Nano-LC-MS/MS separation was performed using a nanoAcquity high**-**performance liquid chromatography (Waters Technology) and Q-Exactive mass spectrometer (Thermo Fisher Scientific). A 50 fmol retention time calibration mixture (Pierce) was added to all samples as an internal standard prior to LC-MS analysis. Reversed-phased C-18 analytical (BEH C18, 150 μm × 100 mm) and trapping (180 μm × 20 mm) columns from Waters technology were used with a 120 min method with a gradient from 2 to 98% ACN in 97 min, using 99% water/1% ACN/0.1% formic acid as mobile phase A and 99% ACN/1% water/0.1% formic acid as mobile phase B at a flow rate of 0.5 μl/min as previously described in (30). Data-dependent acquisition MS/MS was acquired in the positive ionization mode for the top ten most abundant precursor ions. Full MS scans were acquired from *m/z* 350 to 2000 with 70,000 resolution using an automatic gain control target of 1e^6^ and a maximum injection time of 100 ms. Dynamic exclusion (12 s) was enabled. The minimum threshold for precursor selection was set to 5 × 10^4^. Precursor ions were fragmented using 2 micro scans at a resolution of 17,500 with a maximum injection time of 50 ms and an automatic gain control value of 2 e^5^ using higher energy collision-induced dissociation with a step-up normalized collision energy of 27, 35.

### Protein Identification, PEAKS Label-free Quantification, and Data Analysis

The raw LC-MS/MS data were converted into mzXML format using ProteoWizard msConvert (31). The data were searched using PeaksDB and PeaksPTM using Peaks Studio version 8.0 (Bioinformatics Solutions, Inc) against the Uniprot/Swissprot database for *Mus musculus* (house mouse) with a 1% false discovery rate (FDR) and at least two unique peptides. A 10-ppm error tolerance for the precursor (MS1) and 0.02 Da mass error tolerance for fragment ions (MS2) were specified. A maximum of three missed cleavages per peptide was allowed for the database search, permitting nontryptic cleavage at one end. Trypsin was specified as the enzyme and carbamidomethylation as a fixed modification. A peaksPTM search was queued after the peaksDB search, using advanced settings of a larger set of variable modifications, including hydroxylation P, oxidation M, hydroxylation K, hydroxylation-Hex K, hydroxylation-Hex-Hex K, HexNAc ST, HexHexNAc ST, phosphorylation STY, ubiquitination K, deamidation N, methoxy K, and nitrotyrosine Y. The final protein list generated was a combination of peaksDB and peaksPTM searches. The label-free quantification was achieved using PEAKS Studio Quantification, a label-free module with a setting of mass error tolerance of 10 ppm and a retention time shift tolerance of 2.0 min. For data filtering for label-free peptide quantification following parameters were used: significance 15, fold change 1, quality 0, average area 1E4, charges from 1 to 10, detected in at least one of 18 samples. The protein quantification following settings was used: significance 0, fold change 1, at least two unique peptides, significance method PEAKS. All Abundances were normalized using TICs by the software relative to saline sample 1 (S1).

### Glycomics Data Analysis Using Excel Spreadsheet

Student's t-tests were performed with two-tailed distribution using Microsoft Excel to test for alterations in the obtained HS and CS disaccharide profiles. Different concentrations (500 fmol, 1 pmol, 2 pmol, 5 pmol, and 10 pmol) of eight HS standard unsaturated disaccharides (D0A0, D2A0, D0A6, D2A6, D0S0, D2S0, D0S6, and D2S6) (Iduron), and four CS unsaturated disaccharides (D0a0, D0a4, D0a6, and D0a10); nomenclature, *m/z*, Lawrence codes and its designation, and other description for the disaccharides are shown in Table 1 and Supplemental Table S1 (4, 5, 6, 7, 8) were run on LC-MS as triplicates, for plotting an MS standard curve. The MS/MS standard curve was plotted for different ratios of HS isoforms (D2A0/D0A6 and D2S0/D0S6) and CS isoforms (D0a4/D0a6) as previously described (8). The area under the curve for extracted ion chromatograms (EICs) for HS and CS disaccharides from each sample was obtained from the raw LC-MS/MS data using qualitative analysis software (version B.06; Agilent Technologies). The obtained abundances for each disaccharide were first normalized to a spiked internal control (as indicated above) and then further normalized to a standard curve to obtain an absolute abundance of HS or CS disaccharides (fmol). A relative abundance was then calculated for each disaccharide from the absolute abundance. For differentiating the isoforms, EIC (MS/MS) for diagnostic ions, as indicated in (32) for each CS and HS disaccharide isoform, was extracted, and abundance was obtained from manual area calculation. The obtained abundance was then used to calculate the % relative abundance of each isoform which was further normalized using the MS/MS standard curve to obtain the % absolute abundance of each HS or CS isoform.

### Statistical Analysis

In order to assess the variability in proteins and GAG disaccharides among the different treatments, we employed several statistical and data visualization methods. We generated heatmaps, hierarchical clustering dendrograms, and principal component analysis (PCA) plots for all LH and ST data and then again for each pair of treatments. We also performed *t* tests to determine significantly differentially expressed proteins for each drug treatment against saline, using a Benjamin–Hochberg corrected *p* value cut-off of 0.2. Protein data was standardized by logging and normalizing in order to fit every sample's protein expression into a normal distribution with mean 0 and SD 1 for sample comparison. GAG data were similarly standardized but without the logging step due to the disaccharide expression already following a normal distribution. All statistical analyses and data visualizations were performed using R (33).To test the significance of the effects of AAV microinjections in mice’s performance in the EPM, one-way ANOVA followed by Tukey post hoc test was used for the total distance, for the percentage of time spent in the open arms and for the percentage of open arms entries. One-way ANOVA followed by Fisher post hoc was used for the latency to the first open arm entry. Two-way ANOVA followed by Tukey post hoc test was used to verify the statistical effects of AAV microinjections in mice’s preference for the cocaine-associated compartment or the novelty-associated one.

### Functional Analysis (Proteomics)

To understand the biological deregulation of aberrantly expressed proteins, the differentially quantified proteins from peaks output were used and further analyzed by in-house and online functional annotation and bioinformatics tools for protein gene ontology (GO) annotation, and pathway analysis, including IPA software (Ingenuity Systems), DAVID Bioinformatics resource 6.8 (https://david.ncifcrf.gov/), and Kyoto Encyclopedia of Genes and Genomes (KEGG) pathways (http://www.genome.jp/kegg/pathway.html). The scores computed by IPA for each network are derived from a *p* value and indicate the likelihood of the proteins being found together in that network by random chance (34). The PCA, clustering plots, and differentially expressed protein lists were obtained from the in-house software PEAKSviz (https://jdhogan.shinyapps.io/peaksviz/).

## Results

### Brain Region–Specific Changes (HS, CS Disaccharide, and Proteins)

Two brain regions, the LH and ST were used for this study. The saline samples from each brain region were used to determine the qualitative brain region–specific changes in HS and CS disaccharides and proteins. The HS and CS disaccharide abundances in the two brain regions with the difference in relative abundance (%) in LH *versus* ST are summarized in Supplemental Table S2. Interestingly, D0A6 and D0A0 HS disaccharides were the most abundant HS disaccharide in LH and ST, respectively, and D0A0 was notably lower in LH *versus* ST. The elevation of D0A6 in LH was an unusual observation, as most brain studies report D0A0 (similar to our ST data) as the most abundant disaccharide (30). To rule out any analytical bias related to sample processing or LC-MS instrument performance, we compared the HS disaccharide profiles of HSBK (heparan sulfate sodium salt from the bovine kidney), a standard control that was processed and acquired on the instrument with both brain region samples at separate times. The EIC for D0A0 and D2A0/D0A6 (Supplemental Fig. S6) for HSBK for both batches showed similar EICs with no significant difference as observed in our samples, ruling out any analytical bias in our data. Sulfated disaccharides (D2A0, D0A6, D2S0, D0S6, D2S6) were higher for LH than ST, pointing toward a higher HS sulfation in LH than ST, which was also confirmed by HS content (%). For CS disaccharides, unsulfated (D0a0) disaccharide was observed to be higher, while sulfated (D0a4/a6) disaccharides were observed to be lower in LH *versus* ST, pointing toward lower CS sulfation in LH *versus* ST contrary to HS sulfation. However, the total CS content (%) was similar in both brain regions. The total CS abundance (fmol) was decreased for LH *versus* ST Importantly, both brain regions showed a higher CS 4-*O* sulfation (D0a4) than 6-*O* sulfation (D0a6).

For proteomics data, a Venn diagram (Supplemental Fig. S7) was constructed using proteins (unique peptide ≥2) observed in saline samples for LH and ST, and a GO and pathway analysis of common and exclusive proteins to each brain region was performed using DAVID. Most proteins in LH were localized in the cytoplasm, whereas most proteins in ST belonged to nonmembrane-bound organelles. The majority of common proteins in both brain regions were localized in mitochondrion. Oxidative phosphorylation was among the top KEGG pathways for ST exclusive and common (LH and ST) proteins, while regulation of actin cytoskeleton was among the top pathway for LH exclusive proteins. It is important to stress that these qualitative brain region–specific trends need further validation with a larger cohort and a dedicated brain region–specific study.

### HS Disaccharide Analysis for LH and ST

HS disaccharides were enzymatically released from tissue lysis of the LH and ST, purified using SEC, and analyzed using a GlycanPac AXH-1 column ESInegative ion MS/MS-based quantitative glycomics. Eight HS unsaturated disaccharides four *N-*acetylated (D0A0;unsulfated, D2A0;2*-O* sulfated, D0A6; 6*-O* sulfated, D2A6; and 2-*O*, 6-*O* sulfated)*,* and four *N*-sulfated (D0S0; no O-sulfation, D2S0; 2-*O* sulfated, D0S6; 6*-O* sulfated, and D2S6; 2-*O*, 6-*O* sulfated) (Table 1) were observed for cocaine (C1-C6), METH (M1, M3-M6; M2 sample was removed for being an outlier sample), and saline (S1-S6) in LH (Supplemental File S1*A*), while only seven HS disaccharides (D0A0, D2A0, D0A6, D0S0, D2S0, D0S6, and D2S6; D2A6 was absent, or present below detectable limits) were observed for cocaine (C1-C6), METH (M1-M6), and saline (S1-S6) in ST (Supplemental File S2*A*). GlcN-*3-0* sulfated and saturated HS disaccharides were not observed as they were below the detectable limit of the instrument used. The disaccharide abundances were normalized to a spiked internal disaccharide (ΔHexA2S-GlcNCoEt(6S)) and MS standard curve. An MS standard curve for different concentrations of eight standard HS disaccharides (D0A0, D2A0, D0A6, D2A6, D0S0, D2S0, D0S6, and D2S6) was plotted for both LH (Supplemental File S1*B*) and ST samples (Supplemental File S2*B*). In addition, an MS/MS standard curve was plotted for different ratios of HS isoforms (D2A0/D0A6 and D2S0/D0S6) for both LH (Supplemental File S1*C*) and ST samples (Supplemental File S2*C*).

**Fig. 1:**
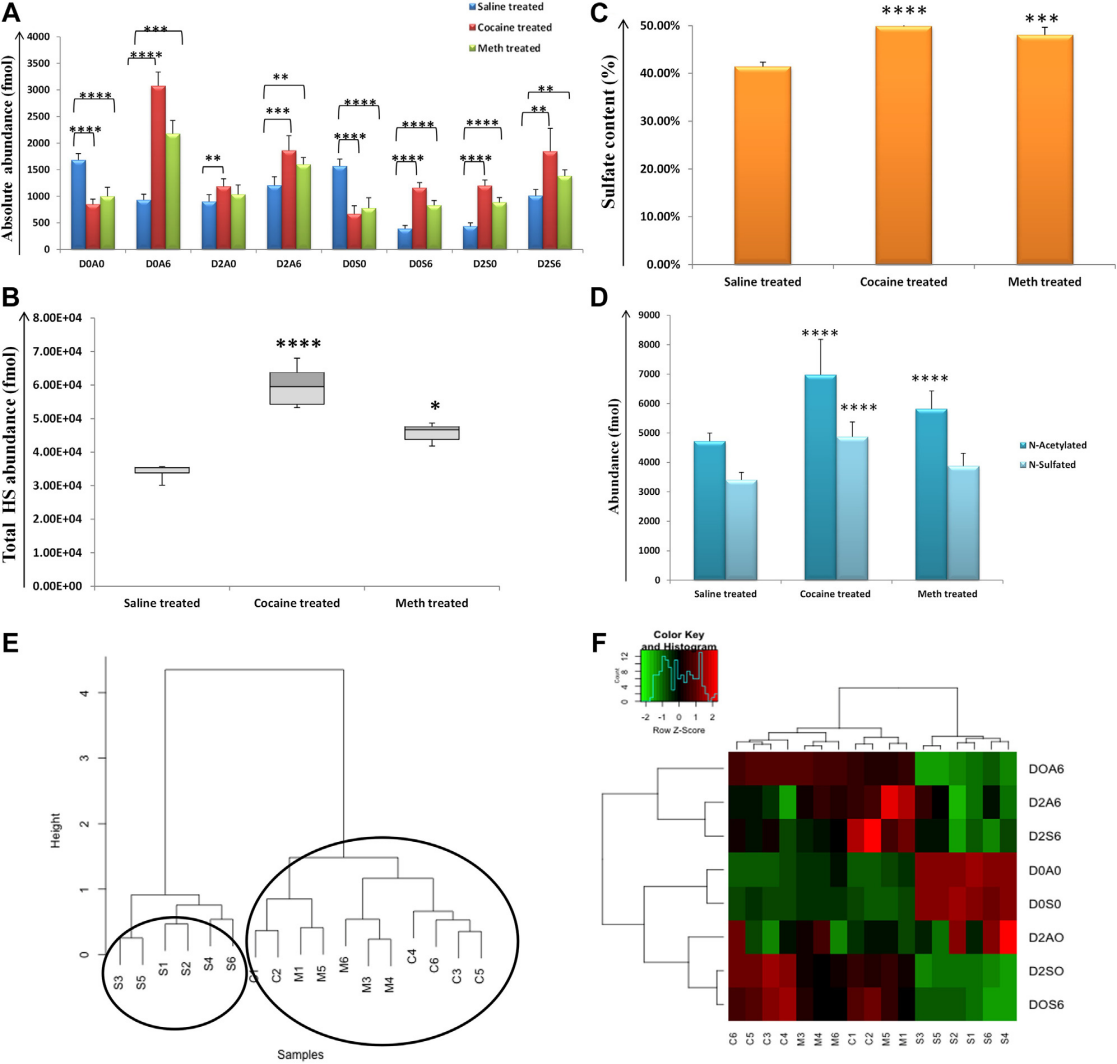
**Heparan sulfate (HS) disaccharides analysis for lateral hypothalamus (LH).***A,* absolute abundances in fmol for eight HS disaccharides (D0A0, D2A0, D0A6, D2A6, D0S0, D2S0, D0S6, and D2S6) for saline (*blue color*), cocaine (*red color*), and METH (*green color*) in LH. *B,* total HS disaccharide abundance (fmol) for saline, cocaine, and METH-treated samples in LH. *C,* sulfate content (%) for saline, cocaine, and METH-treated samples in LH. *D, N*-acetylated and *N*-sulfated HS disaccharide abundance (fmol) for saline-, cocaine-, and METH-treated samples in LH. (∗=*p* ≥ 0.05, ∗∗= *p* ≥ 0.001, ∗∗∗= *p* ≥ 0.0001, ∗∗∗∗= *p* ≥ 0.00001) (mean ± SD). *E,* unsupervised hierarchical clustering of the entire HS disaccharide data for saline, cocaine, and METH samples. *F,* heatmap representation of relative quantification for HS disaccharides (*red*: increased and *green*: decreased abundance). METH, methamphetamine.

For LH, significant changes in the abundances (fmol) of different HS disaccharides were observed for cocaine, METH, and saline-treated samples. Importantly, unsulfated D0A0 (*N*-acetylated) was observed to decrease for drug-treated samples (cocaine; *p* = 1.4 E-07 and METH; *p* = 0.0001) when compared to saline-treated samples. In addition, D0S0 (*N*-sulfated; no *O*-sulfation) HS disaccharide was decreased for cocaine (*p* = 7.5 E-07) and METH (*p* = 0.0001) compared to saline (Fig. 1*A*). Interestingly, D0A6 (*N*-acetylated, 6-*O*-sulfated) HS disaccharide was the most abundant HS disaccharide and was increased for drug-treated samples (cocaine *versus* saline; *p* = 4.46 E-09, METH *versus* saline; *p* = 0.0001). In fact, D0A6 was significantly higher for cocaine-treated than METH-treated samples (*p* = 0.0003). D2A0 (2*-O* sulfated), an isoform of D0A6, was also significantly higher for only cocaine (*p* = 0.004) than saline. The disulfated (D2A6, D2S0, D0S6) and trisulfated (D2S6) HS disaccharides were elevated for cocaine and METH *versus* saline, indicating a higher sulfation pattern in the drug-treated samples.

The total HS abundance (fmol) (Fig. 1*B*) and sulfate content (%) (Fig. 1*C*) were significantly higher for cocaine (*p* = 1.1 E-05, and *p* = 3.8 E-06, respectively) and METH (*p* = 0.02, and *p* = 0.0002, respectively) than saline in LH. Among the drug samples, cocaine was observed to have significantly higher HS abundance (fmol) (*p* = 0.007) than METH in LH. A comparison of *N*-acetylated (D0A0, D2A0, D0A6, D2A6) and *N*-sulfated (D0S0, D2S0, D0S6, D2S6) HS disaccharides revealed *N*-acetylated form to be more abundant than *N*-sulfated for all the samples, and a significant increase in *N*-acetylated HS disaccharides for both cocaine (*p* = 4.13 E-06) and METH (*p* = 0.01) *versus* saline and *N*-sulfated HS disaccharides for cocaine *versus* METH (*p* = 0.006) and saline (*p* = 8.3 E−05) was observed, Figure 1*D*. Unsupervised hierarchical clustering of entire HS disaccharide data for the samples in LH showed a tight clustering of drug samples, while the saline samples were clustered separately, Figure 1*E*. A heatmap representation of relative quantification for HS disaccharides also illustrated the decrease in unsulfated (D0A0, D0S0) and increase in sulfated (D0A6, D2A6, D2S0, D0S6, and D2S6) disaccharides (Fig. 1*F*) in drug *versus* saline-treated samples.

**Fig. 2:**
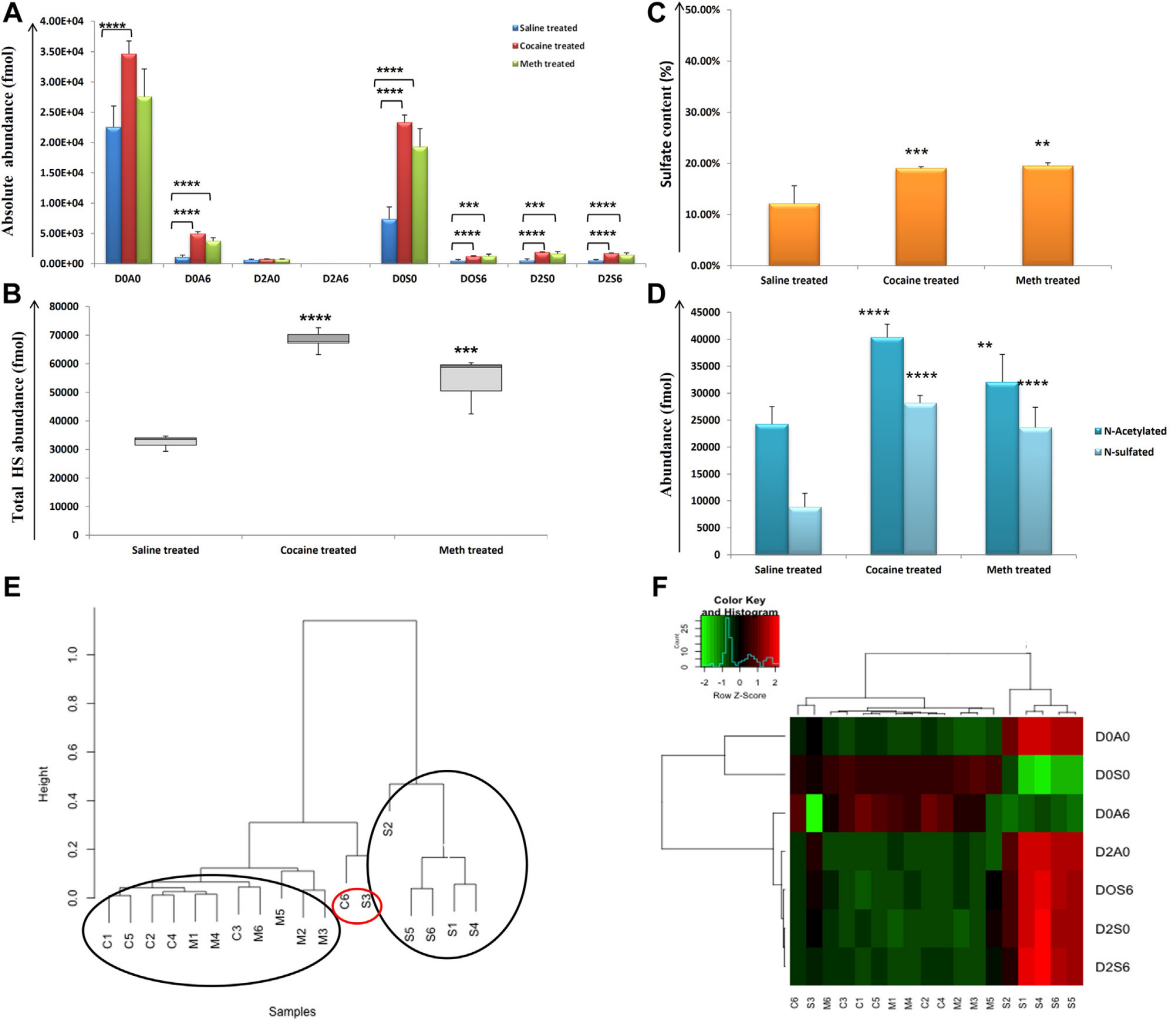
**Heparan sulfate (HS) disaccharides analysis for striatum (ST).***A,* absolute abundance in fmol for seven HS disaccharides (D0A0, D2A0, D0A6, D0S0, D2S0, D0S6, and D2S6) (D2A6 was not detected) for saline (*blue color*), cocaine (*red color*), and METH (*green color*) in ST. *B,* total HS disaccharide abundance (fmol) for saline-, cocaine-, and METH-treated samples in ST. *C,* sulfate content (%) for saline-, cocaine-, and METH-treated samples in ST. *D, N*-acetylated and *N*-sulfated HS disaccharide abundance (fmol) for saline-, cocaine-, and METH-treated samples in ST. (∗=*p* ≥ 0.05, ∗∗= *p* ≥ 0.001, ∗∗∗= *p* ≥ 0.0001, ∗∗∗∗= *p* ≥ 0.00001) (mean ± SD). *E,* unsupervised hierarchical clustering of the entire HS disaccharide data for saline, cocaine, and METH samples. *F,* heatmap representation of relative quantification for HS disaccharides (*red*: increased and *green*: decreased abundance). METH, methamphetamine.

By contrast, for ST, unsulfated D0A0 (*N-*acetylated) was the most abundant HS disaccharide for all the samples and was increased for cocaine (*p* = 2.7 E-05) and METH (*p* = 0.05) *versus* saline samples. D0S0 (*N-*sulfated; no *O*-sulfation) HS disaccharide was the second most abundant HS disaccharide, which was also increased for cocaine (*p* = 1.1E-08) and METH (*p* = 1.06 E-05) *versus* saline samples (Fig. 2*A*). D2A6 disaccharide was absent (or below detectable limits) for ST samples. In agreement with LH, sulfated HS disaccharides were significantly elevated in the drug compared to saline samples for ST. In addition, the total HS abundance (fmol) (Fig. 2*B*) and sulfate content (%) (Fig. 2*C*) were significantly higher for cocaine (*p* = 4.0 E-09, and *p* = 0.0006, respectively) and METH (*p* = 0.0001, and *p* = 0.009, respectively) *versus* saline-treated samples. The *N*-acetylated were more abundant than *N*-sulfated HS disaccharides for cocaine, METH, and saline samples. In addition, both *N*-acetylated and *N*-sulfated HS disaccharides were significantly higher for cocaine (*p* = 2.02 E-0-6, *p* = 1.1 E−08, respectively) and METH (*p* = 0.01, *p* = 1.1 E-05, respectively) than saline samples (Fig. 2*D*). Unsupervised hierarchical clustering of entire HS disaccharide data in ST showed a tight clustering of drug samples, while the saline samples were clustered separately, Figure 2*E*. A heatmap representation of relative quantification for HS disaccharides showed differences in HS disaccharides between drug *versus* saline samples, Figure 2*F*.

### CS Disaccharide Analysis for LH and ST

CS disaccharides were released enzymatically from tissue lysis of the LH and ST, purified using SEC), and analyzed using GlycanPac AXH-1 column ESI-negative ion-MS/MS-based quantitative glycomics. Four CS unsaturated disaccharides D0a0 (unsulfated), D0a4 (*4-O* sulfated), D0a6 (*6-O* sulfated), D0a10 (4*-O* and 6-*O* sulfated) (or D2a6/D2a4; nondifferentiated isoforms with same *m/z* 538; used as D0a10 throughout the manuscript) (Table 1) were observed for cocaine (C1-C6), METH (M1-M6), and saline (S1-S6) in LH (Supplemental File S3*A*) and ST (Supplemental File S4*A*). The trisulfated CS disaccharide D2a10 and saturated CS disaccharides were not observed and/or were below the detectable limit. The CS disaccharides were normalized to a spiked internal disaccharide (ΔHexA2S-GlcNCoEt (6S)) and MS standard curve to obtain an absolute abundance of CS disaccharides. An MS standard curve for different concentrations of four HS disaccharides D0a0, D0a4, D0a6, D0a10) was plotted for both LH (Supplemental File S3*B*-i) and ST samples (Supplemental File S4*B*-i). In addition, an MS/MS standard curve was plotted for different ratios of CS isoforms (D0a4/a6) for both LH (Supplemental File S3*B*-ii) and ST samples (Supplemental File S4*B*-ii).

**Fig. 3:**
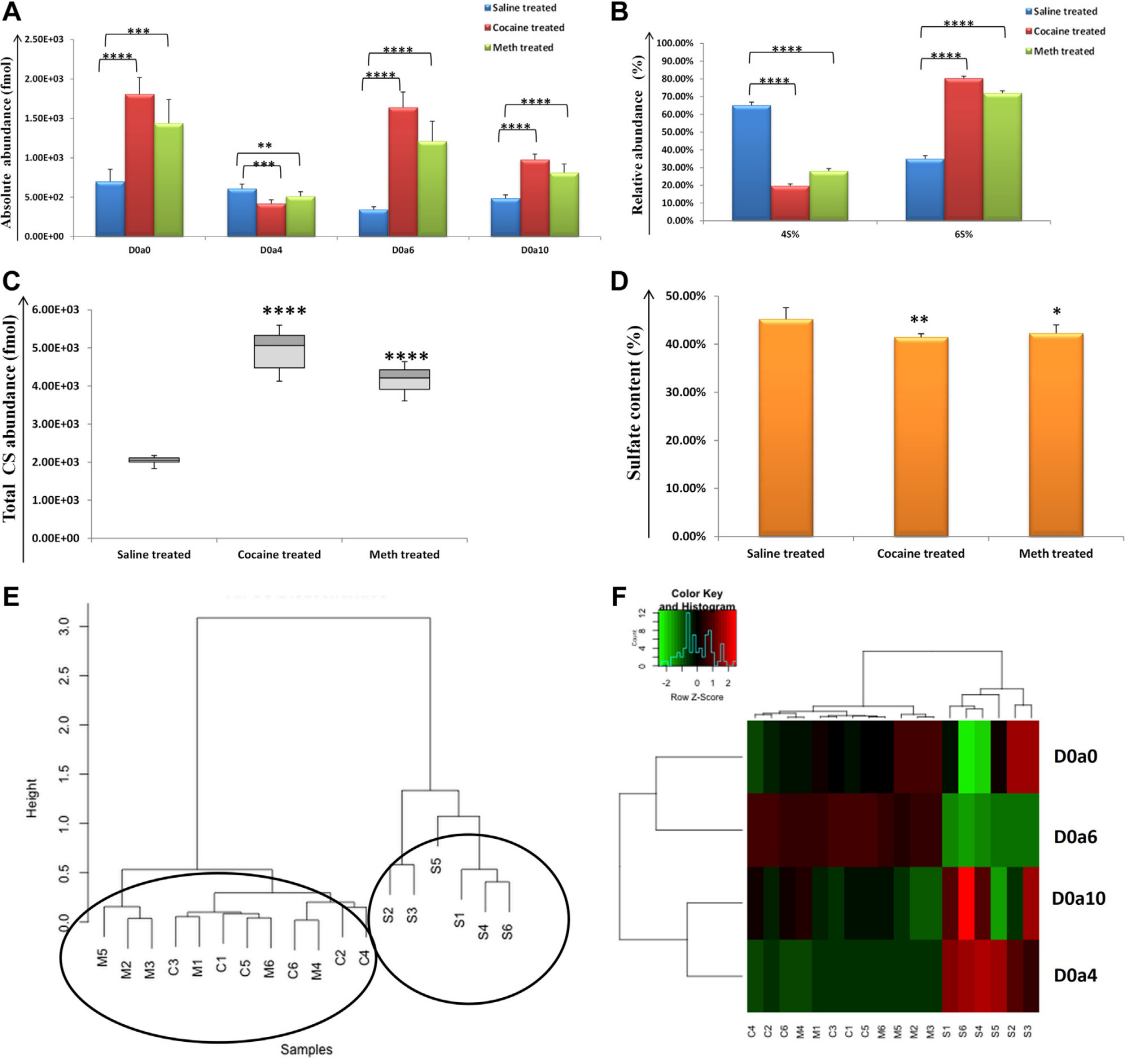
**Chondroitin sulfate (CS) disaccharides analysis for lateral hypothalamus (LH).***A,* absolute abundance in fmol for four CS disaccharides (D0a0, D2a4, D0a6, and D0a10) for saline (*blue color*), cocaine (*red color*), and METH (*green color*) in LH. *B,* relative abundance (%) for 4-*0* sulfation (4S) and 6-*0* sulfation (6S) for saline-, cocaine-, and METH-treated samples in LH. *C,* Total CS abundance in fmol for saline-, cocaine-, and METH-treated samples in LH. *D,* sulfate content (%) for saline-, cocaine-, and METH-treated samples in LH. (∗=*p* ≥ 0.05, ∗∗= *p* ≥ 0.001, ∗∗∗= *p* ≥ 0.0001, ∗∗∗∗= *p* ≥ 0.00001) (mean ± SD). *E,* unsupervised hierarchical clustering of the entire CS disaccharide data for saline, cocaine, and METH samples. *F,* heatmap representation of relative quantification for CS disaccharides (*red:* increased and *green*: decreased abundance). METH, methamphetamine.

For LH, unsulfated D0a0 was the most abundant CS disaccharide for all the samples, and it was significantly elevated for cocaine (C; *p* = 1.2 E-06) and METH (M; *p* = 3.3 E-04) *versus* saline (S)-treated samples (Fig. 3*A*). Interestingly, when compared to D0a4 (4-*O*-sulfation) and D0a6 (6-*O*-sulfation), D0a6 was more abundant in drug-treated samples, while D0a4 was more abundant for saline samples. In addition, a significant increase in D0a6 and a concomitant decrease of D0a4 was observed for cocaine (*p* = 1.9 E-08 and *p* = 0.0001, respectively) and METH (*p* = 8.4 E-06 and *p*= 0.01, respectively) when compared to saline samples. The disulfated CS disaccharide D0a10 was also significantly increased for cocaine (*p* = 6.8 E-08) and METH (*p* = 5.8 E-05) relative to saline samples. The unique differential expression of 4-*O* (4S) and 6*-0* (6S) sulfation was also illustrated by the relative abundance (%) of 4S *versus* 6S, where 6S was relatively higher, while 4S was significantly lower for cocaine (*p*= 2.1 E-13) and METH (*p*= 2.8 E-12) than saline samples (Fig. 3*B*). Unsupervised hierarchical clustering of entire CS disaccharide data in LH showed a tight clustering of drug samples, while the saline samples were clustered separately, Figure 3*E*. A heatmap representation of relative quantification for CS disaccharides also highlighted the increased D0a6 and increased D0a4, Figure 3*F*, in drug-treated *versus* saline samples. The total CS abundance (fmol) was observed to increase for drug-treated samples (cocaine; *p* = 6.6 E-07, and METH; *p* = 4.6 E-07) compared to saline samples in LH (Fig. 3*C*), whereas sulfate content (%) was observed to decrease for drug-treated samples (cocaine; *p =* 0.004, and METH; *p* = 0.03) compared to saline samples (Fig. 3*D*).

**Fig. 4:**
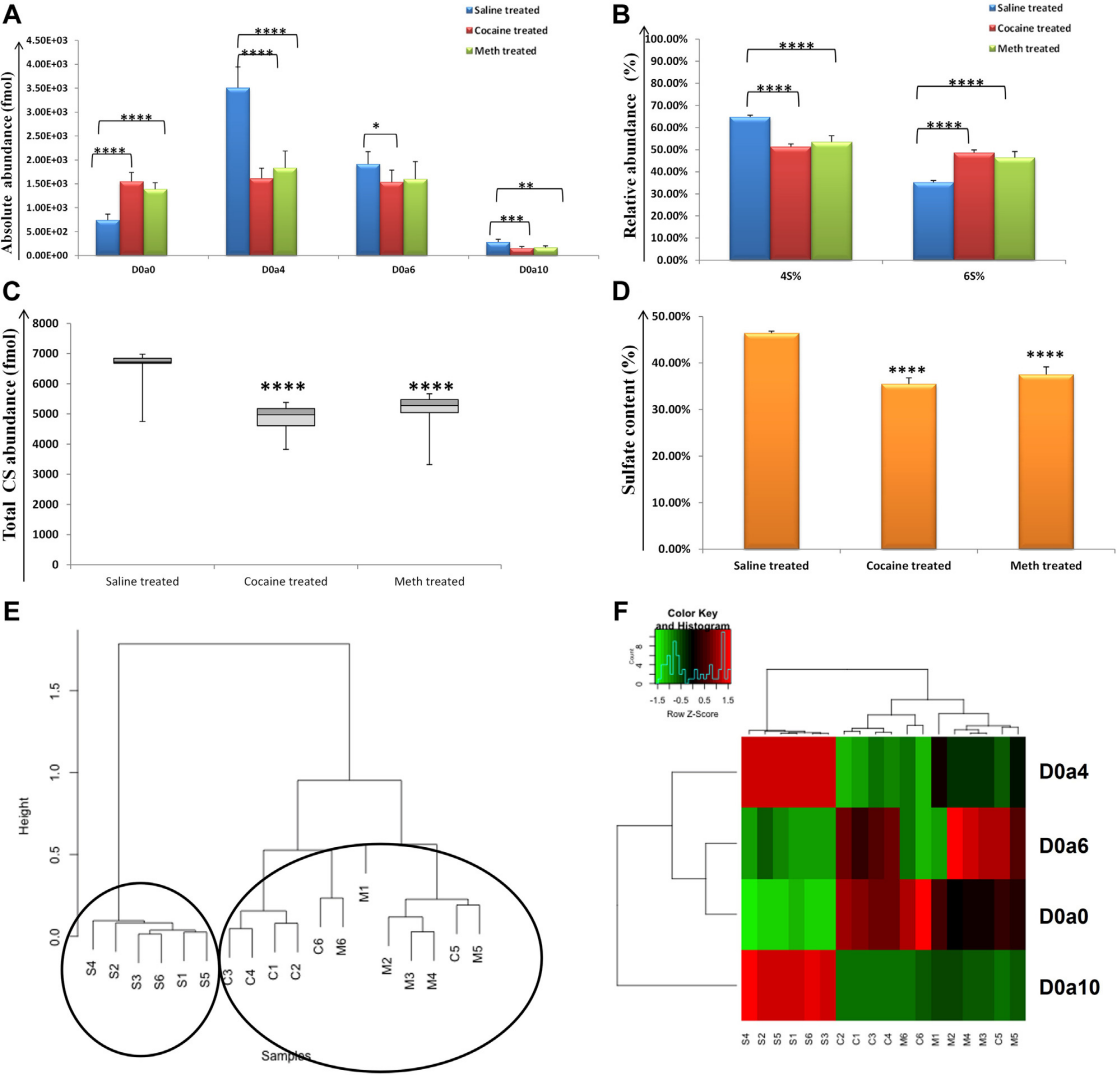
**Chondroitin sulfate (CS) disaccharides analysis for striatum (ST).***A,* absolute abundance in fmol for four CS disaccharides (D0a0, D2a4, D0a6, and D0a10) for saline (*blue color*), cocaine (*red color*), and METH (*green color*) in ST. *B,* relative abundance (%) for 4-*0* sulfation (4S) and 6-*0* sulfation (6S) for saline-, cocaine-, and METH-treated samples in ST. *C,* total CS abundance in fmol for saline-, cocaine-, and METH-treated samples in ST. *D,* sulfate content (%) for saline-, cocaine-, and METH-treated samples in ST. (∗=*p* ≥ 0.05, ∗∗= *p* ≥ 0.001, ∗∗∗= *p* ≥ 0.0001, ∗∗∗∗= *p* ≥ 0.00001) (mean ± SD). *E,* unsupervised hierarchical clustering of the entire CS disaccharide data for saline, cocaine, and METH samples. *F,* heatmap representation of relative quantification for CS disaccharides (*red:* increased and *green:* decreased abundance). METH, methamphetamine.

For ST, D0a4 was the most abundant CS disaccharide, similar to LH, and was significantly decreased for cocaine (*p* = 2.38 E-06) and METH (*p* = 2.6 E-05) compared to saline samples. However, unlike LH, D0a6 was also decreased for cocaine *(p* = 0.03) and METH (*p* = 0.1; not significant) *versus* saline samples (Fig. 4*A*). The unsulfated D0a0 was increased for cocaine *(p* = 4.8 E-06) and METH (*p* = 6.1 E-06) when compared to saline-treated samples, and the disulfated D0a10 was also significantly decreased for cocaine (*p* = 0.0009), and METH (*p* = 0.002) relative to saline-treated samples in ST. The unique differential expression of 4-*O* (4S) and 6*-0* (6S) sulfation was also illustrated by the relative abundance (%) of 4S *versus* 6S, where 6S was relatively higher, while 4S was lower for cocaine (*p* = 8.8 E−10) and METH (*p*= 2.5 E-06) than saline samples in ST (Fig. 4*B*). The total CS abundance (fmol) was observed to decrease for drug-treated samples (cocaine; *p* = 4.8 E-06, and METH; *p* = 6.1 E-06) compared to saline samples in ST, unlike LH (Fig. 4*C*). While similar to LH, sulfate content (%) was observed to decrease for drug-treated samples (cocaine; *p* = 2.78 E-09, and METH; *p* = 0.04) compared to saline samples (Fig. 4*D*). Unsupervised hierarchical clustering of entire CS disaccharide data in ST showed a tight clustering of drug samples, while the saline samples were clustered separately, Figure 4*E*. A heatmap representation of relative quantification for CS disaccharides also showed increased D0a6 and increased D0a4, Figure 4*F*, in drug-treated *versus* saline-treated samples.

### Proteomics Analysis for LH and ST

Two different drug treatments, cocaine and METH, and a saline treatment as control were performed as six biological sets for each (cocaine C1-C6, METH M1-M6, and saline S1-S6), for two brain regions LH and ST, and were analyzed by reversed-phase nano-LC-MS/MS. For LH, two technical replicates and for ST, one technical replicate for each biological sample were acquired and processed using PEAKS software v8.0. About 900 to 1000 and 700 to 800 proteins with two unique peptides for LH (Supplemental File S5) and ST (Supplemental File S6), respectively, were observed for combined six biological (and technical replicates) for each treatment after PEAKS PTM analysis. A list of proteoglycans and their interacting and binding partners identified in the proteomics data for cocaine, METH, and saline from both LH and ST are shown in Supplemental Table S3.

A Venn diagram (http://bioinfogp.cnb.csic.es/tools/venny/) was constructed for a combined protein list from six biological replicates for each cocaine, METH, and saline for LH, and ST (Supplemental Fig. S8, *A* and *B*), to visualize common and exclusive proteins between the three groups. Interestingly, the majority of proteins 766 for LH and 607 for ST were common between the three groups. Some unique proteins were observed for cocaine (67), METH (84), and saline (69) for LH, but no unique proteins were present for cocaine and saline, and only METH (117) showed some unique proteins for ST. A GO annotation analysis of the complete protein list from all samples from LH and ST was performed using DAVID Bioinformatics resource 6.8 (https://david.ncifcrf.gov/). Supplemental Fig. S8 shows the top, C, GO cellular components; D, GO biological process; E, GO molecular function; F, KEGG pathway analysis; for LH; left panel, and ST; right panel. Among the top GO annotations (based on higher % protein) were cytoplasm as the cellular component, transport as the biological process, protein binding as the molecular function, and metabolic pathways for KEGG pathway analysis. This was true for all three groups and both brain regions. Protein binding and metabolic pathways have been reported among top molecular function and KEGG pathway analysis, respectively, in the prefrontal cortex after repeated cocaine exposure (35).

Using the label-free quantification data from PEAKS Studio Quantification label-free module for LH (Supplemental File S7*A*) and ST (Supplemental File S7*B*), and an R program (33), an unsupervised clustering and PCA plots for complete proteomics data for six biological replicates of cocaine (C1-C6), METH (M1-M6), and saline (S1-S6) for both LH (Supplemental Fig. S9, *A* and *B*) and ST (Supplemental Fig. S9, *C* and *D*) were plotted. As shown in Supplemental Fig. S9, a tight clustering of drug-treated biological samples, that is, cocaine and METH, were observed, while saline samples were clustered separately. In addition, saline biological replicates had higher intra-similarity as than drug biological replicates, and among biological replicates for drug treatments, cocaine samples were sparser than METH samples. In addition, ST showed much closer clustering for drug samples than LH.

A *t* test was performed on the label-free quantified data to obtain differentially expressed proteins; a Benjamin–Hochberg corrected *p* value cut-off of 0.2 was used to determine significant proteins. A pairwise *t* test (cocaine *versus* saline and METH *versus* saline) was performed to obtain increased and decreased abundance proteins for LH (Supplemental File S8-i) cocaine *versus* saline, ii) METH *versus* saline) and ST (Supplemental File S9-i) cocaine *versus* saline, ii) METH *versus* saline). Using filtering parameters such as Bonferroni value of <1 and an FDR <0.02, 54 (33 increased, and 21 decreased) and 96 (41 increased, and 55 decreased), differentially expressed proteins were observed for cocaine *versus* saline and METH *versus* saline, respectively in LH, and 376 (202 increased, and 174 decreased) and 403 (232 increased, and 171 decreased) differentially expressed proteins were observed for cocaine *versus* saline and METH *versus* saline, respectively in ST. The top ten increased and decreased abundance proteins with their respective *t* values (value from the *t* test which is similar to the difference between the means of the two groups or the log of the fold change. A negative *t,*value represents the lower expression, while a positive *t* value represents higher expression in the first group, for cocaine *versus* saline and METH *versus* saline for both LH (A) and ST (B) are shown in Table 2. Interestingly, a number of common differentially expressed proteins were observed for both drug *versus* saline, including increased cytochrome c oxidase subunit 6A1, ubiquitin thioesterase, histone H1.4, and NADH dehydrogenase (ubiquinone) 1 beta subcomplex subunit 6, and decreased tenascin-R (TENR), synapsin-2 (SYN2), and synaptophysin in LH, and increased DnaJ homolog subfamily C member 5, ubiquitin-2, and prefoldin subunit 2, and decreased myelin proteolipid protein (MYPR), syntaxin-binding protein 1, and Ras-related protein in ST. Some proteins had an opposite expression in the two brain regions, for example, Thy-1 membrane glycoprotein, which was decreased for METH in LH but was increased for cocaine in ST when compared to saline samples. Intriguingly, CSPGs, neurocan (NCAN), and CSPG5 were observed among increased abundance proteins for both cocaine and METH *versus* saline in ST, Table 2, and Supplemental File S9.

**Table 2:** Differentially (increased abundance-i and decreased abundance-ii) proteins for lateral hypothalamus (LH) and striatum (ST) for pairwise comparison, that is, cocaine *versus* saline (*left panel*) and methamphetamine (METH) *versus* saline (*right panel*)

Protein accession	Protein name	t	Protein accession	Protein name	t
Lateral hypothalamus (LH)					
i) Top ten increased abundance cocaine *versus* saline			i) Top ten increased abundance METH *versus* saline		
P43024|CX6A1_MOUSE	Cytochrome c oxidase subunit 6A1, mitochondrial	11.7	P43024|CX6A1_MOUSE	Cytochrome c oxidase subunit 6A1, mitochondrial	24.9
P97450|ATP5J_MOUSE	ATP synthase-coupling factor 6, mitochondrial	11.2	Q7TQI3|OTUB1_MOUSE	Ubiquitin thioesterase OTUB1	17.2
Q7TQI3|OTUB1_MOUSE	Ubiquitin thioesterase OTUB1	9.8	P43274|H14_MOUSE	Histone H1.4	15.8
Q3UIU2|NDUB6_MOUSE	NADH dehydrogenase [ubiquinone] 1 beta subcomplex subunit 6	9.5	O35658|C1QBP_MOUSE	Complement component 1 Q subcomponent-binding protein, mitochondrial	13.3
Q9CPQ1|COX6C_MOUSE	Cytochrome c oxidase subunit 6C	9.4	Q8CCT4|TCAL5_MOUSE	Transcription elongation factor A protein-like 5	11.7
P02088|HBB1_MOUSE	Hemoglobin subunit beta-1	8.4	Q9CQJ8|NDUB9_MOUSE	NADH dehydrogenase [ubiquinone] 1 beta subcomplex subunit 9	11.3
P43274|H14_MOUSE	Histone H1.4	8.5	Q91WS0|CISD1_MOUSE	CDGSH iron-sulfur domain-containing protein 1	11.2
Q9CQZ6|NDUB3_MOUSE	NADH dehydrogenase [ubiquinone] 1 beta subcomplex subunit 3	8.4	O55126|NIPS2_MOUSE	Protein NipSnap homolog 2	11.1
O08749|DLDH_MOUSE	Dihydrolipoyl dehydrogenase, mitochondrial	8.2	Q3UIU2|NDUB6_MOUSE	NADH dehydrogenase [ubiquinone] 1 beta subcomplex subunit 6	10.7
Q9CQH3|NDUB5_MOUSE	NADH dehydrogenase [ubiquinone] 1 beta subcomplex subunit 5, mitochondrial	8.1	Q8K1M6|DNM1L_MOUSE	Dynamin-1–like protein	10.5
ii) Top ten decreased abundance cocaine *versus* saline			ii) Top ten decreased abundance METH *versus* saline		
Q9JIA1|LGI1_MOUSE	Leucine-rich glioma-inactivated protein 1	−10.6	Q91VD9|NDUS1_MOUSE	NADH-ubiquinone oxidoreductase 75 kDa subunit, mitochondrial	−10.6
P56695|WFS1_MOUSE	Wolframin	−9.2	P04104|K2C1_MOUSE	Keratin, type II cytoskeletal 1	−10.4
Q80TL4|PHF24_MOUSE	PHD finger protein 24	−7.7	P16330|CN37_MOUSE	2′,3′-cyclic-nucleotide 3′-phosphodiesterase	−9.9
Q9DBG3|AP2B1_MOUSE	AP-2 complex subunit beta	−7.2	P68372|TBB4B_MOUSE	Tubulin beta-4B chain	−9.5
P11798|KCC2A_MOUSE	Calcium/calmodulin-dependent protein kinase type II subunit alpha	−6.8	P17182|ENOA_MOUSE	Alpha-enolase	−9.1
Q60829|PPR1B_MOUSE	Protein phosphatase 1 regulatory subunit 1B	−6.6	P01831|THY1_MOUSE	Thy-1 membrane glycoprotein	−9.1
Q9JME5|AP3B2_MOUSE	AP-3 complex subunit beta-2	−6.2	P18242|CATD_MOUSE	Cathepsin D	−8.1
Q5SQX6|CYFP2_MOUSE	Cytoplasmic FMR1-interacting protein 2	−5.8	Q7TMM9|TBB2A_MOUSE	Tubulin beta-2A chain	−7.9
Q62277|SYPH_MOUSE	Synaptophysin	−5.7	P48962|ADT1_MOUSE	ADP/ATP translocase 1	−7.6
Q9Z1S5|SEPT3_MOUSE	Neuronal-specific septin-3	−5.7	P01942|HBA_MOUSE	Hemoglobin subunit alpha	−7.5
Striatum (ST)					
i) Top ten increased abundance cocaine *versus* saline			i) Top ten increased abundance METH *versus* saline		
P60904|DNJC5_MOUSE	DnaJ homolog subfamily C member 5	28.9	P60904|DNJC5_MOUSE	DnaJ homolog subfamily C member 5	30.4
Q9WV98|TIM9_MOUSE	Mitochondrial import inner membrane translocase subunit Tim9	22.3	Q9QZM0|UBQL2_MOUSE	Ubiquilin-2	28.1
P63323|RS12_MOUSE	40S ribosomal protein S12	21.5	O70591|PFD2_MOUSE	Prefoldin subunit 2	24.9
P01831|THY1_MOUSE	Thy-1 membrane glycoprotein	20.8	Q9WV98|TIM9_MOUSE	Mitochondrial import inner membrane translocase subunit Tim9	23.5
Q9CWM4|PFD1_MOUSE	Prefoldin subunit 1	20.4	Q9CWM4|PFD1_MOUSE	Prefoldin subunit 1	20.3
P56212|ARP19_MOUSE	cAMP-regulated phosphoprotein 19	20.0	P63323|RS12_MOUSE	40S ribosomal protein S12	19.8
O70591|PFD2_MOUSE	Prefoldin subunit 2	17.8	P56212|ARP19_MOUSE	cAMP-regulated phosphoprotein 19	19.1
Q9QZM0|UBQL2_MOUSE	Ubiquilin-2	17.5	Q9D1X0|NOL3_MOUSE	Nucleolar protein 3	18.8
Q8R317|UBQL1_MOUSE	Ubiquilin-1	17.1	P55066|NCAN_MOUSE	Neurocan core protein	18.7
Q9D8Z2|TRIA1_MOUSE	TP53-regulated inhibitor of apoptosis 1	16.3	P63158|HMGB1_MOUSE	High mobility group protein B1	17.8
ii) Top ten decreased abundance cocaine *versus* saline			Top ten decreased abundance METH *versus* saline		
P60202|MYPR_MOUSE	Myelin proteolipid protein	−21.5	Q9R1Q9|VAS1_MOUSE	V-type proton ATPase subunit S1	−32.2
P52480|KPYM_MOUSE	Pyruvate kinase PKM	−18.9	Q9D6M3|GHC1_MOUSE	Mitochondrial glutamate carrier 1	−23.5
Q61885|MOG_MOUSE	Myelin-oligodendrocyte glycoprotein	−17.8	Q9CQQ7|AT5F1_MOUSE	ATP synthase F(0) complex subunit B1, mitochondrial	−23.5
P68368|TBA4A_MOUSE	Tubulin alpha-4A chain	−17.4	P60202|MYPR_MOUSE	Myelin proteolipid protein	−20.4
P63011|RAB3A_MOUSE	Ras-related protein Rab-3A	−16.3	P63011|RAB3A_MOUSE	Ras-related protein Rab-3A	−19.1
O08599|STXB1_MOUSE	Syntaxin-binding protein 1	−15.8	O08599|STXB1_MOUSE	Syntaxin-binding protein 1	−18.9
Q9D0K2|SCOT1_MOUSE	Succinyl-CoA:3-ketoacid coenzyme A transferase 1, mitochondrial	−15.6	Q8VDN2|AT1A1_MOUSE	ATP synthase F(0) complex subunit B1, mitochondrial	−18.4
O88569|ROA2_MOUSE	Heterogeneous nuclear ribonucleoproteins A2/B1	−15.0	P52480|KPYM_MOUSE	Pyruvate kinase PKM	−18.2
P06151|LDHA_MOUSE	L-lactate dehydrogenase A chain	−14.7	P62827|RAN_MOUSE	GTP-binding nuclear protein Ran	−17.6
P62806|H4_MOUSE	Histone H4	−14.5	Q9DBG3|AP2B1_MOUSE	AP-2 complex subunit beta	−17.1

A negative *t* value represents the lower expression, while a positive *t* value represents the higher expression in the first group. t = *t* value, a value from the *t* test which is similar to the difference between the means of the two groups or the log of the fold change.

**Fig. 5:**
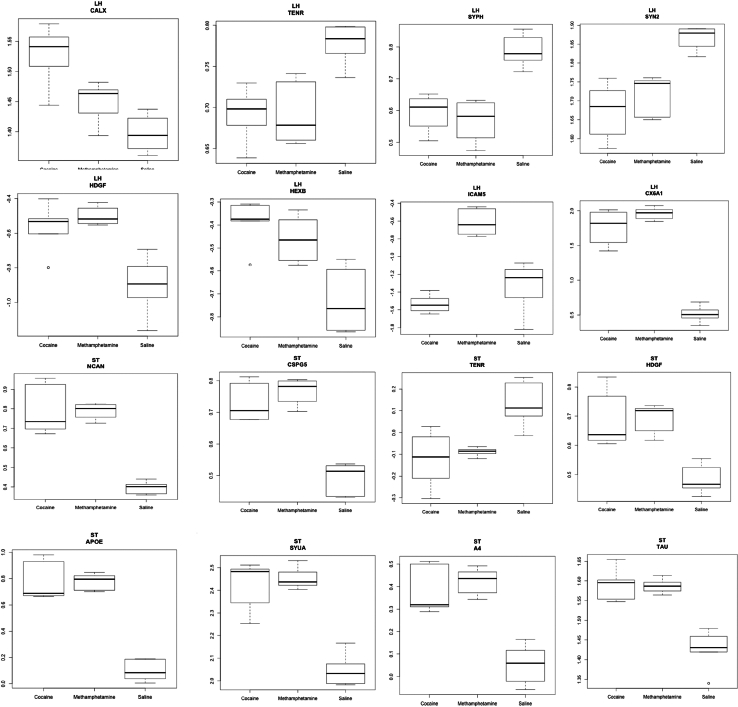
**Box plots for differentially expressed proteins.** A box-plot representation of differentially expressed proteins after ANOVA analysis for cocaine, methamphetamine, and saline in the lateral hypothalamus (LH) (first and second row) and striatum (ST) (third and fourth row). The *y-*axis represents logged and scaled abundance of each protein.

A three-way ANOVA was also performed to obtain a list of differentially expressed proteins in the three treatments: cocaine, METH, and saline (LH; Supplemental File S8-iii, and ST; Supplemental File S9-iii). For LH, 97 differentially expressed proteins were observed using a Bonferroni value of <1 and an FDR< 0.2, including cytochrome c oxidase subunit 6A1, ubiquitin thioesterase, NADH dehydrogenase 1 beta subcomplex subunit 6, NADH dehydrogenase [ubiquinone] iron-sulfur protein 1 mitochondrial, intercellular adhesion molecule 5, calcium/calmodulin-dependent protein kinase type II subunit alpha and beta, cytochrome c oxidase subunit 6C, synaptophysin, SYN2, calnexin (CALX), hepatoma-derived growth factor (HDGF), neurogranin, neurofilament medium polypeptide, beta-hexosaminidase subunit beta, and TENR. For ST, 451 differentially expressed proteins were observed, including calcium/calmodulin-dependent protein kinase type II subunit alpha, DnaJ homolog subfamily C member 5, ubiquitin-2, MYPR, cytochrome c oxidase subunit 2, CALX, APO-E, APO-D, alpha-synuclein (SYUA), microtubule-associated protein tau (TAU), SYN2, amyloid-beta A4 protein (A4), calreticulin, annexin A6 and A7, and HDGF. Figure 5 shows a box plot representation of the logged and scaled abundances of some of the important proteins for the three treatments in LH and ST.

Proteomics data for the two brain regions were used to determine characteristic features defined by proteins that were differentially expressed in the same direction (both with *t* value >0 or *t* value <0) and FDR <0.2 in both brain regions LH and ST for cocaine *versus* saline and METH *versus* saline comparisons. Using this criterion, 66 and 135 common differentially expressed proteins were observed for both brain regions for cocaine *versus* saline (Supplemental File S10-i) and METH *versus* saline (Supplemental File S10-ii), respectively, and 43 proteins (Supplemental File S10-iii), including, MYPR, KCC2A, SYN2, TENR, CALX, ANXA7, and HDGF were found to overlap for both drug treatments (cocaine and METH) *versus* saline, common to both brain regions (LH and ST). A KEGG pathway analysis of these 43 common proteins using DAVID revealed previously reported oxidative phosphorylation as the top aberrant pathway for drug abuse, as shown in Supplemental Fig. S10. An ingenuity pathway analysis (IPA) on the 43 proteins illustrated neurological disease (*p* = 2.19 E-02-8.53 E-07; 24 molecules), organismal injury and abnormalities (*p* = 2.31 e-02-8.53 e07; 42 molecules), and developmental disorders (*p* = 2.12 E-02-3.20E-05; 9 molecules) among top diseases and disorders (IPA summary, Supplemental PDF S2). Among the top networks were cellular assembly and organization, cellular function and maintenance, and cellular movement (IPA score 47), Supplemental Fig. S11. The network was overlayed with diseases and biological functions (nervous system and development function, neurological disease, developmental disorder, and psychological disorder; pink border) and canonical pathways (opioid signaling pathway, clathrin-mediated endocytosis signaling, synaptic long-term potentiation, and synaptogenesis signaling pathway).

### Increasing CS *4-0* Levels in the LH Ameliorates Anxiety and Prevents the Expression of Preference for Cocaine in a Novelty-Induced Conditioned Place Preference Test during Withdrawal

**Fig. 6:**
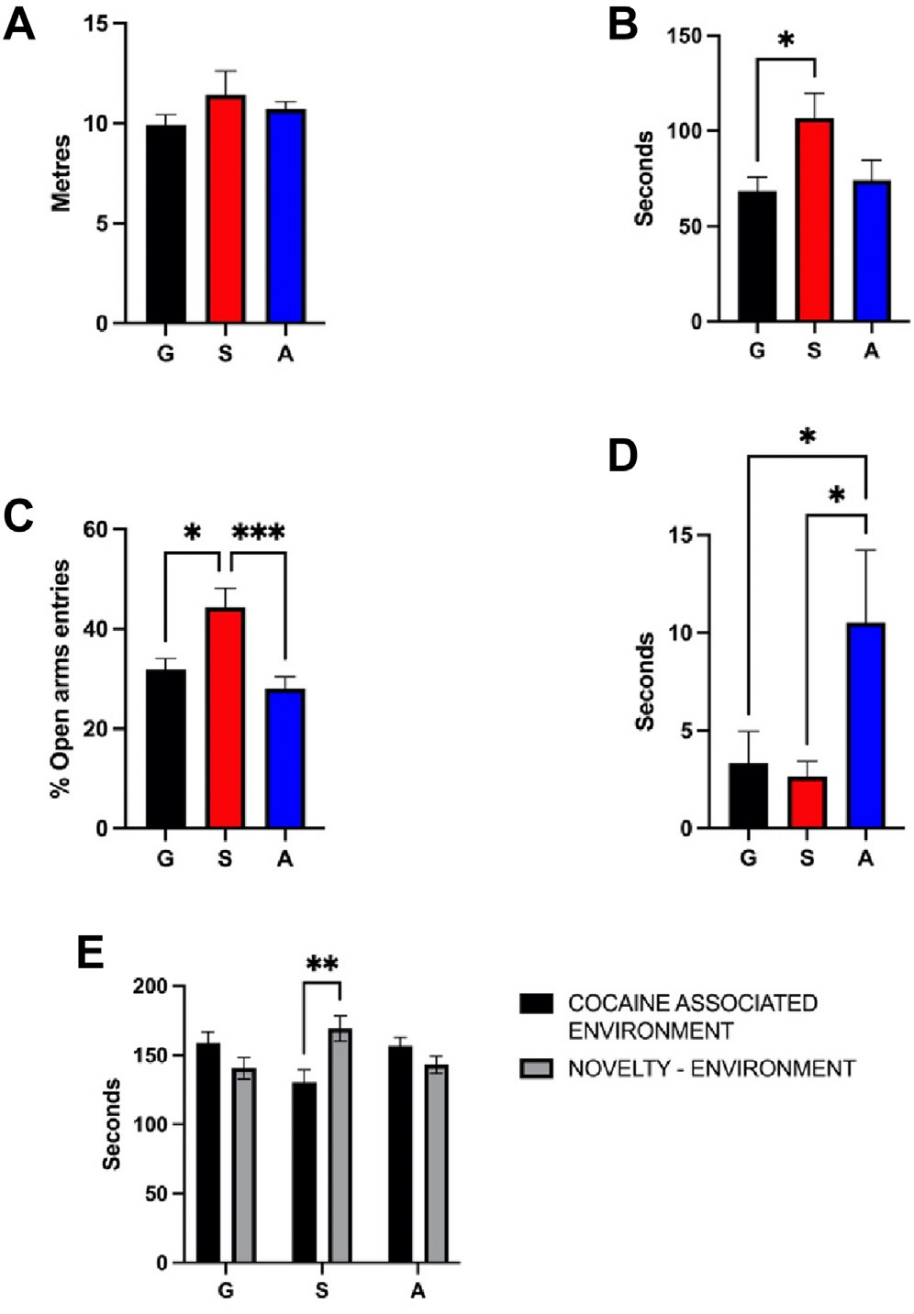
**Effects of downregulating by shRNA (S), overexpressing (A) ARSB, and control group (G) in mice’s anxiety in the elevated plus maze and in mice’s preference for cocaine over novelty during cocaine withdrawal.***A*, the total activity of the control group (G), the shRNA ARSB group (S), and the upregulated ARSB group (A) during the test. *B*, one-way ANOVA revealed a significant difference between the groups in the time spent in the open arms. *C*, the percentage of entries in the open arms. *D*, the latency to the first entry in the open arms (F_2,42_ = 3.88, *p* < 0.05; F_2,42_ = 8.73, *p* < 0.001; F_2,42_ = 3.36, *p* < 0.05). Tukey post hoc revealed that S spent significantly more time in the open arms when compared to the control (G) (*p* < 0.05) and had a significantly higher percentage of entries in the open arms than G (*p* < 0.05) and *A* (*p* < 0.001). Moreover, Fisher post hoc revealed that *A* had a significantly higher latency to the first entry in the open arms than G (*p* < 0.05) and S (*p* < 0.05). In addition, two-way ANOVA revealed a significant effect of Group x Novelty interaction (F2,90 = 8.34, *p* < 0.001) in mice’s preference between cocaine and novelty. *E*, Tukey post hoc revealed that S significantly spent more time exploring the novelty (*p* < 0.01). ARSB, arylsulfatase B.

We used AAV to overexpress ARSB (N-acetylgalactosamine-4-sulfatase) or an shRNA to ARSB, while an empty AAV was used for control. Figure 6, *A*–*D* represents the effects of overexpressing or downregulating LH ARSB by shRNA in mice’s performance at the EPM after 10 days of cocaine withdrawal following an intraperitoneal treatment with cocaine (10 mg/kg/10 ml) for 14 days Figure 6*A* represents the total activity of the control group (G), the shRNA ARSB group (S), and the upregulated ARSB group (A) during the test. One-Way ANOVA did not reveal any significant effect (*p* > 0.05), showing that mice were not sedate nor hyperactive, hence validating the test. Anxiety-related parameters were significantly different. Accordingly, one-way ANOVA revealed a significant difference between the groups in the time spent in the open arms (Fig. 6*B*), in the percentage of entries in the open arms (Fig. 6*C*), and in the latency to the first entry in the open arms (Fig. 6*D*) (F_2,42_ = 3.88, *p* < 0.05; F_2,42_ = 8.73, *p* < 0.001; F_2,42_ = 3.36, *p* < 0.05). Moreover, Tukey post hoc test revealed that S-group spent significantly more time in the open arms when compared to the G-group (*p* < 0.05) and had a significantly higher percentage of entries in the open arms than G (*p* < 0.05) and A (*p* < 0.001). Furthermore, Fisher post hoc revealed that A had a significantly higher latency to the first entry in the open arms than G (*p* < 0.05) and S (*p* < 0.05). In addition, Figure 6*E* represents the effects of overexpressing or downregulating LH ARSB in mice’s preference between cocaine and novelty in a novelty-induced place preference test during cocaine withdrawal. Two-way ANOVA revealed a significant effect of Group x Novelty interaction (F_2,90_ = 8.34, *p* < 0.001). Moreover, Tukey post hoc revealed that S significantly spent more time exploring the novelty during withdrawal (*p* < 0.01) when compared to G and A. Altogether, these results point out how restoring cocaine-induced reduction of C4-S0, through silencing ARSB RNA, reduced anxiety and preference for cocaine in S mice during cocaine withdrawal. To verify the efficiency of AAV injections, we used MS CS disaccharide analysis to capture the changes in *4-O* and *6-O* sulfation for overexpressing ARSB (N-acetylgalactosamine-4-sulfatase) or to knock it down through shARSB and GFP as control (Supplemental Fig. S4). A significant increase in CS *4-0* levels in the LH by AAV delivery of an shRNA to ARSB was observed.

## Discussion

Aberration in protein and gene expression in neuropsychiatry has been well documented, but only limited studies have utilized MS-based proteomics to profile altered proteins related to drug abuse while profiling of glycoconjugates (HS and CS) remains unexplored. This study provides in-depth profiling of HS and CS disaccharides, as well as of the proteome of two essential brain regions involved in drug abuse, the LH, and the ST harvested from mice treated with two classic brain stimulants, cocaine and METH. Comprehensive glycomics and proteomics profiles of drugs *versus* saline treatments were generated, underpinning the structural aberration of these biomolecules associated with these drugs of abuse.

We found that both stimulants induce significant changes in HS and CS disaccharide profiles when for drug treatment compared to saline treatment. A key observation was an increase in total HS abundance and sulfate content in both brain regions indicating a potential increase in heparan sulfate proteoglycans (HSPGs) and sulfotransferase enzymes (or a decrease in sulfatase) associated to the treatment with both drugs. Accordingly, our previous study showed an increase in gene expression of HSPG syndecan-3 in LH after excessive cocaine self-administration (12). In aggrement with our findings, another study showed downregulation of *Sulf1*, the extracellular *6-O-*endosulfatases in mice nucleus accumbens after treatment with cocaine (36). The same study also reported a decrease in NDST4, a bifunctional enzyme that catalyzes *N*-deacetylation and *N*-sulfation in HS, congruent with our observation of an increase in HS *N-*acetylation and decreases in *N*-sulfation in drug treatments (36).

Another interesting observation was an increase in total CS abundance for LH and a decrease in CS sulfate content for LH and ST in drug-treated *versus* saline samples. Conversely, CS sulfation was observed to increase with ethanol treatment in astrocytes (37). A major finding of this study was a significant increase in CS *6-O*-sulfation and a concomitant decrease in CS *4-O-*sulfation on drug treatments observed in both brain regions, indicating a probable enzymatic aberration in sulfotransferase or sulfatase enzymes leading to biosynthetic or degradation pathways for CS *4-O* and *6-O* sulfation.

To explore this, we overexpressed ARSB (A) or downregulated it by shRNA (S), an enzyme responsible for the degradation of CS *4-0* sulfation. A significant increase in CS *4-0* levels in the LH by AAV delivery of an shRNA to ARSB was observed (Supplemental Fig. S4). Notably, the downregulation of ARSB decreased anxiety in mice and increased their preference for novelty over cocaine during withdrawal (Fig. 6), suggesting that restoring the levels of CS *4-0* altered by the treatment with cocaine can reduce the severity of withdrawal manifestations. A previous study on astrocytes reported inhibition of ARSB on treatment with ethanol, triggering the increase in *4-0* sulfation and inhibiting astrocyte-mediated neurite outgrowth (37). A similar increase in 4-*0* sulfation was reported in brain injury (38). Notably, *6-0* sulfation has been reported to correlate with axonal inhibition and cortical plasticity (39, 40). Together with the present results, these observations support that CS sulfation patterns likely contribute to drug-induced neural plasticity in brain regions like the LH (14).

Chondroitin sulfate proteoglycans (CSPGs) are spatio-temporarily expressed during brain development and regulate critical processes, such as neuronal migration, neurite growth, axon outgrowth, synaptogenesis, and synaptic maturation (41, 42, 43). Elevated levels of CSPGs are present in brain extracellular matrix structures, including perineuronal nets (PNNs), where they stabilize synaptic connections and regulate neural plasticity (44, 45). PNNs are widely detected in both LH and ST in mice, and they have been associated with drug addiction (46). Intriguingly, PNNs in the LH have an active role in regulating cue-induced reinstatement of cocaine-seeking behavior, and their removal *via* chondroitinase (CHABC) administration abolished the acquisition of cocaine-induced conditioned place preference and attenuated the acquisition of cocaine self-administration (47, 48). Moreover, the removal of CS GAG chains with CHABC enzyme restores plasticity in adult rat visual cortex (49). CSPGs are also known to impede axonal regeneration and treatment with CHABC enhanced axonal growth (50). Together, these studies document the essential roles of CS and their sulfation pattern in axonal guidance and synaptic plasticity, the two key processes associated with the drug of abuse (51, 52, 53, 54).

Our proteomics data revealed a number of aberrant proteins in drug-treated *versus* saline samples, which have been previously reported in psychostimulant brain studies (35, 55, 56, 57, 58, 59, 60), and some unique proteins not reported earlier, including CSPG5, TENR, HSPG, and CALR. Specifically, two CSPGs, NCAN and CSPG5, were increased by both drug treatments in ST. A previous study on ethanol treatment also illustrated an increase in NCAN that inhibited astrocyte-mediated neurite outgrowth (37). In addition, some of the CSPG-interacting proteins, including HDGF and ANXA7, were increased, while TENR and annexin A6 were decreased with drug treatment compared to saline. Upregulation of annexin A7, an annexin family of calcium-dependent phospholipid binding proteins has been previously reported in the ST with cocaine treatment (55). Taken together, these data support that aberration in both GAG and proteoglycan regulation play a role in the pathogenesis of drug abuse.

Several proteins involved in neurodegenerative disorders such as Alzheimer's and Parkinson's disease, including Tau, APO-E, APO-D, A4, and SYUA, were observed to be increased by the treatment with both stimulants in ST for drug-treated relative to saline samples. Several studies have documented the link between drug abuse and neurodegeneration, including a previous study that suggested dysregulation of iron homeostasis in cocaine-addicted brains that leads to cell death, a phenomenon also observed in neurodegeneration (61). Intriguingly, proteoglycans, through their GAG chains, are known to play essential roles in the aggregation of these proteins in neurodegenerative disorders (62, 63, 64). Thus, there could be an association between aberrant GAGs and neurodegenerative proteins observed in our study. The role of drug-induced neurodegeneration, suggested by the present results, is a finding that warrants further exploration.

In conclusion, little is known about aberration in HS, CS, and proteins in the LH and ST with drug abuse. Here, we highlighted unique alterations in specific HS and CS disaccharides and some key proteins after cocaine and METH exposure which may serve as novel markers and therapeutic targets for substance use disorder. In addition, the altered pathways and associated molecular mechanisms, as reported here, will strengthen our fundamental knowledge of the neurobiology of addiction. The expression patterns reported by this study and the functional roles discussed herein need to be further characterized to unravel the biological implication of drug abuse in the brain.

### Analytical Considerations and Future Directions

Due to highly complex GAG structures, proteoglycans (PGs) are not identified effectively with MS and require further fractionation and removal of GAG structures to achieve deep sequencing of PGs (65). Thus, it is unsurprising that we do not observe many PGs in the proteomics data and only observe two differentially expressed CSPGs in the ST. At the same time, a number of GAG disaccharides are differentially expressed at the glycomics level. Altered peptides are generally present at a very low level and require further processing and/or targeted experiments. We, therefore, in the future, aim to achieve in-depth profiles of proteoglycans by immunoprecipitating CS-PG and HS-PGs, removing high molecular weight GAG chains to minimize complexity and further analyzing them by MS to determine the altered expression of PGs in the drug abuse.

## Data Availability

The datasets generated and/or analyzed during the current study are available in the ProteomeXchange Consortium *via* the PRIDE (66) partner repository with the dataset identifier PXD038576 and 10.6019/PXD038576.

Reviewer account details are:

Username: reviewer_pxd038576@ebi.ac.uk

Password: 8Z0BFNBp

## Supplemental data

This article contains supplemental data (4, 5, 6, 7, 8).

## Conflict of interest

The authors declare no competing interests.
