# Supplementary material for: Comprehensive Glycomic and Proteomic Analysis of Mouse Striatum and Lateral Hypothalamus Following Repeated Exposures to Cocaine or Methamphetamine
Source: Mol Cell Proteomics. 2024 Jun 15;23(8):100803. doi: 10.1016/j.mcpro.2024.100803 (PMC11324981; doi:10.1016/j.mcpro.2024.100803)
Supplement: Supplemental PDF S1 [file mmc11.pdf]

Total Ion Chromatograms (TICs)

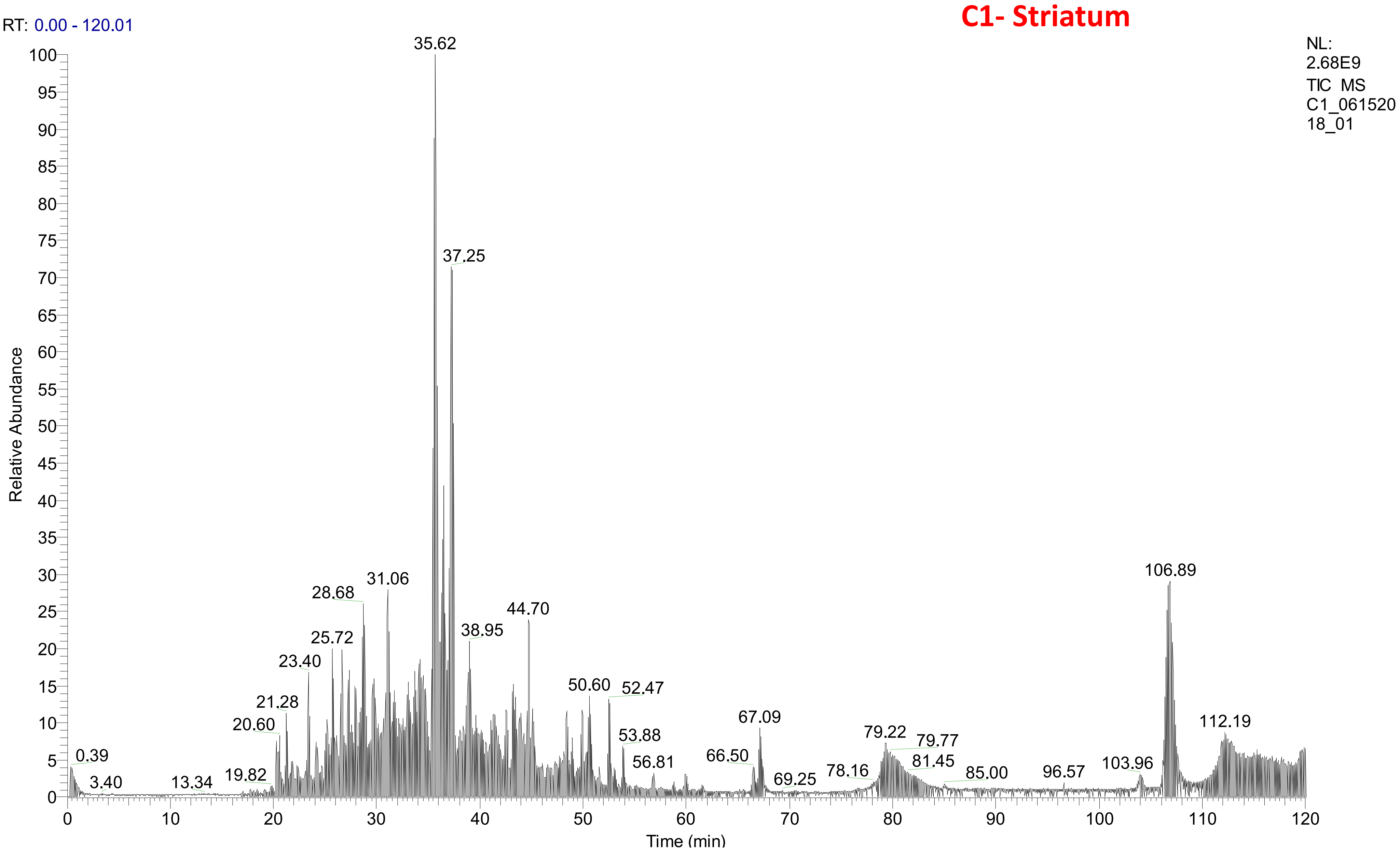
C1- Striatum
RT: 0.00 - 120.01
NL:
2.68E9
TIC MS
C1_061520
18_01
Relative Abundance
Time (min)
35.62
37.25
31.06
28.68
44.70
38.95
25.72
23.40
21.28
20.60
0.39
3.40
13.34
19.82
50.60
52.47
53.88
56.81
66.50
67.09
69.25
78.16
79.22
79.77
81.45
85.00
96.57
103.96
106.89
112.19

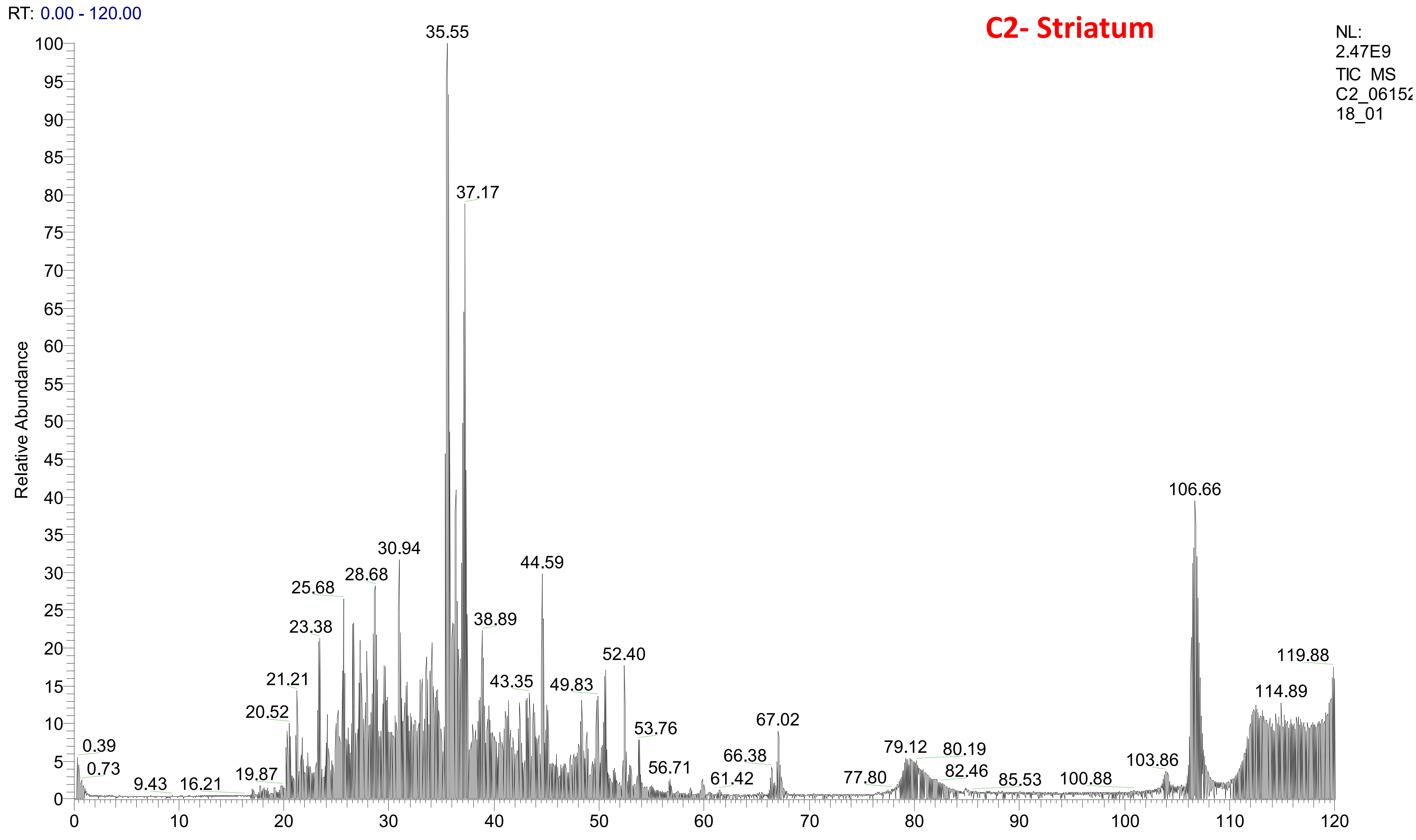

RT: 0.00 - 120.00
C2- Striatum
NL: 2.47E9
TIC MS
C2_06152
18_01
Relative Abundance
35.55
37.17
30.94
44.59
28.68
25.68
23.38
38.89
52.40
21.21
43.35
49.83
20.52
53.76
67.02
0.39
0.73
9.43
16.21
19.87
56.71
61.42
66.38
77.80
79.12
80.19
82.46
85.53
100.88
103.86
106.66
114.89
119.88

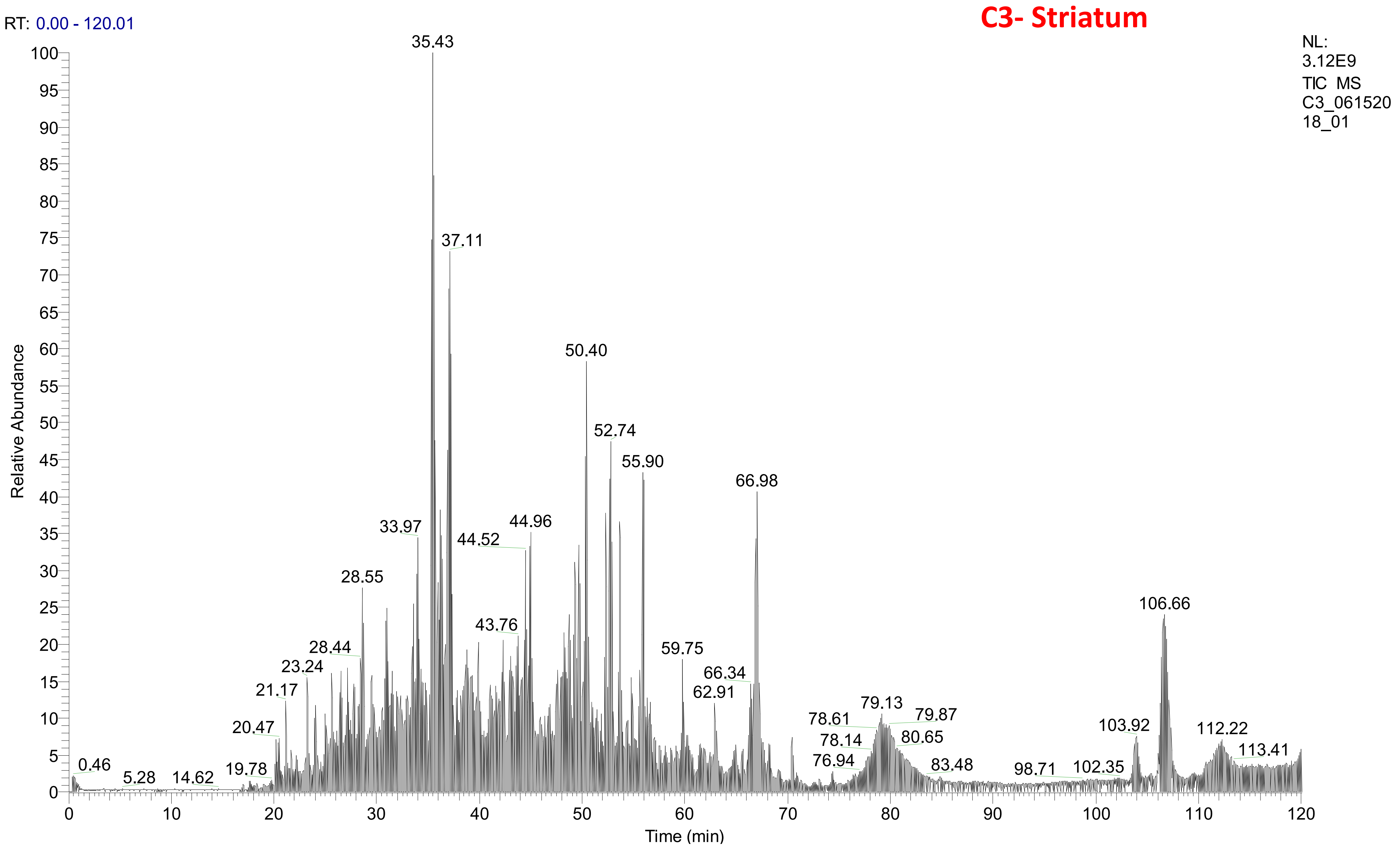

C3- Striatum
RT: 0.00 - 120.01
NL:
3.12E9
TIC MS
C3_061520
18_01
Relative Abundance
Time (min)
35.43
37.11
50.40
52.74
55.90
66.98
33.97
44.96
44.52
28.55
43.76
59.75
28.44
23.24
66.34
21.17
62.91
20.47
79.13
79.87
78.61
80.65
78.14
76.94
83.48
103.92
106.66
112.22
113.41
98.71
102.35
0.46
5.28
14.62
19.78

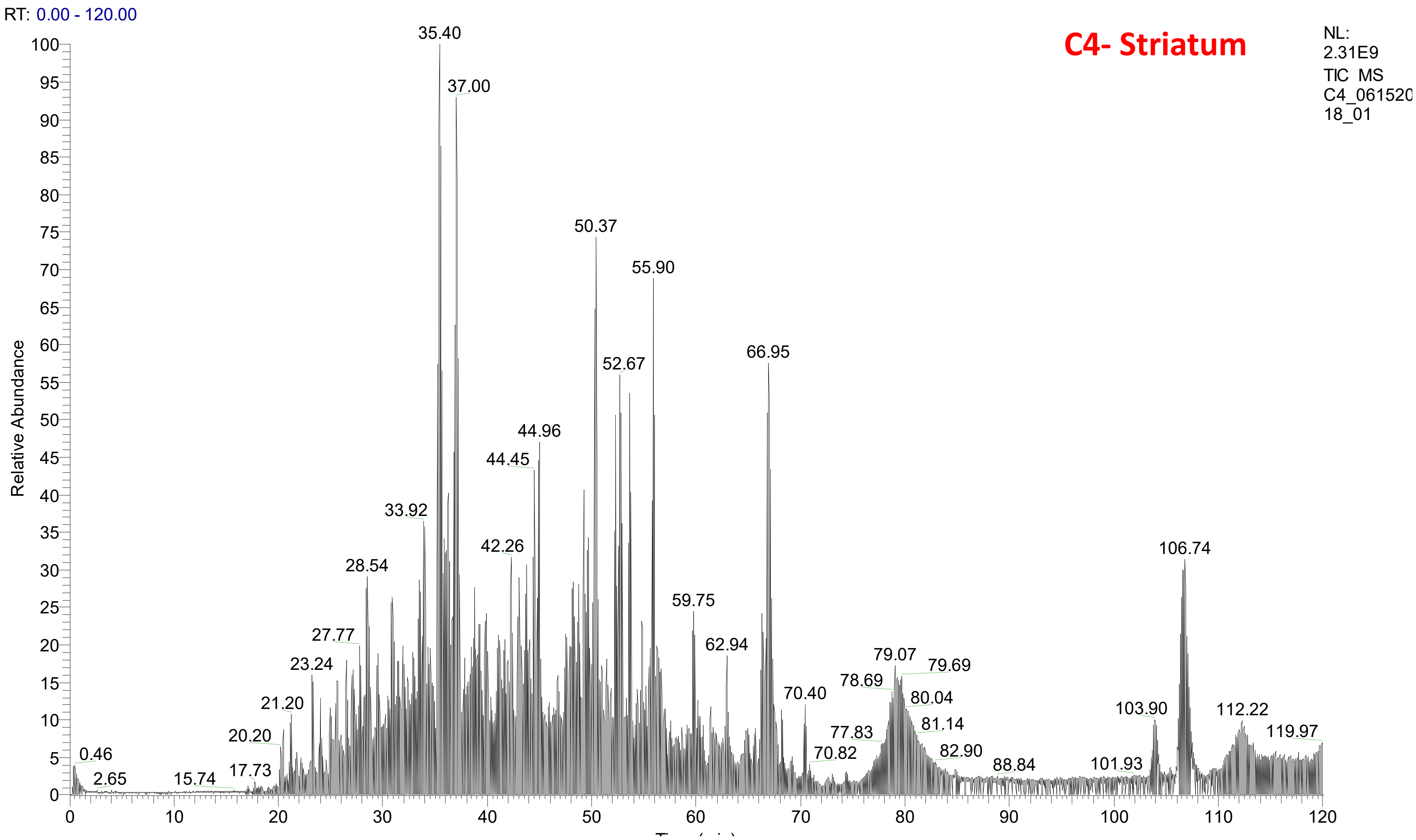
RT: 0.00 - 120.00
C4- Striatum
NL:
2.31E9
TIC MS
C4_061520
18_01
Relative Abundance
35.40
37.00
50.37
55.90
66.95
52.67
44.96
44.45
33.92
42.26
28.54
106.74
59.75
62.94
27.77
23.24
79.07
79.69
78.69
80.04
21.20
70.40
103.90
112.22
81.14
20.20
77.83
119.97
0.46
70.82
82.90
17.73
88.84
101.93
2.65
15.74

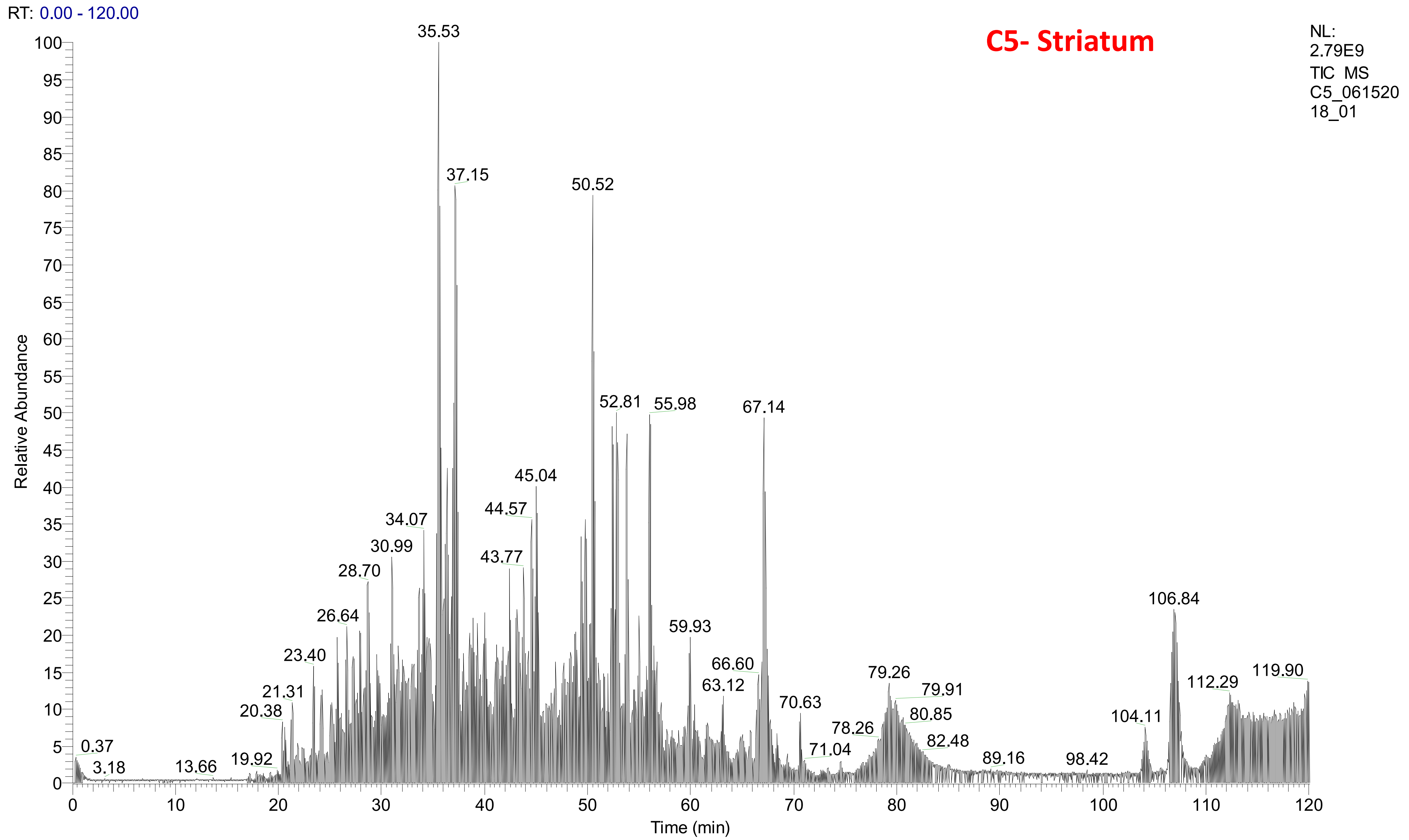
RT: 0.00 - 120.00
C5- Striatum
NL:
2.79E9
TIC MS
C5_061520
18_01
Relative Abundance
Time (min)
35.53
37.15
50.52
52.81
55.98
67.14
45.04
44.57
34.07
30.99
43.77
28.70
26.64
23.40
21.31
20.38
19.92
13.66
3.18
0.37
59.93
66.60
63.12
70.63
71.04
78.26
79.26
79.91
80.85
82.48
89.16
98.42
104.11
106.84
112.29
119.90

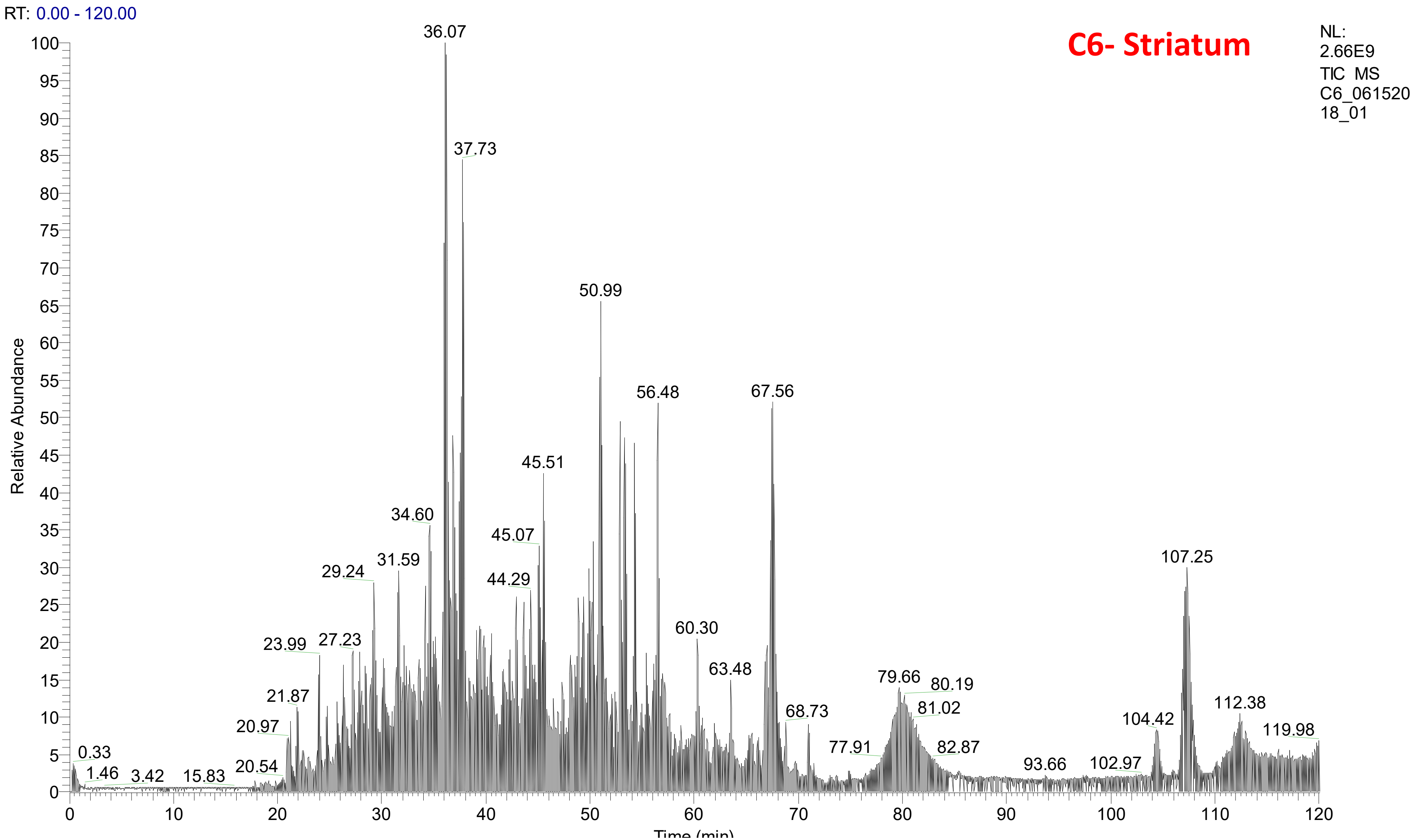
RT: 0.00 - 120.00
C6- Striatum
NL:
2.66E9
TIC MS
C6_061520
18_01
Relative Abundance
Time (min)
36.07
37.73
50.99
56.48
67.56
45.51
34.60
45.07
31.59
29.24
44.29
107.25
60.30
23.99
27.23
63.48
79.66
80.19
21.87
112.38
81.02
68.73
104.42
20.97
119.98
0.33
77.91
82.87
1.46
3.42
15.83
20.54
93.66
102.97

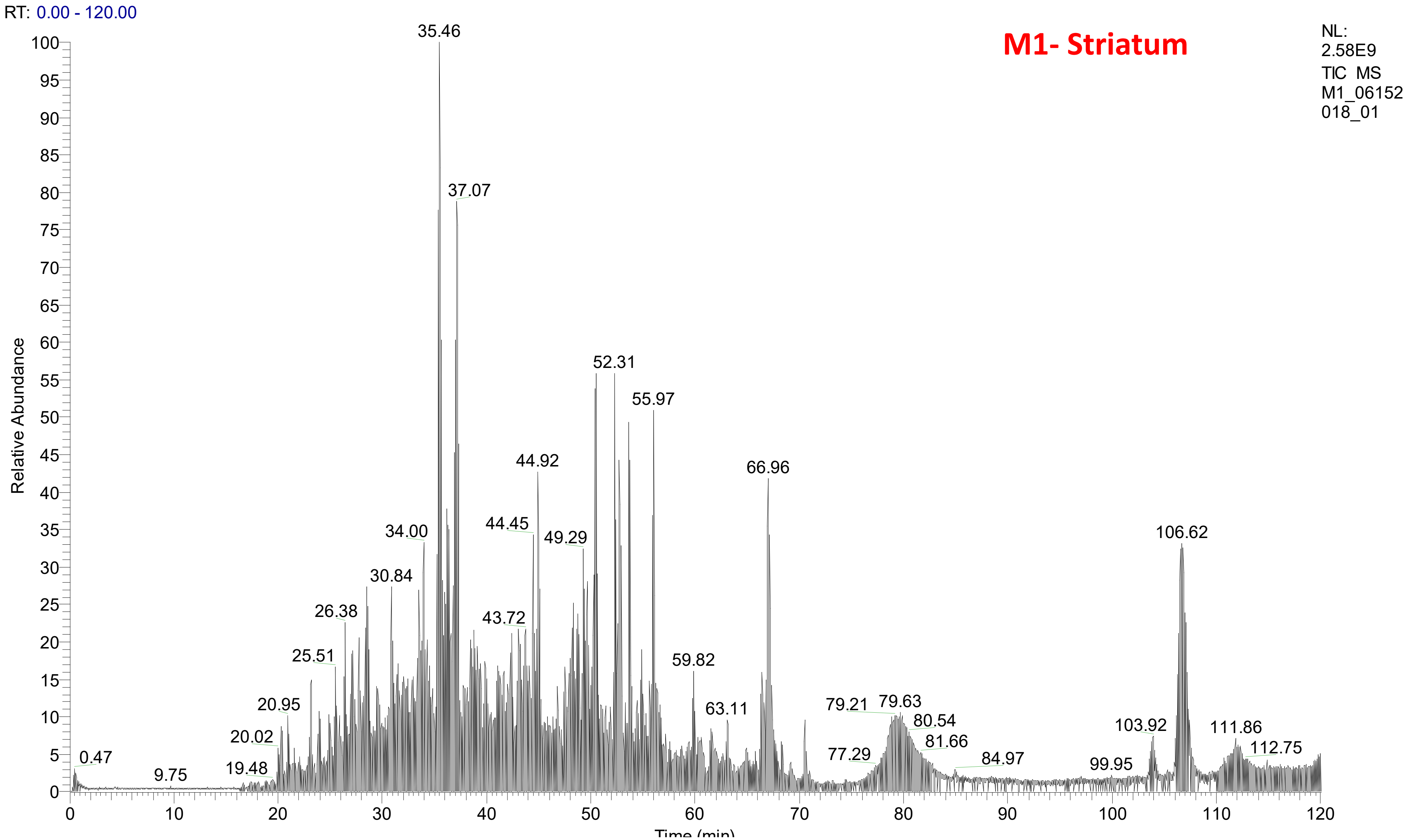
RT: 0.00 - 120.00
M1- Striatum
NL: 2.58E9
TIC MS
M1_06152 018_01
Relative Abundance
Time (min)
35.46
37.07
52.31
55.97
44.92
66.96
44.45
49.29
34.00
106.62
30.84
26.38
43.72
25.51
59.82
20.95
63.11
79.21
79.63
80.54
81.66
20.02
103.92
111.86
112.75
0.47
77.29
84.97
99.95
19.48
9.75

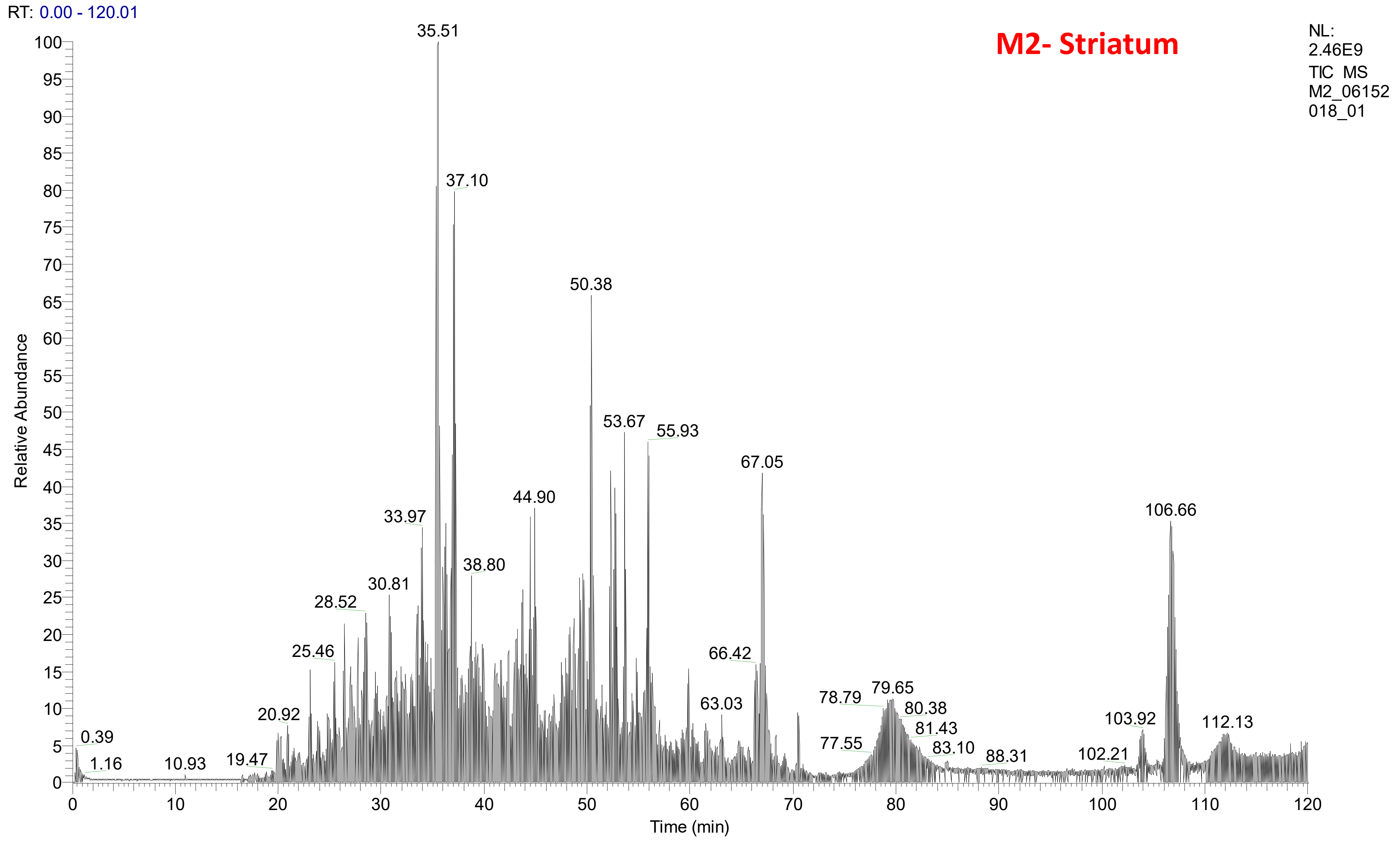
RT: 0.00 - 120.01
M2- Striatum
NL:
2.46E9
TIC MS
M2_06152
018_01
Relative Abundance
Time (min)
35.51
37.10
50.38
53.67
55.93
67.05
44.90
33.97
106.66
38.80
30.81
28.52
25.46
66.42
79.65
78.79
80.38
63.03
20.92
103.92
112.13
81.43
0.39
77.55
83.10
88.31
102.21
19.47
1.16
10.93

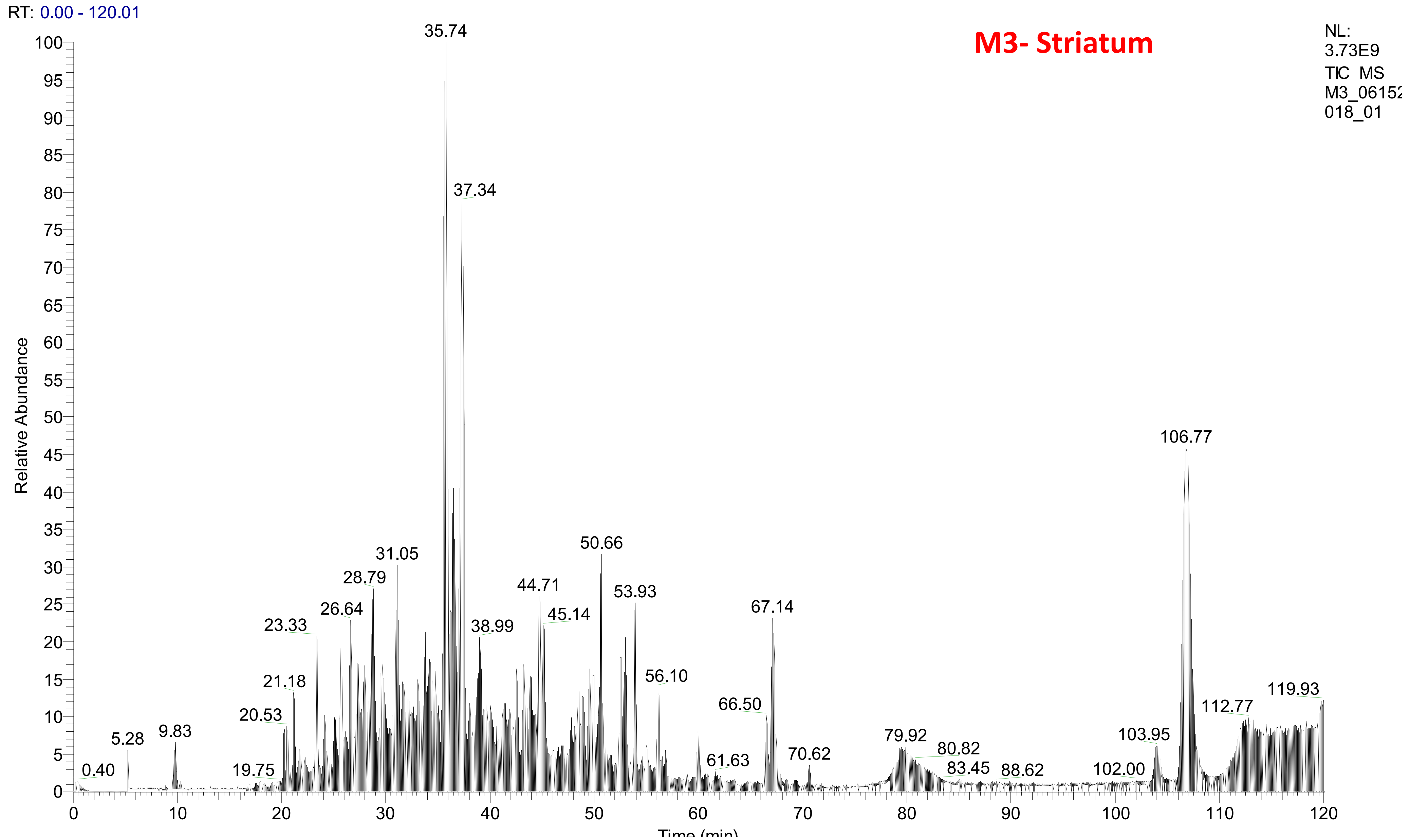
RT: 0.00 - 120.01
M3- Striatum
NL:
3.73E9
TIC MS
M3_06152
018_01
Relative Abundance
Time (min)
35.74
37.34
106.77
31.05
50.66
28.79
44.71
53.93
26.64
67.14
45.14
23.33
38.99
21.18
56.10
119.93
20.53
66.50
112.77
9.83
5.28
79.92
103.95
80.82
70.62
61.63
83.45
0.40
19.75
88.62
102.00

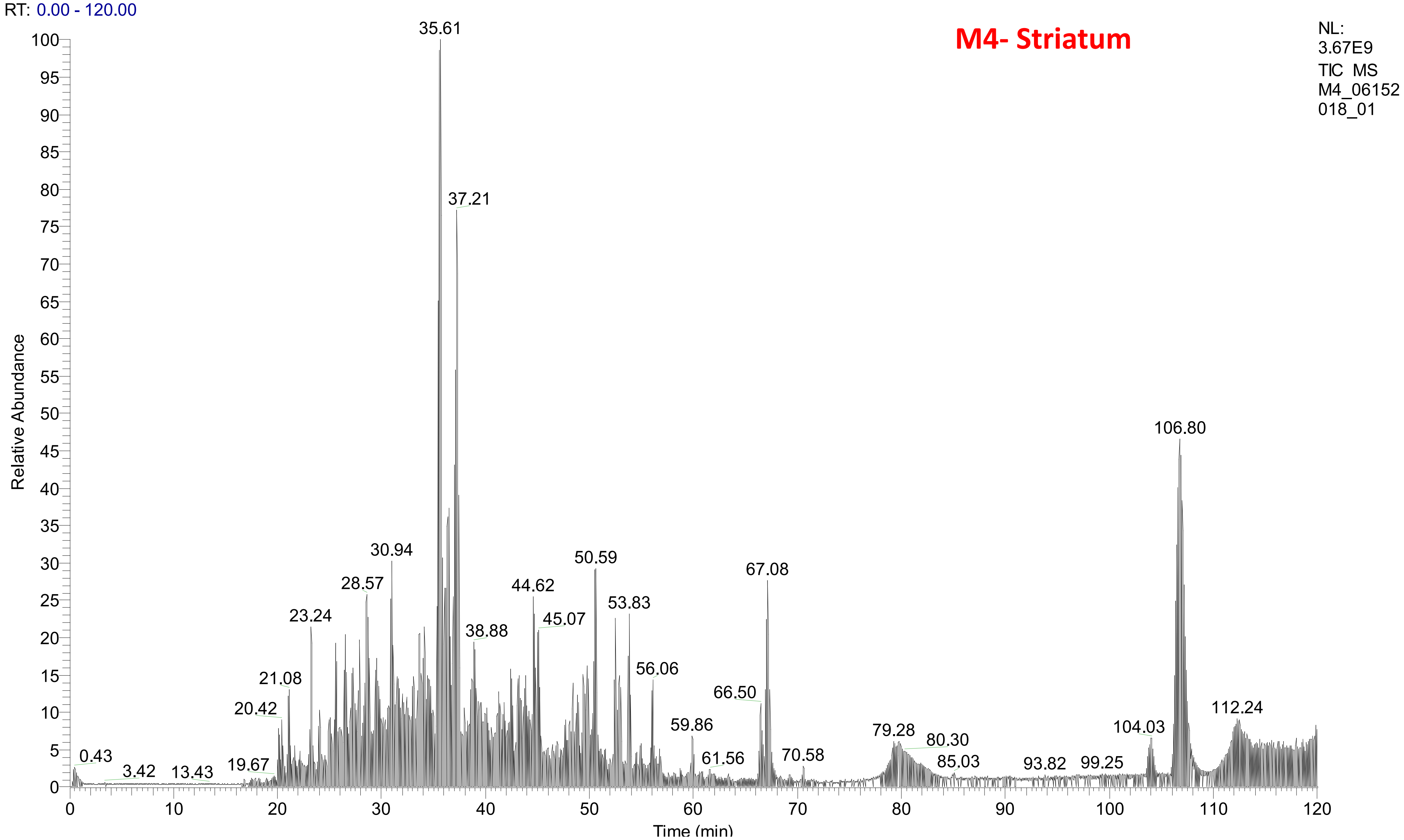
RT: 0.00 - 120.00
M4- Striatum
NL:
3.67E9
TIC MS
M4_06152
018_01
Relative Abundance
Time (min)
35.61
37.21
106.80
30.94
50.59
67.08
28.57
44.62
53.83
23.24
45.07
38.88
21.08
56.06
66.50
20.42
112.24
59.86
104.03
79.28
80.30
0.43
61.56
70.58
85.03
93.82
99.25
3.42
13.43
19.67

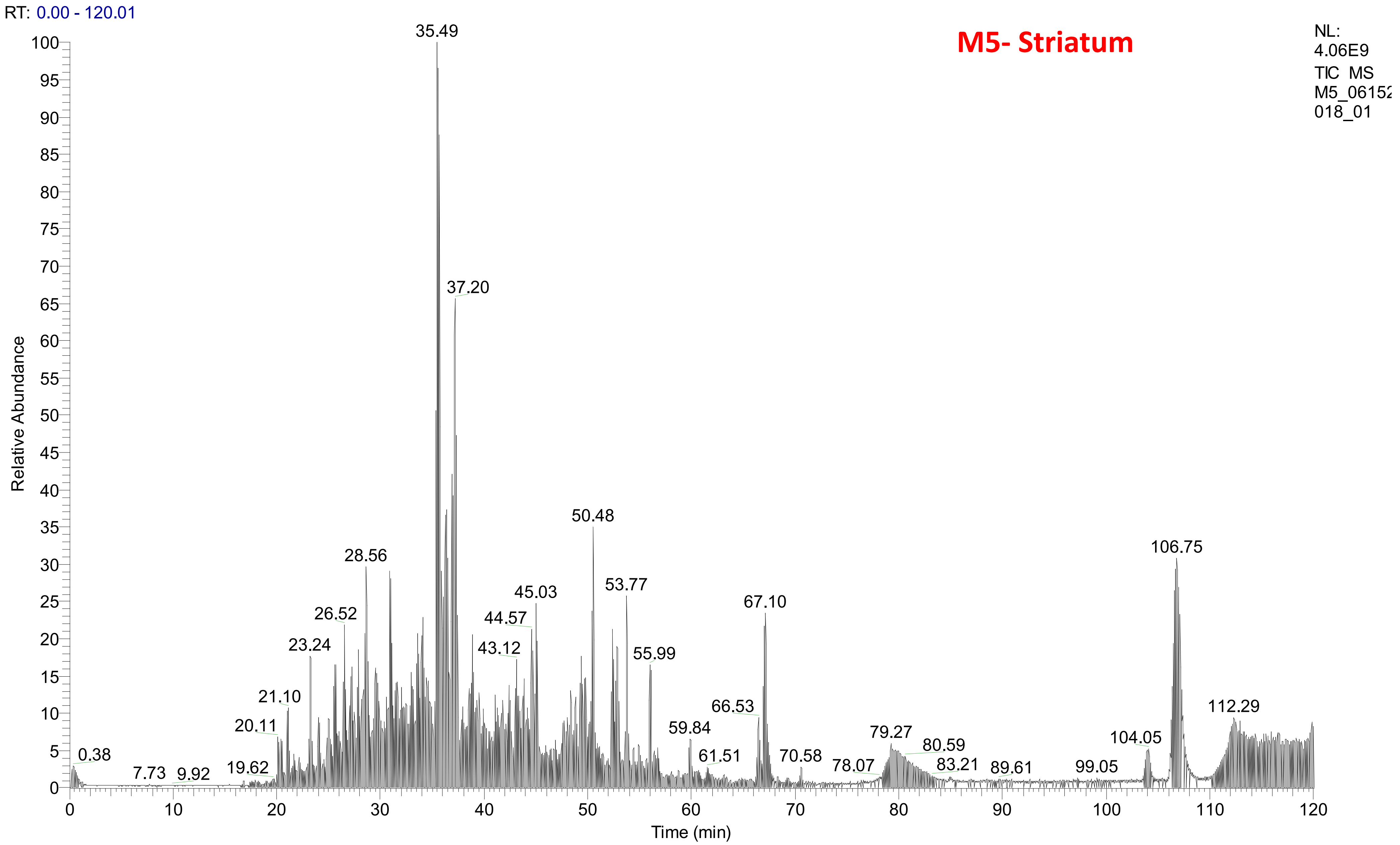
RT: 0.00 - 120.01
M5- Striatum
NL:
4.06E9
TIC MS
M5_06152
018_01
35.49
37.20
50.48
28.56
106.75
53.77
45.03
67.10
26.52
44.57
23.24
43.12
55.99
21.10
112.29
66.53
20.11
59.84
79.27
104.05
80.59
0.38
61.51
70.58
83.21
78.07
89.61
99.05
7.73
9.92
19.62
Relative Abundance
Time (min)

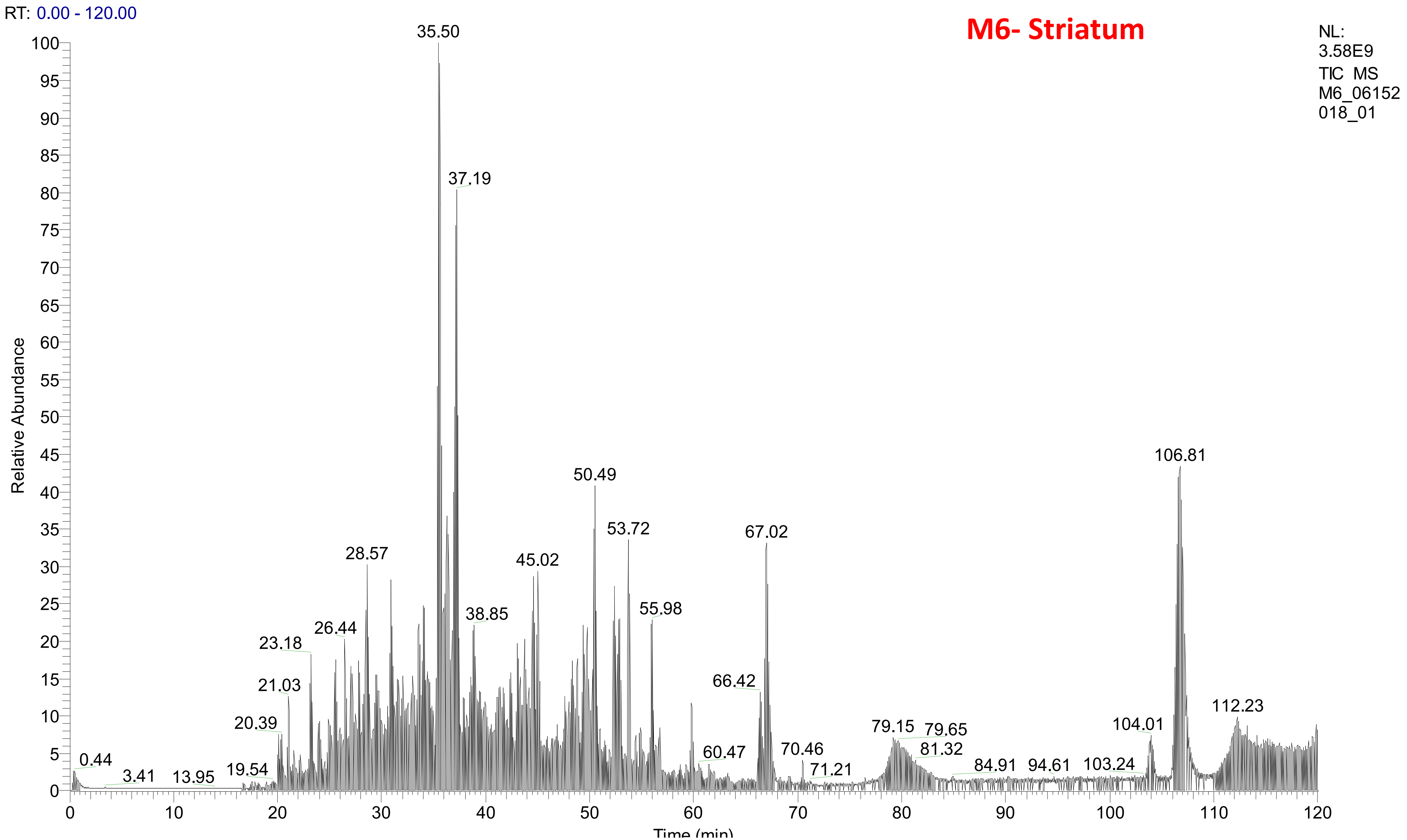
RT: 0.00 - 120.00
M6- Striatum
NL:
3.58E9
TIC MS
M6_06152
018_01
Relative Abundance
Time (min)
35.50
37.19
106.81
50.49
53.72
67.02
28.57
45.02
38.85
55.98
26.44
23.18
21.03
66.42
20.39
112.23
104.01
79.15
79.65
70.46
81.32
60.47
0.44
3.41
13.95
19.54
71.21
84.91
94.61
103.24

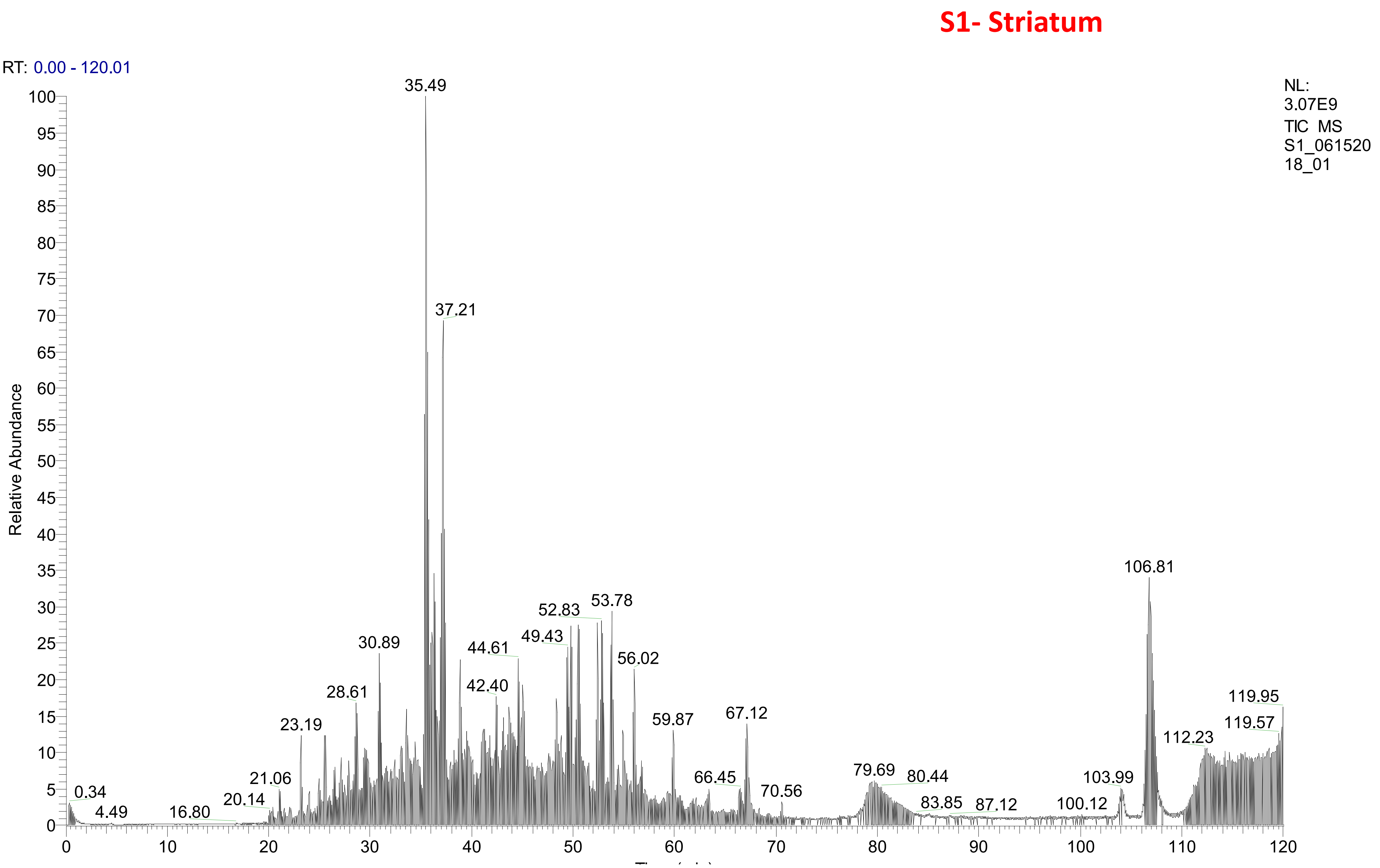
S1- Striatum
RT: 0.00 - 120.01
NL:
3.07E9
TIC MS
S1_061520
18_01
Relative Abundance
35.49
37.21
30.89
28.61
23.19
21.06
20.14
16.80
4.49
0.34
44.61
42.40
49.43
52.83
53.78
56.02
59.87
67.12
66.45
70.56
79.69
80.44
83.85
87.12
100.12
103.99
106.81
112.23
119.57
119.95

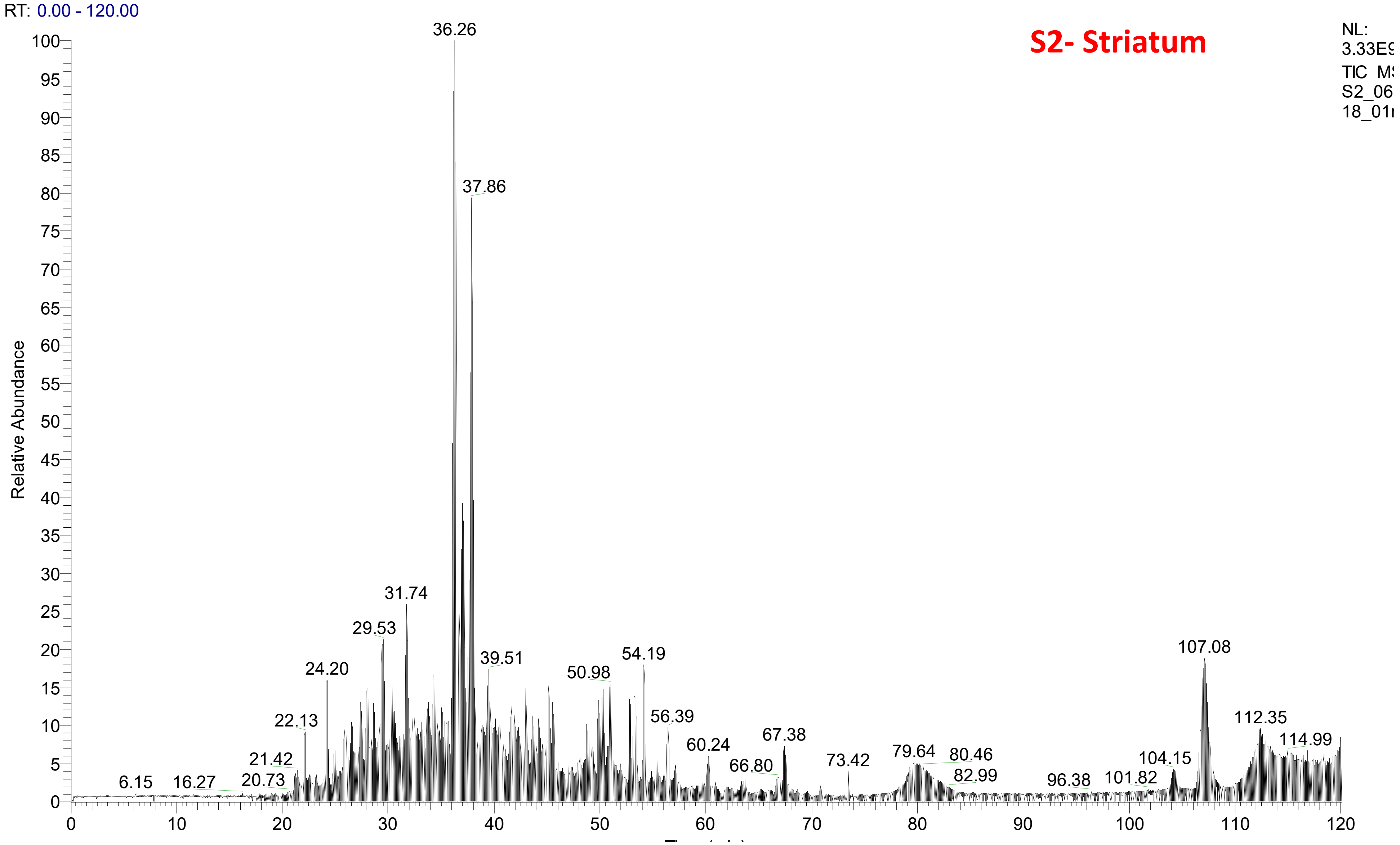

RT: 0.00 - 120.00
S2- Striatum
NL:
3.33E9
TIC MS
S2_06
18_01
Relative Abundance
36.26
37.86
31.74
29.53
39.51
24.20
50.98
54.19
107.08
22.13
56.39
112.35
21.42
60.24
67.38
114.99
66.80
73.42
79.64
80.46
104.15
6.15
16.27
20.73
82.99
96.38
101.82

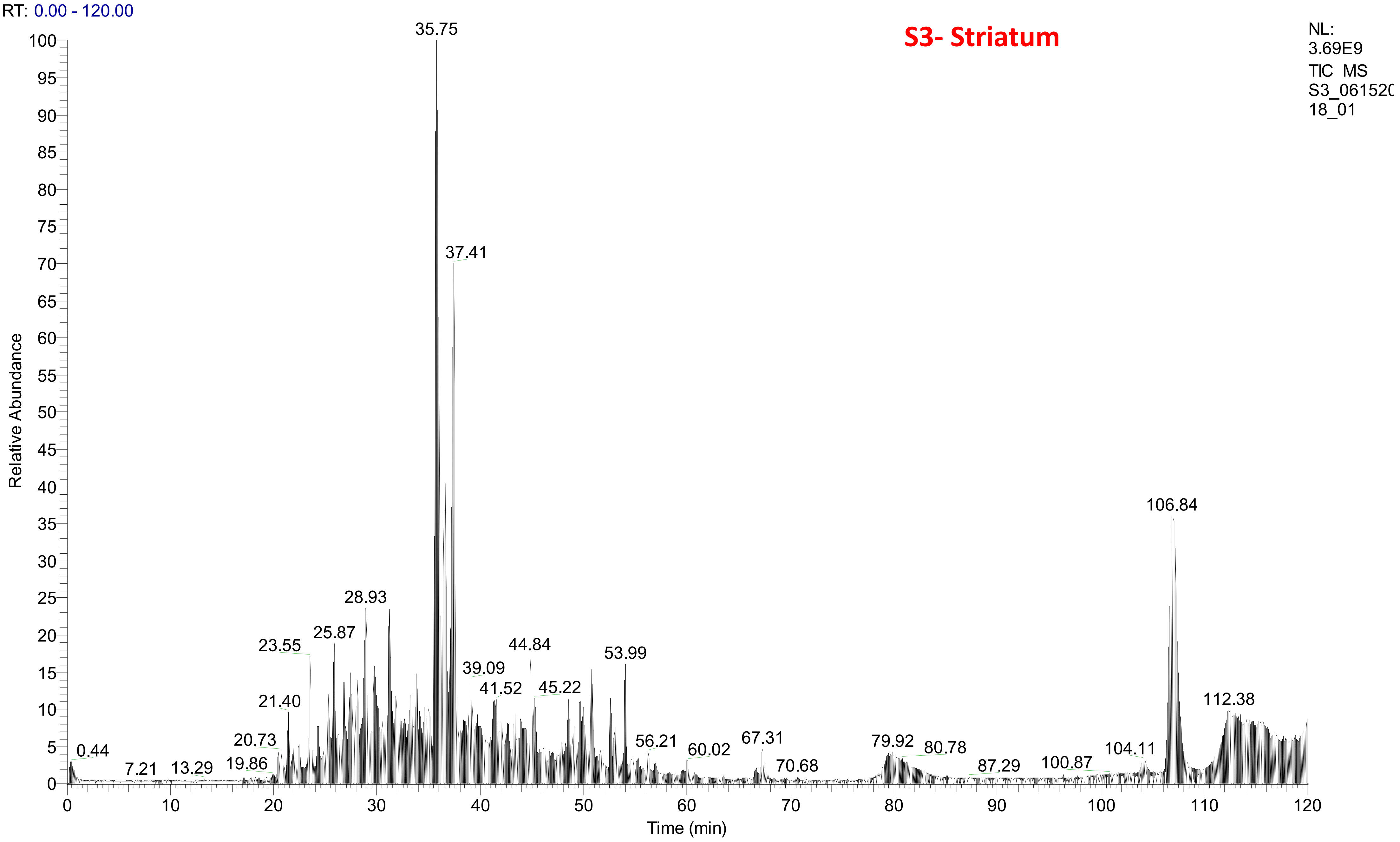
RT: 0.00 - 120.00
S3- Striatum
NL:
3.69E9
TIC MS
S3_061520
18_01
Relative Abundance
Time (min)
35.75
37.41
28.93
25.87
23.55
21.40
20.73
19.86
13.29
7.21
0.44
39.09
41.52
44.84
45.22
53.99
56.21
60.02
67.31
70.68
79.92
80.78
87.29
100.87
104.11
106.84
112.38

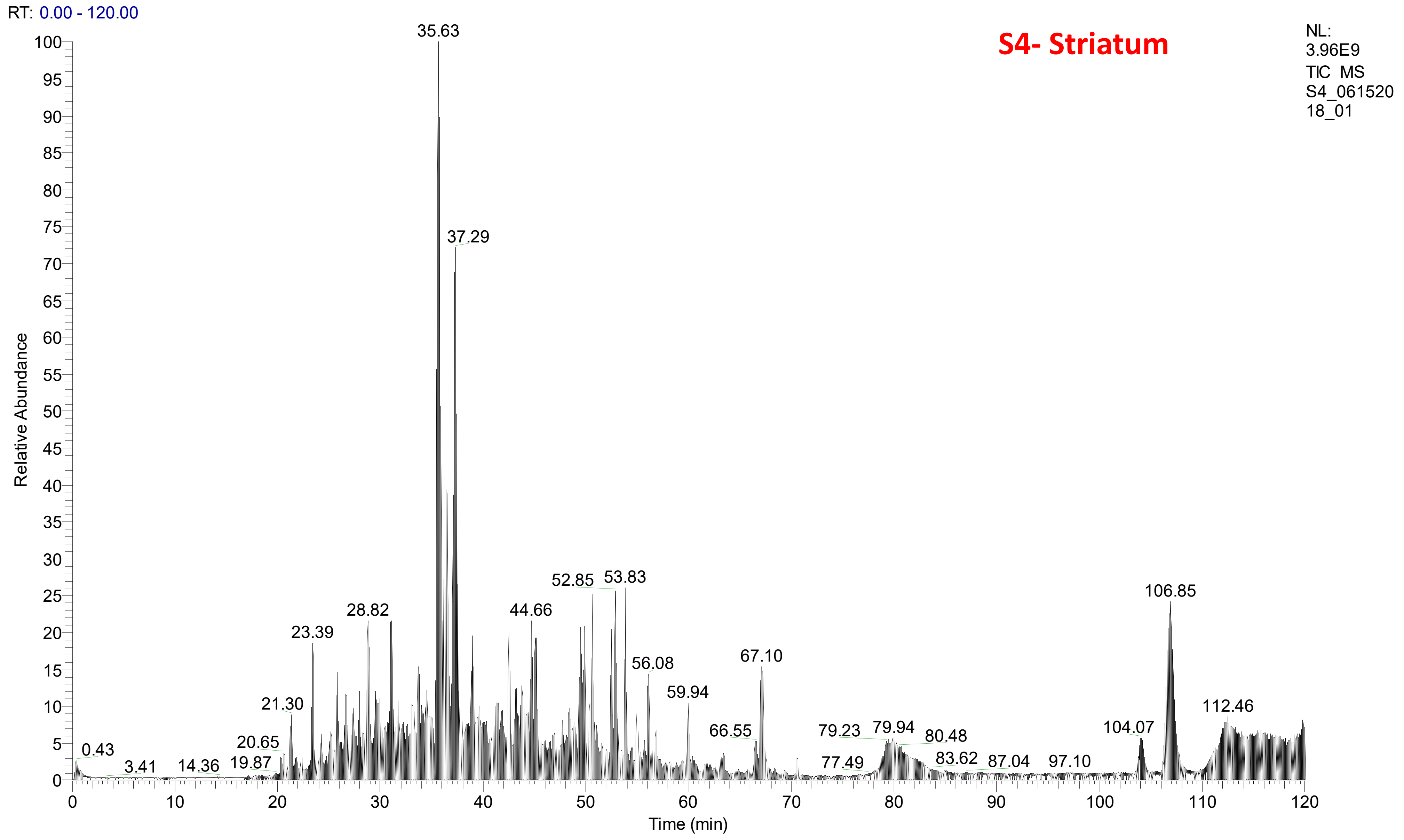

RT: 0.00 - 120.00
S4- Striatum
NL:
3.96E9
TIC MS
S4_061520
18_01
Relative Abundance
Time (min)
35.63
37.29
52.85
53.83
106.85
28.82
44.66
23.39
67.10
56.08
59.94
21.30
112.46
0.43
3.41
14.36
19.87
20.65
66.55
79.23
79.94
80.48
77.49
83.62
87.04
97.10
104.07

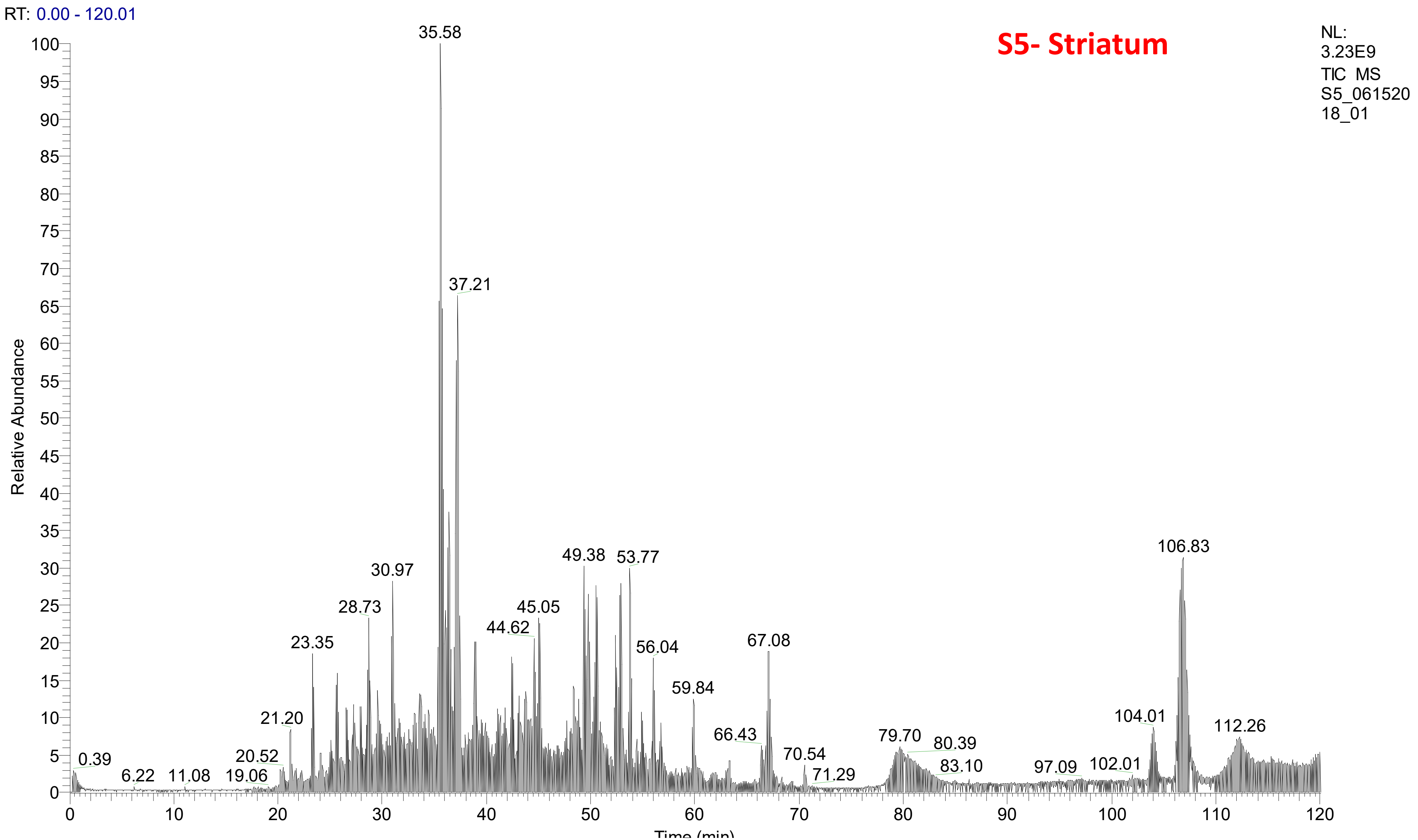

RT: 0.00 - 120.01
S5- Striatum
NL:
3.23E9
TIC MS
S5_061520
18_01
Relative Abundance
Time (min)
35.58
37.21
30.97
28.73
23.35
21.20
20.52
19.06
11.08
6.22
0.39
44.62
45.05
49.38
53.77
56.04
59.84
66.43
67.08
70.54
71.29
79.70
80.39
83.10
97.09
102.01
104.01
106.83
112.26

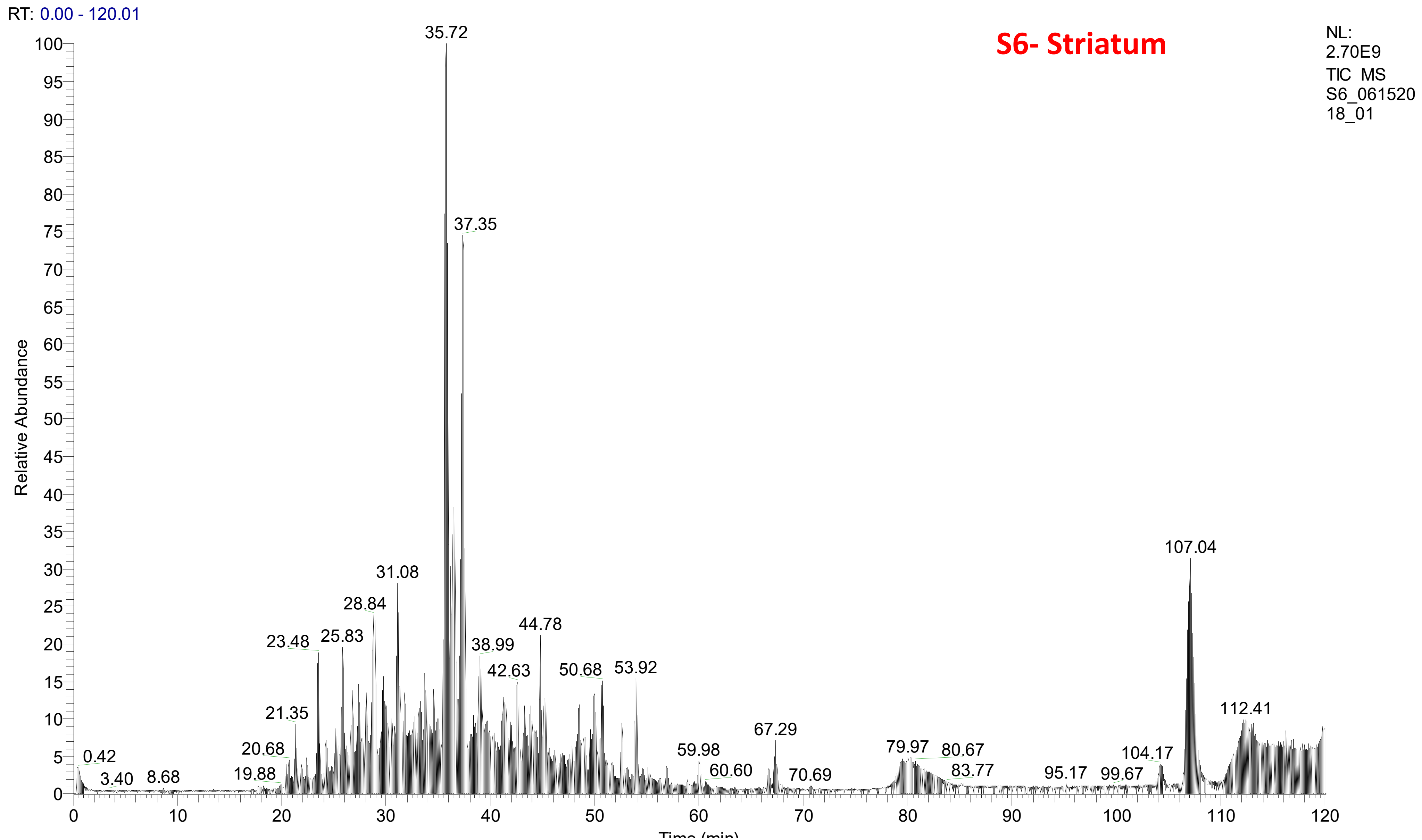
RT: 0.00 - 120.01
S6- Striatum
NL:
2.70E9
TIC MS
S6_061520
18_01
Relative Abundance
35.72
37.35
31.08
28.84
23.48
25.83
44.78
38.99
42.63
50.68
53.92
21.35
20.68
19.88
0.42
3.40
8.68
59.98
60.60
67.29
70.69
79.97
80.67
83.77
95.17
99.67
104.17
107.04
112.41

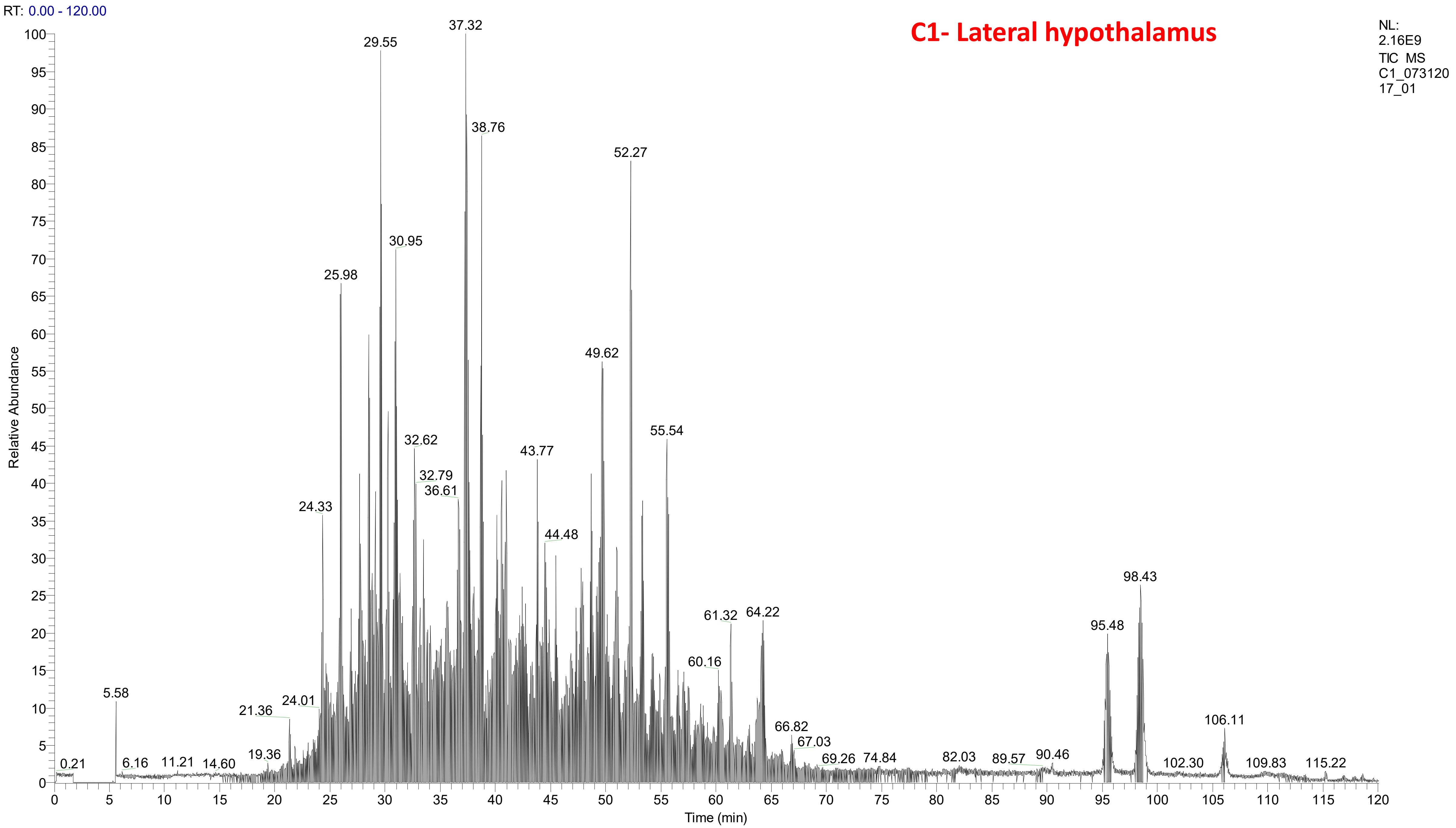
RT: 0.00 - 120.00
C1- Lateral hypothalamus
NL:
2.16E9
TIC MS
C1_073120
17_01
Relative Abundance
Time (min)
37.32
29.55
38.76
52.27
30.95
25.98
49.62
55.54
43.77
32.62
32.79
36.61
24.33
44.48
98.43
61.32
64.22
95.48
60.16
5.58
24.01
21.36
106.11
66.82
67.03
19.36
69.26
74.84
82.03
89.57
90.46
0.21
6.16
11.21
14.60
102.30
109.83
115.22

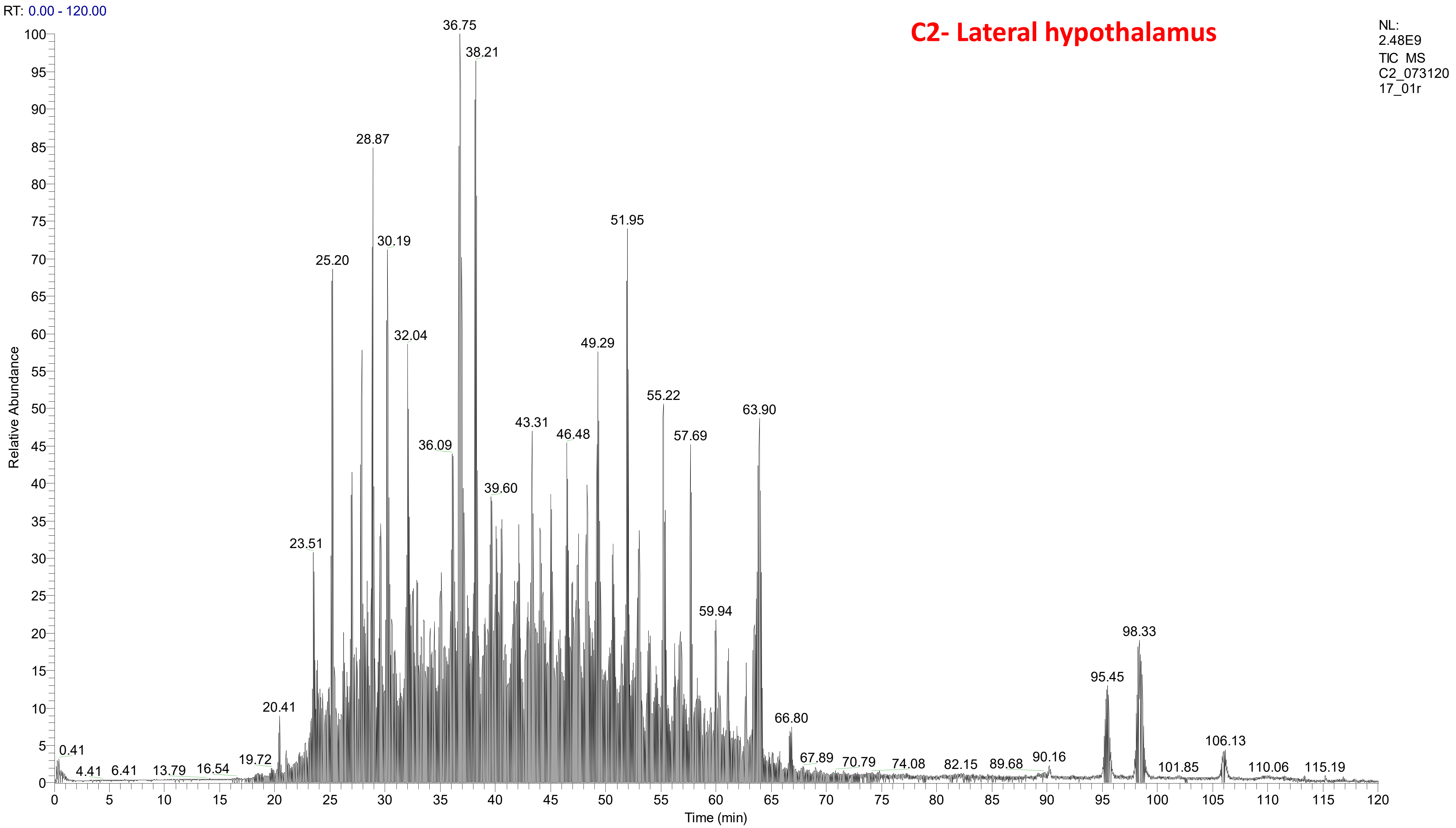

RT: 0.00 - 120.00
C2- Lateral hypothalamus
NL:
2.48E9
TIC MS
C2_073120
17_01r
Relative Abundance
Time (min)
36.75
38.21
28.87
51.95
30.19
25.20
32.04
49.29
55.22
63.90
43.31
46.48
57.69
36.09
39.60
23.51
59.94
98.33
95.45
20.41
66.80
106.13
0.41
4.41
6.41
13.79
16.54
19.72
67.89
70.79
74.08
82.15
89.68
90.16
101.85
110.06
115.19

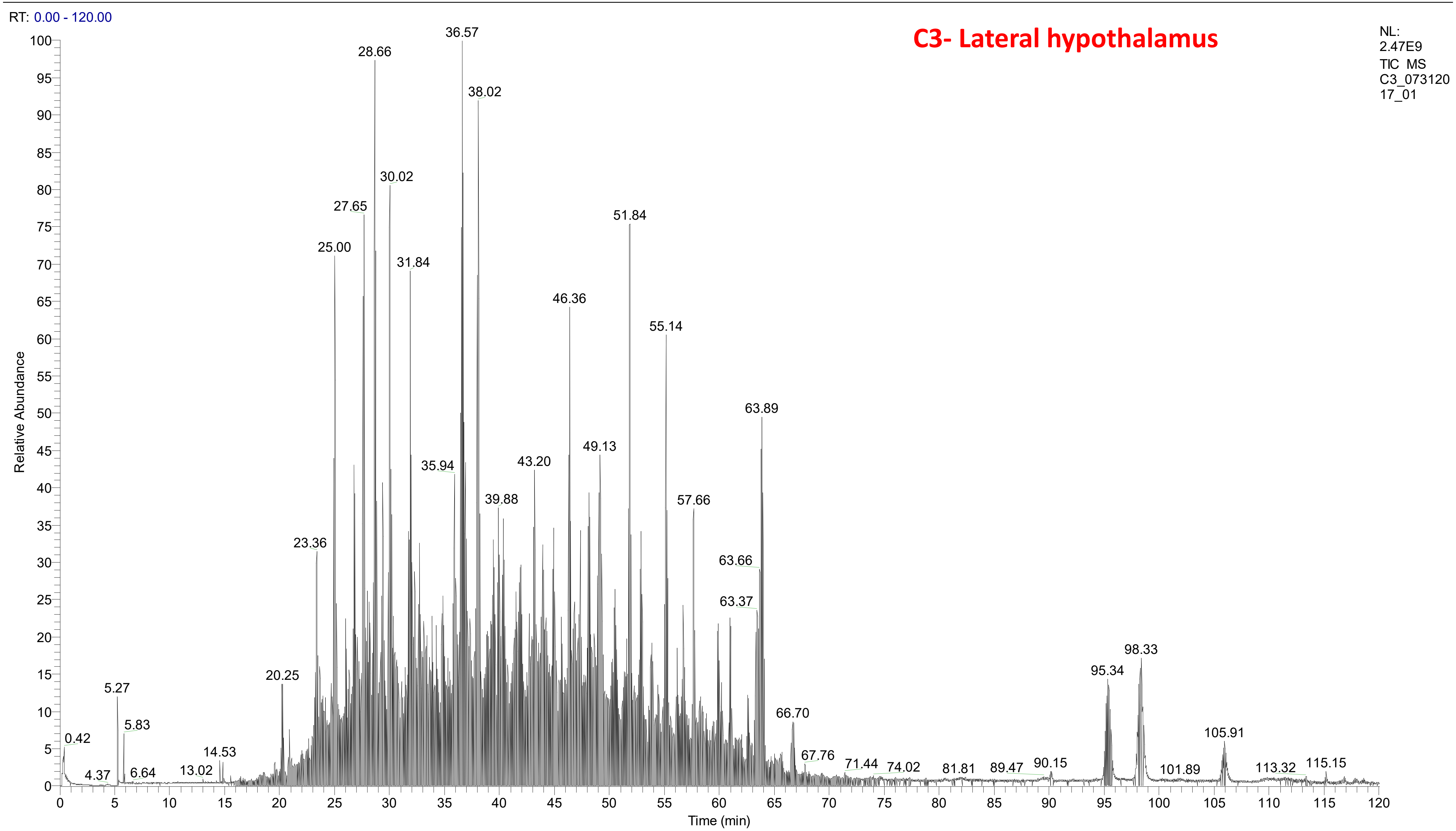
RT: 0.00 - 120.00
C3- Lateral hypothalamus
NL:
2.47E9
TIC MS
C3_073120
17_01
Relative Abundance
Time (min)
36.57
28.66
38.02
30.02
27.65
51.84
25.00
31.84
46.36
55.14
63.89
49.13
43.20
35.94
39.88
57.66
23.36
63.66
63.37
20.25
5.27
5.83
0.42
14.53
13.02
6.64
4.37
98.33
95.34
66.70
105.91
67.76
71.44
74.02
81.81
89.47
90.15
101.89
113.32
115.15

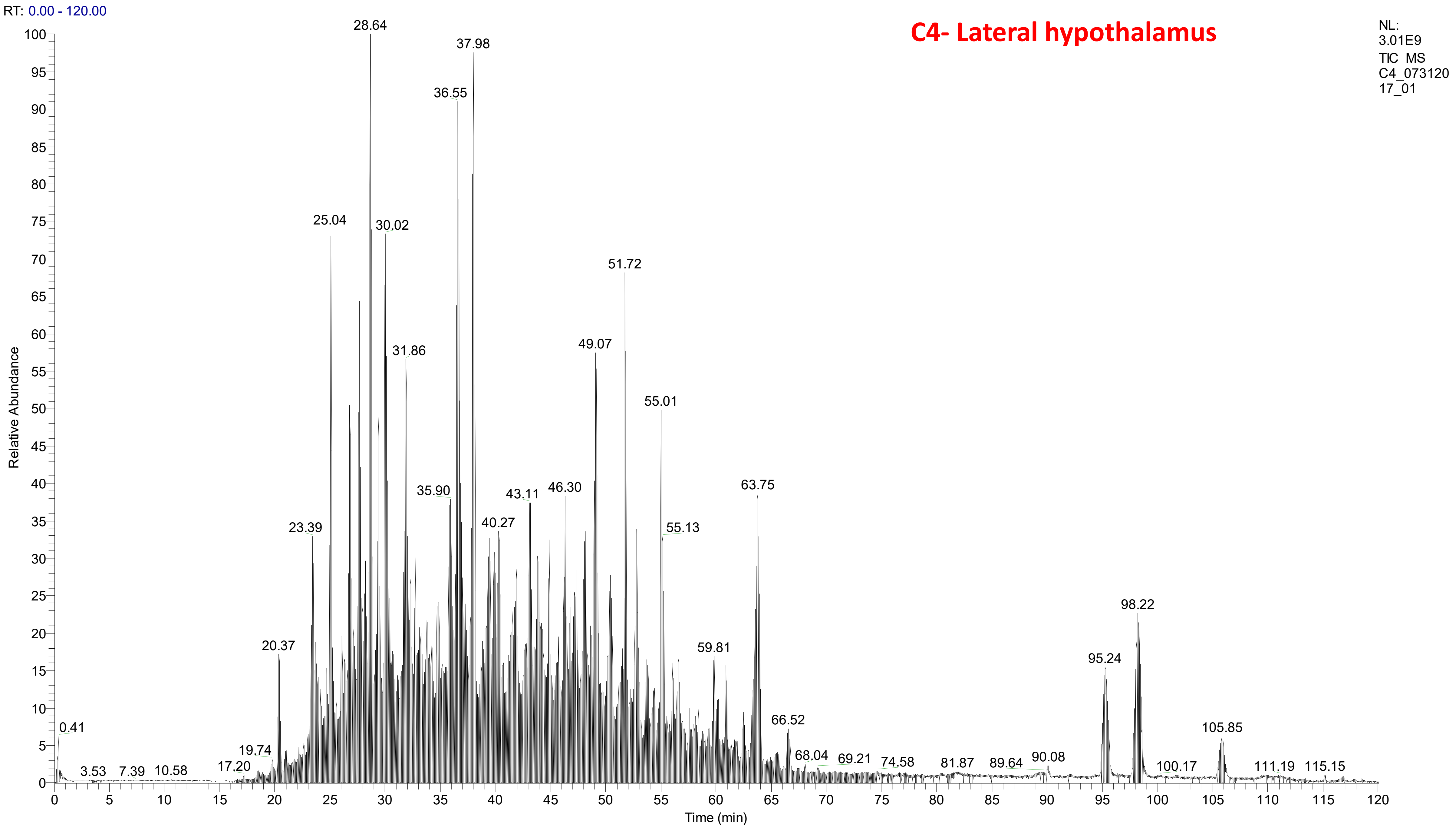

RT: 0.00 - 120.00
C4- Lateral hypothalamus
NL:
3.01E9
TIC MS
C4_073120
17_01
Relative Abundance
Time (min)
28.64
37.98
36.55
25.04
30.02
51.72
31.86
49.07
55.01
35.90
43.11
46.30
63.75
23.39
40.27
55.13
20.37
59.81
98.22
95.24
0.41
66.52
105.85
19.74
17.20
3.53
7.39
10.58
68.04
69.21
74.58
81.87
89.64
90.08
100.17
111.19
115.15

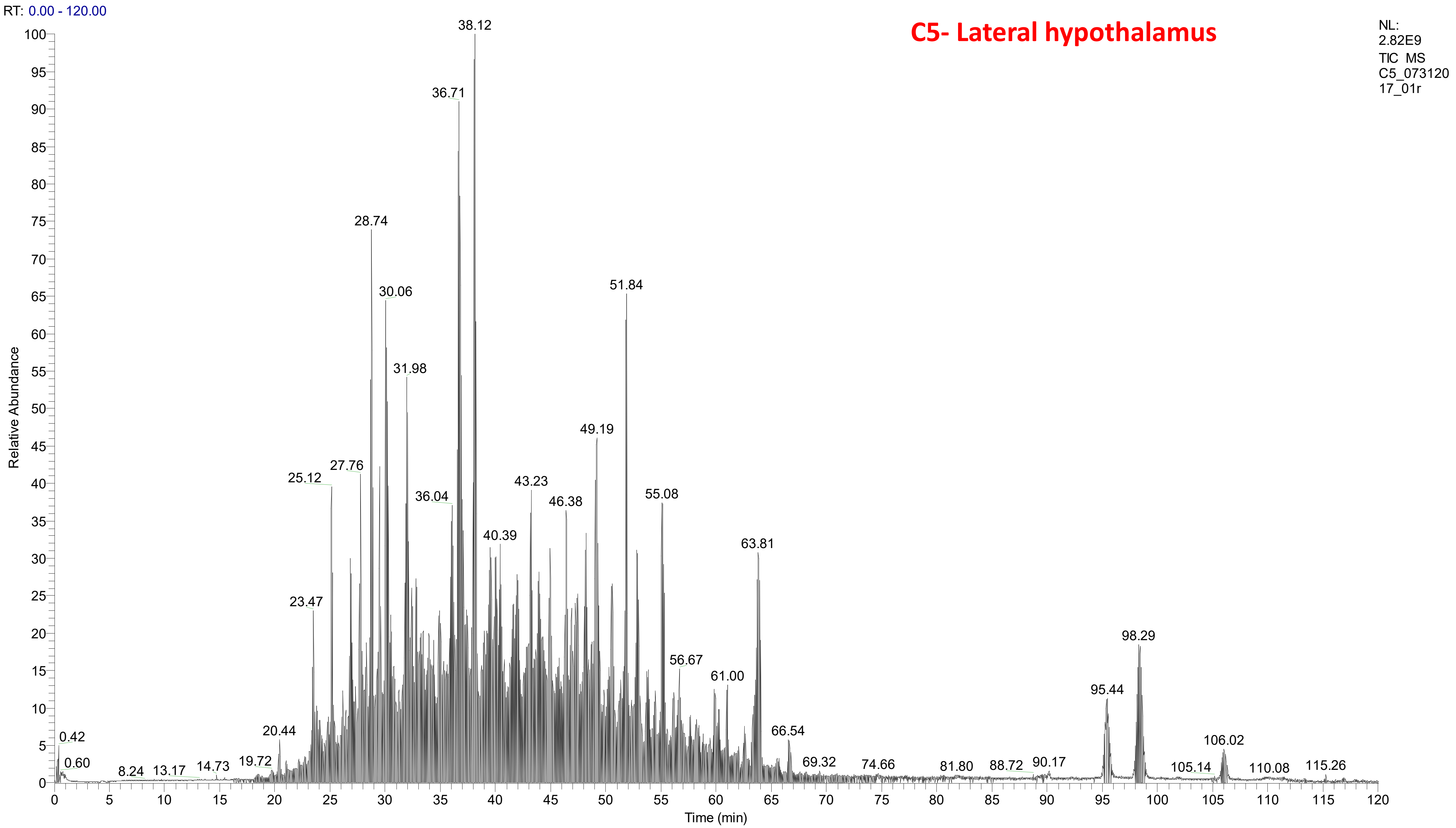
RT: 0.00 - 120.00
C5- Lateral hypothalamus
NL:
2.82E9
TIC MS
C5_073120
17_01r
Relative Abundance
Time (min)
38.12
36.71
28.74
30.06
31.98
51.84
49.19
25.12
27.76
36.04
43.23
46.38
55.08
40.39
63.81
23.47
98.29
56.67
61.00
95.44
0.42
0.60
8.24
13.17
14.73
19.72
20.44
66.54
69.32
74.66
81.80
88.72
90.17
105.14
106.02
110.08
115.26

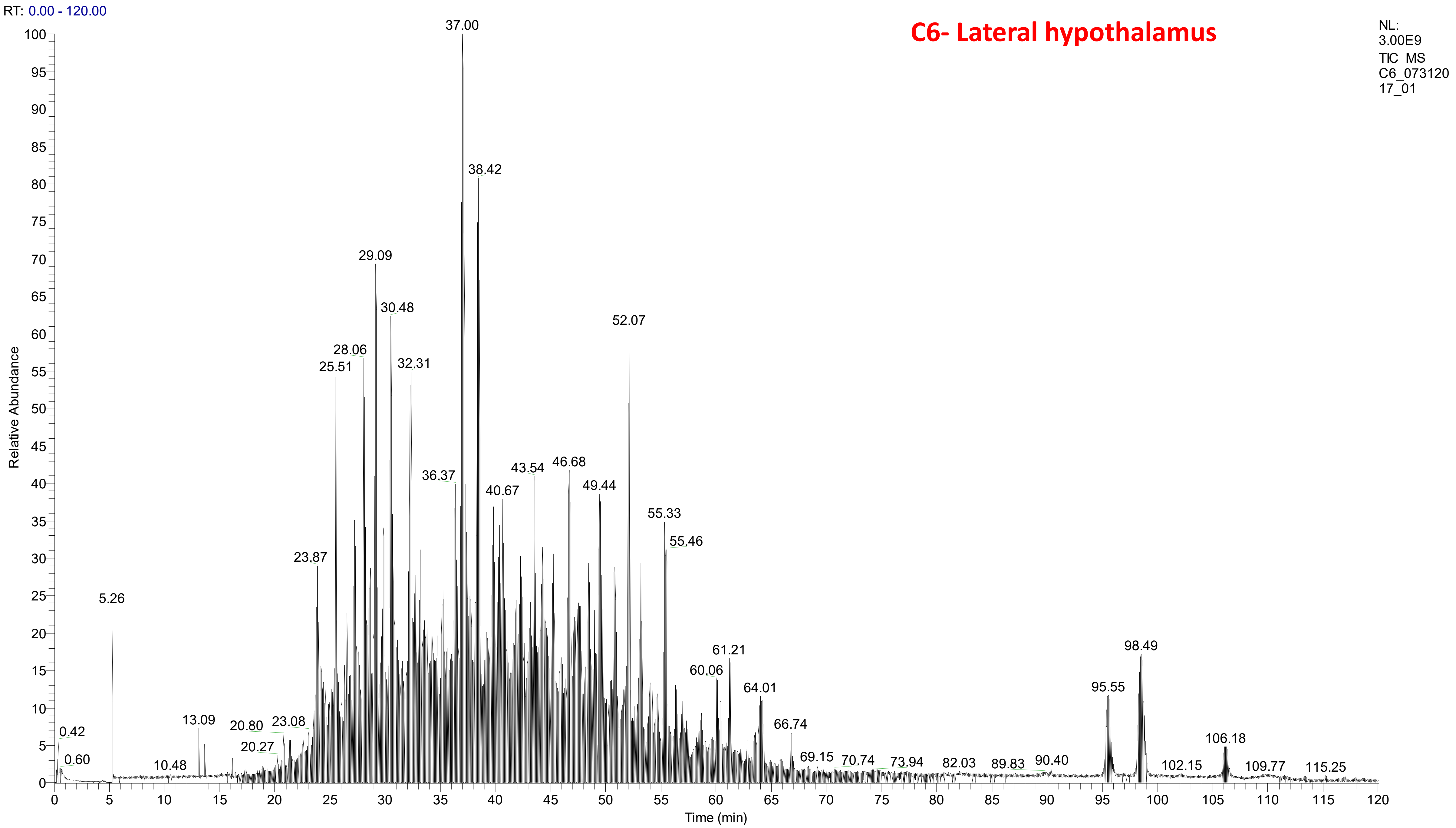
RT: 0.00 - 120.00
C6- Lateral hypothalamus
NL:
3.00E9
TIC MS
C6_073120
17_01
Relative Abundance
Time (min)
37.00
38.42
29.09
30.48
52.07
28.06
25.51
32.31
36.37
43.54
46.68
49.44
40.67
55.33
55.46
23.87
5.26
61.21
60.06
98.49
64.01
95.55
66.74
0.42
13.09
20.80
23.08
20.27
0.60
10.48
69.15
70.74
73.94
82.03
89.83
90.40
102.15
106.18
109.77
115.25

M1- Lateral hypothalamus

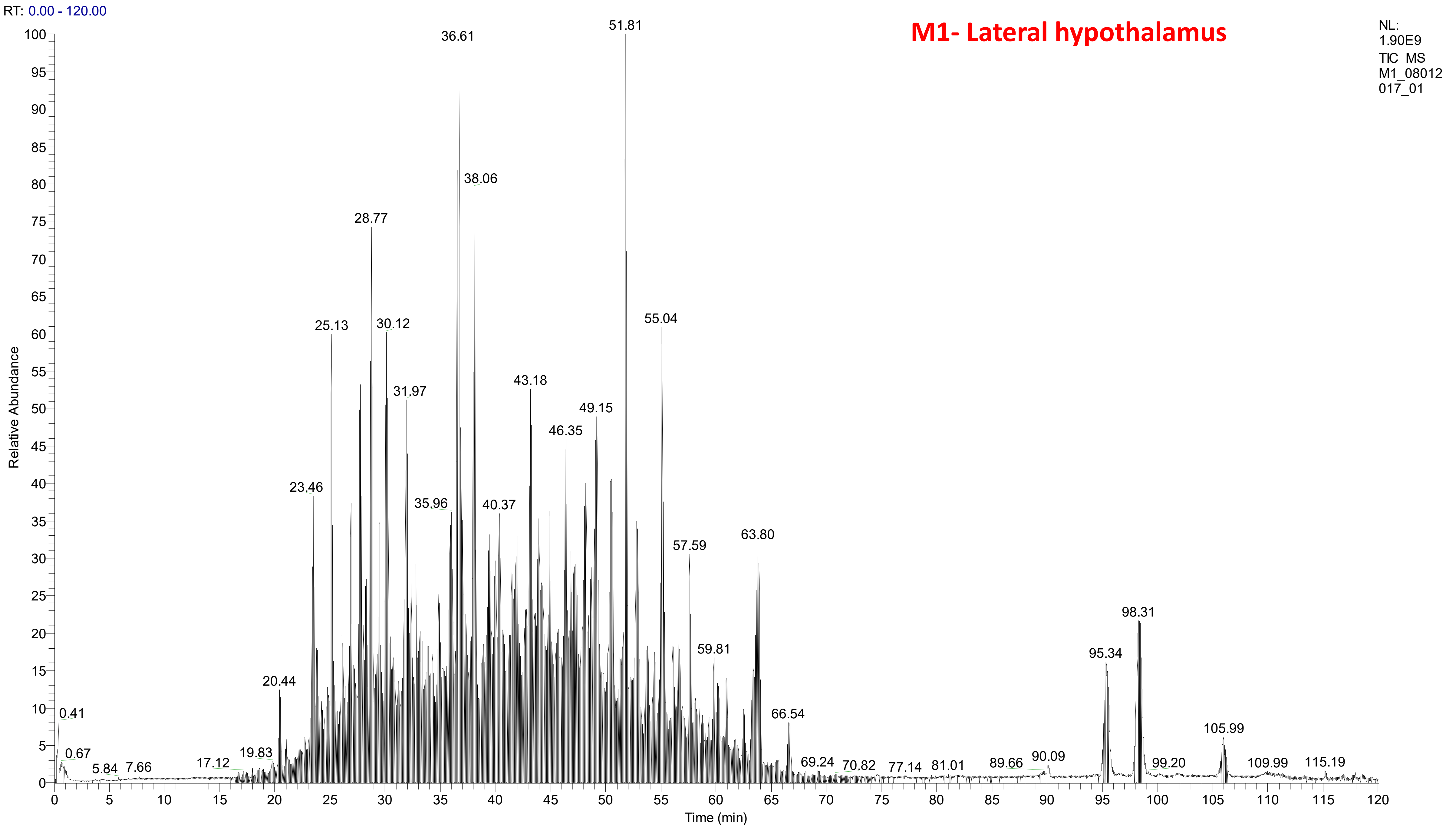

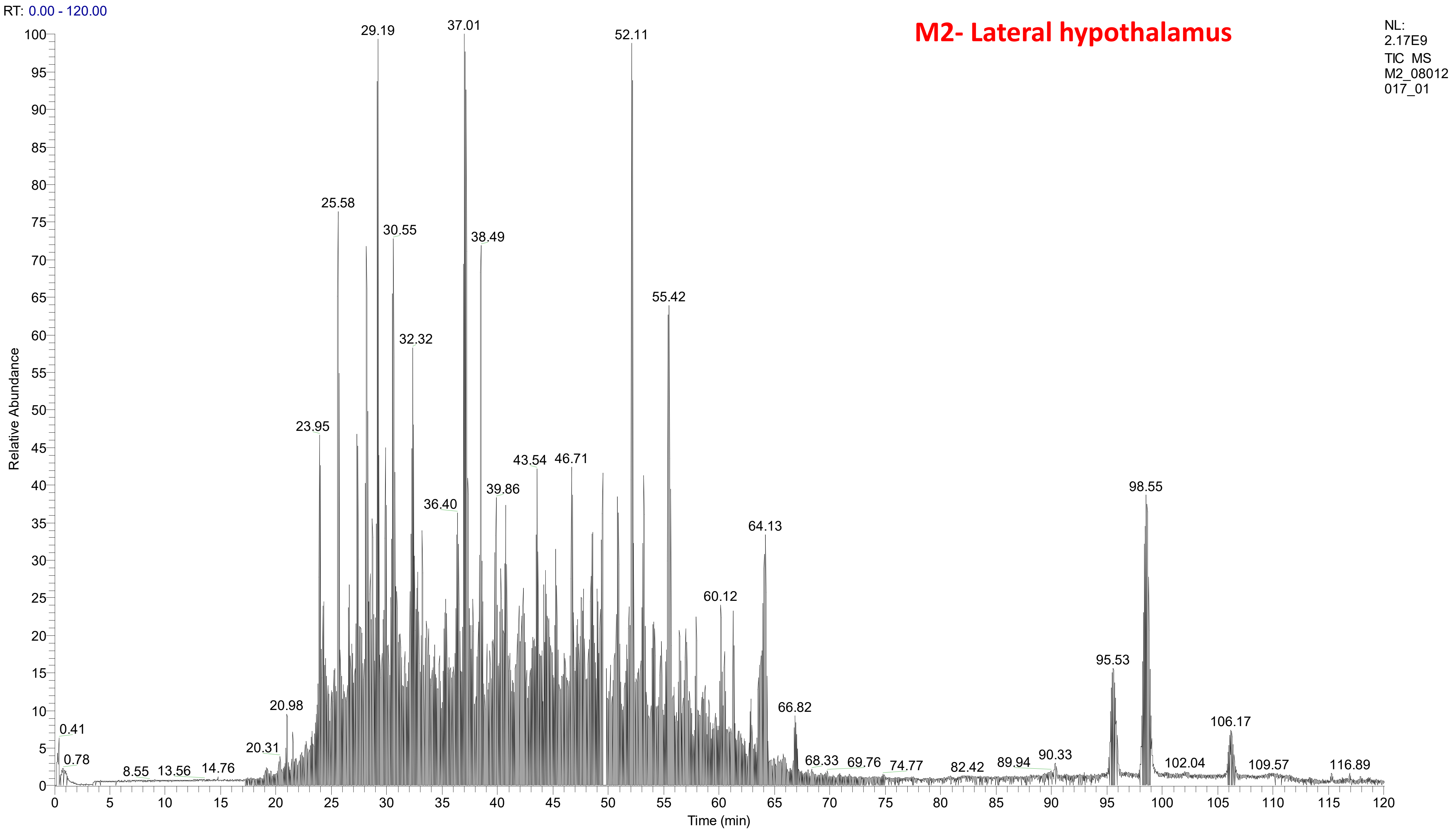
RT: 0.00 - 120.00
M2- Lateral hypothalamus
NL:
2.17E9
TIC MS
M2_08012
017_01
Relative Abundance
Time (min)
29.19
37.01
52.11
25.58
30.55
38.49
55.42
32.32
23.95
43.54
46.71
36.40
39.86
98.55
64.13
60.12
95.53
20.98
66.82
106.17
0.41
0.78
8.55
13.56
14.76
20.31
68.33
69.76
74.77
82.42
89.94
90.33
102.04
109.57
116.89

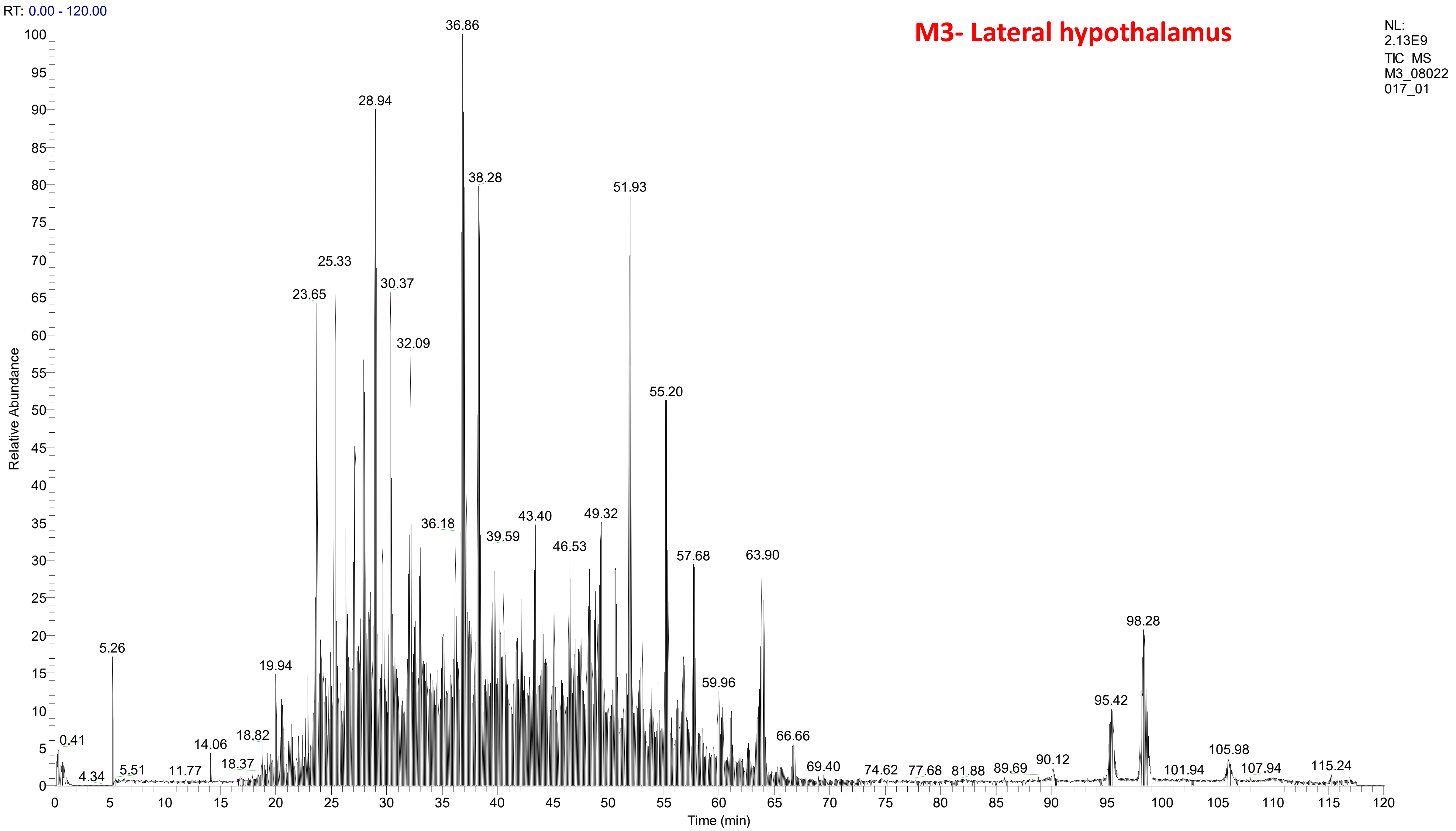
RT: 0.00 - 120.00
M3- Lateral hypothalamus
NL: 2.13E9 TIC MS M3_08022017_01
Relative Abundance
Time (min)
36.86
28.94
38.28
51.93
25.33
30.37
23.65
32.09
55.20
36.18
43.40
49.32
39.59
46.53
57.68
63.90
98.28
5.26
19.94
59.96
95.42
0.41
14.06
18.82
66.66
105.98
4.34
5.51
11.77
18.37
69.40
74.62
77.68
81.88
89.69
90.12
101.94
107.94
115.24

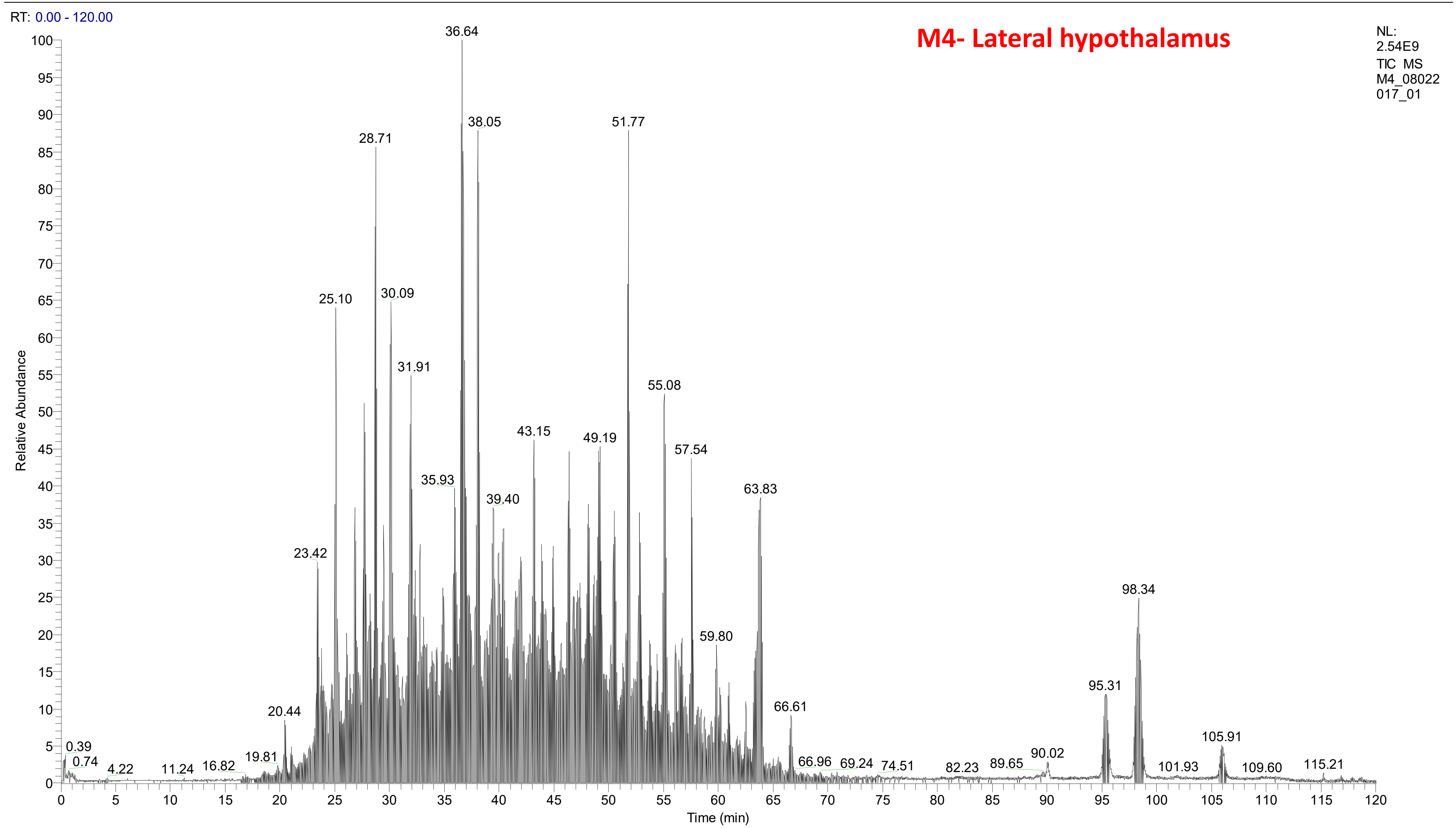

RT: 0.00 - 120.00
M4- Lateral hypothalamus
NL:
2.54E9
TIC MS
M4_08022
017_01
36.64
38.05
51.77
28.71
25.10
30.09
31.91
55.08
43.15
49.19
57.54
35.93
39.40
63.83
23.42
98.34
59.80
95.31
66.61
20.44
105.91
0.39
0.74
4.22
11.24
16.82
19.81
66.96
69.24
74.51
82.23
89.65
90.02
101.93
109.60
115.21
Relative Abundance
Time (min)

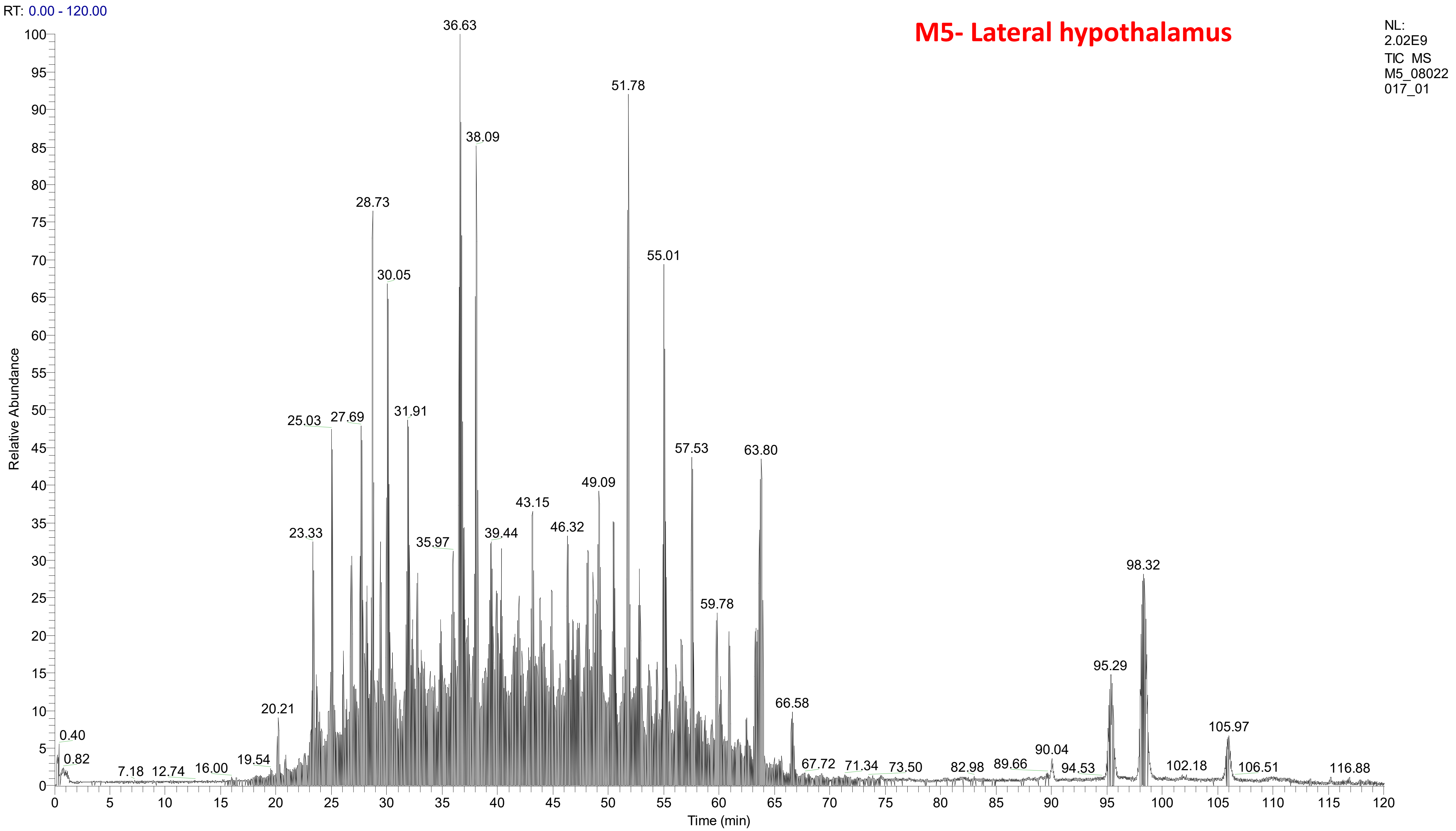
RT: 0.00 - 120.00
M5- Lateral hypothalamus
NL: 2.02E9
TIC MS
M5_08022017_01
Relative Abundance
Time (min)
36.63
51.78
38.09
28.73
55.01
30.05
31.91
27.69
25.03
57.53
63.80
49.09
43.15
46.32
23.33
39.44
35.97
98.32
59.78
95.29
66.58
105.97
20.21
0.40
0.82
7.18
12.74
16.00
19.54
67.72
71.34
73.50
82.98
89.66
90.04
94.53
102.18
106.51
116.88

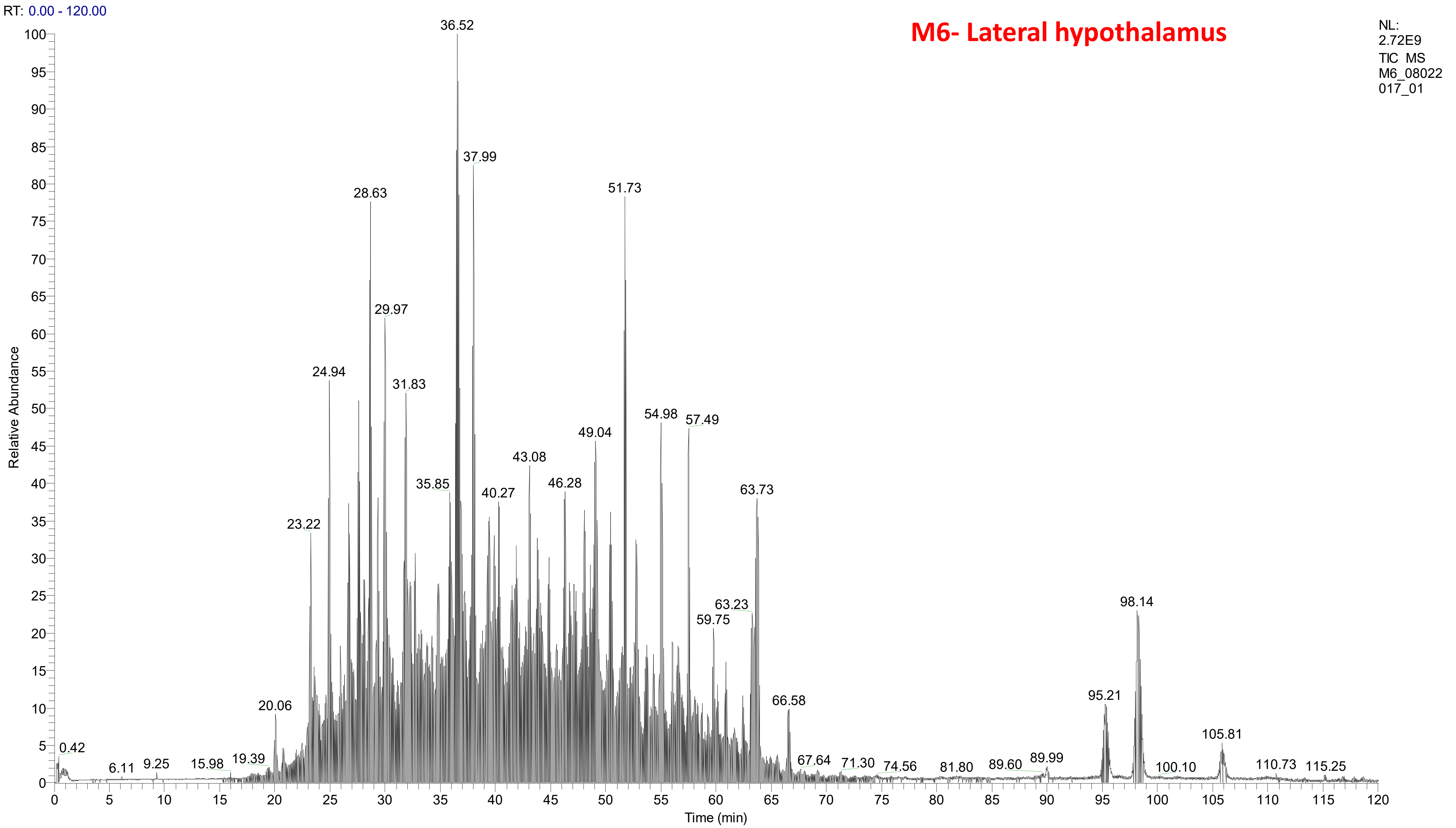

RT: 0.00 - 120.00
M6- Lateral hypothalamus
NL: 2.72E9
TIC MS
M6_08022017_01
Relative Abundance
Time (min)
36.52
37.99
28.63
51.73
29.97
24.94
31.83
54.98
57.49
49.04
43.08
35.85
46.28
40.27
63.73
23.22
63.23
59.75
98.14
20.06
66.58
95.21
105.81
0.42
6.11
9.25
15.98
19.39
67.64
71.30
74.56
81.80
89.60
89.99
100.10
110.73
115.25

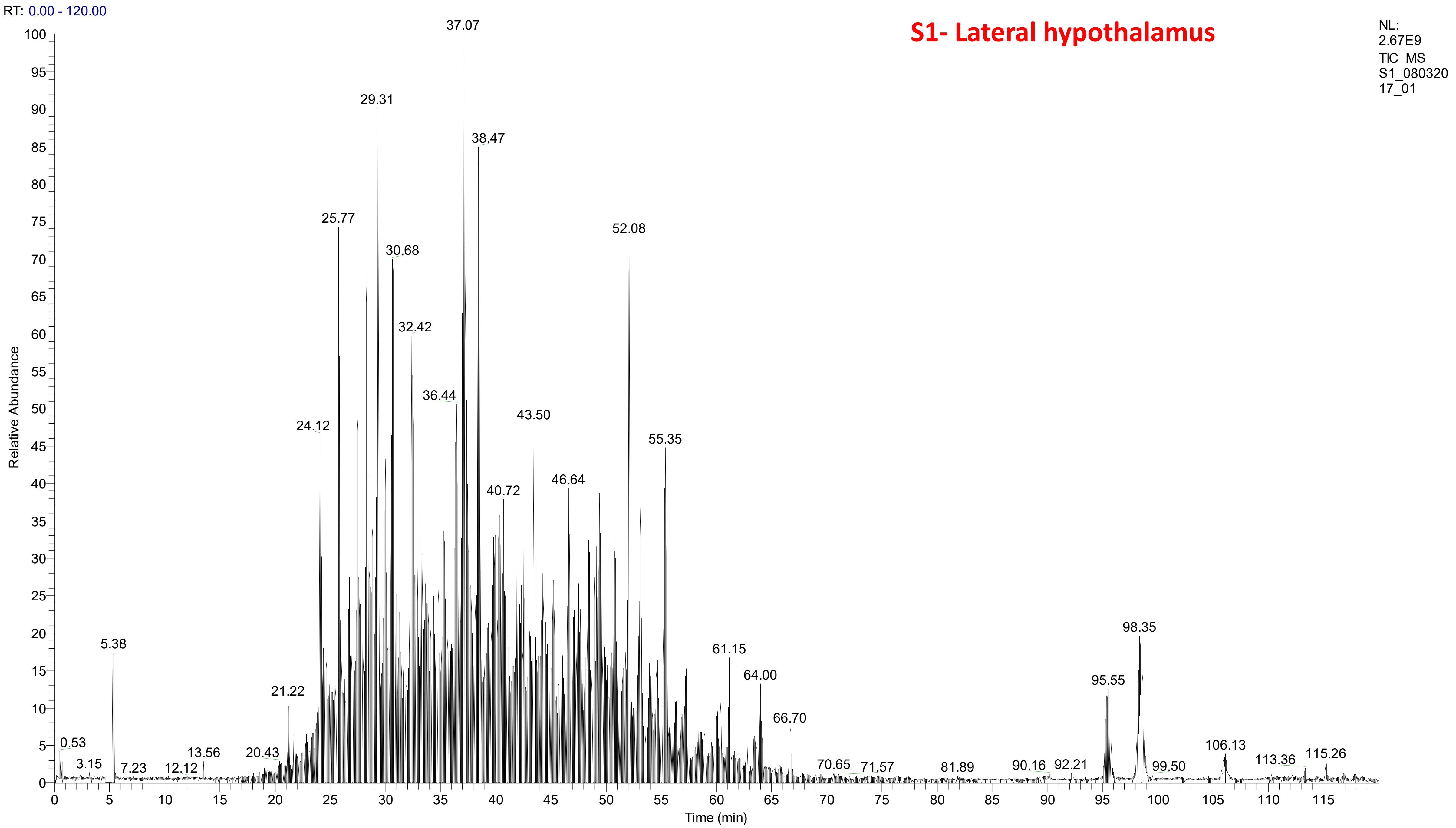

RT: 0.00 - 120.00
S1- Lateral hypothalamus
NL:
2.67E9
TIC MS
S1_080320
17_01
37.07
29.31
38.47
25.77
30.68
52.08
32.42
36.44
43.50
24.12
55.35
46.64
40.72
21.22
5.38
0.53
3.15
7.23
12.12
13.56
20.43
61.15
64.00
66.70
70.65
71.57
81.89
90.16
92.21
95.55
98.35
99.50
106.13
113.36
115.26
Relative Abundance
Time (min)

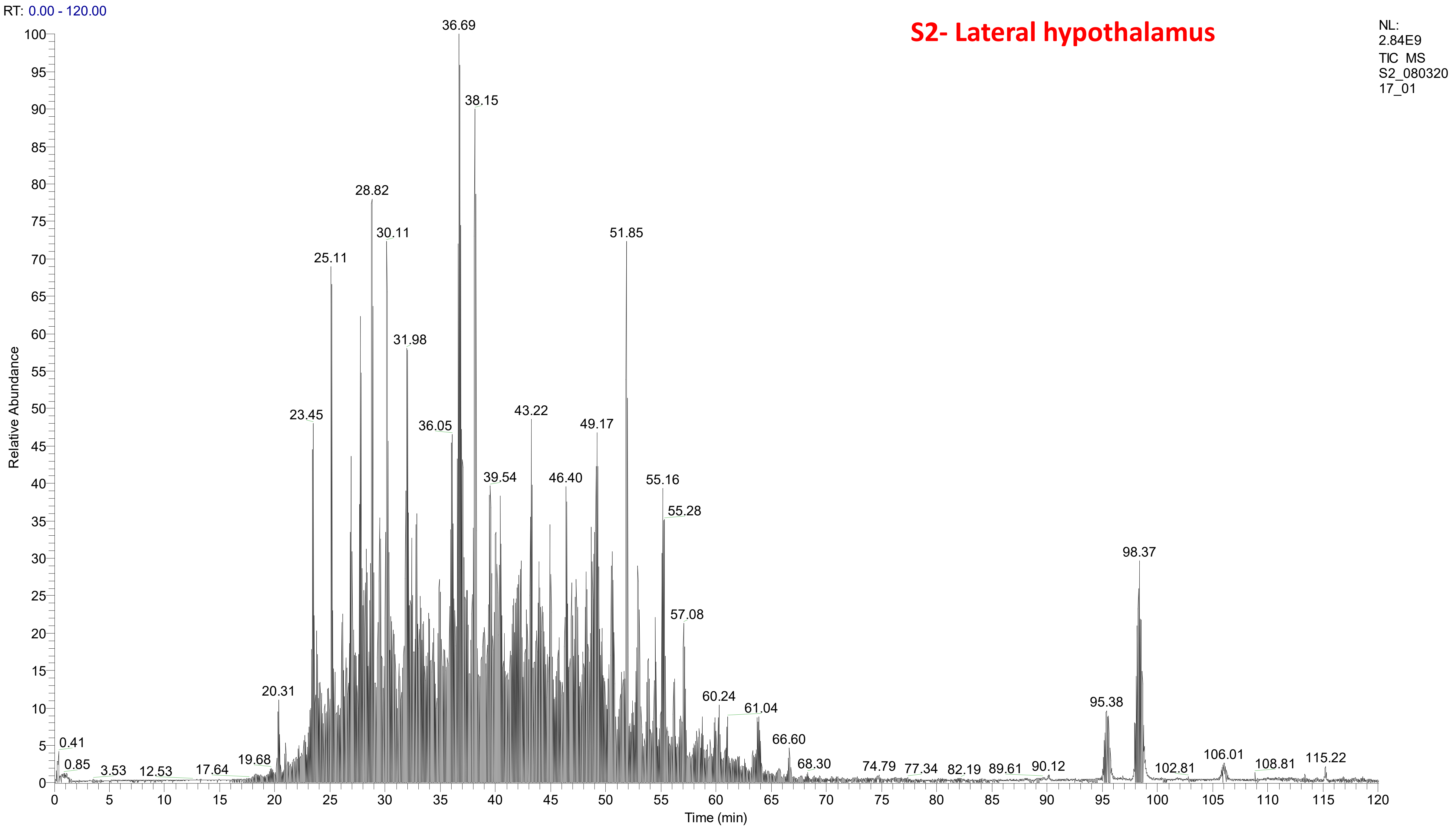
RT: 0.00 - 120.00
S2- Lateral hypothalamus
NL:
2.84E9
TIC MS
S2_080320
17_01
Relative Abundance
Time (min)
36.69
38.15
28.82
30.11
51.85
25.11
31.98
23.45
36.05
43.22
49.17
39.54
46.40
55.16
55.28
98.37
57.08
20.31
60.24
61.04
95.38
66.60
0.41
0.85
3.53
12.53
17.64
19.68
68.30
74.79
77.34
82.19
89.61
90.12
102.81
106.01
108.81
115.22

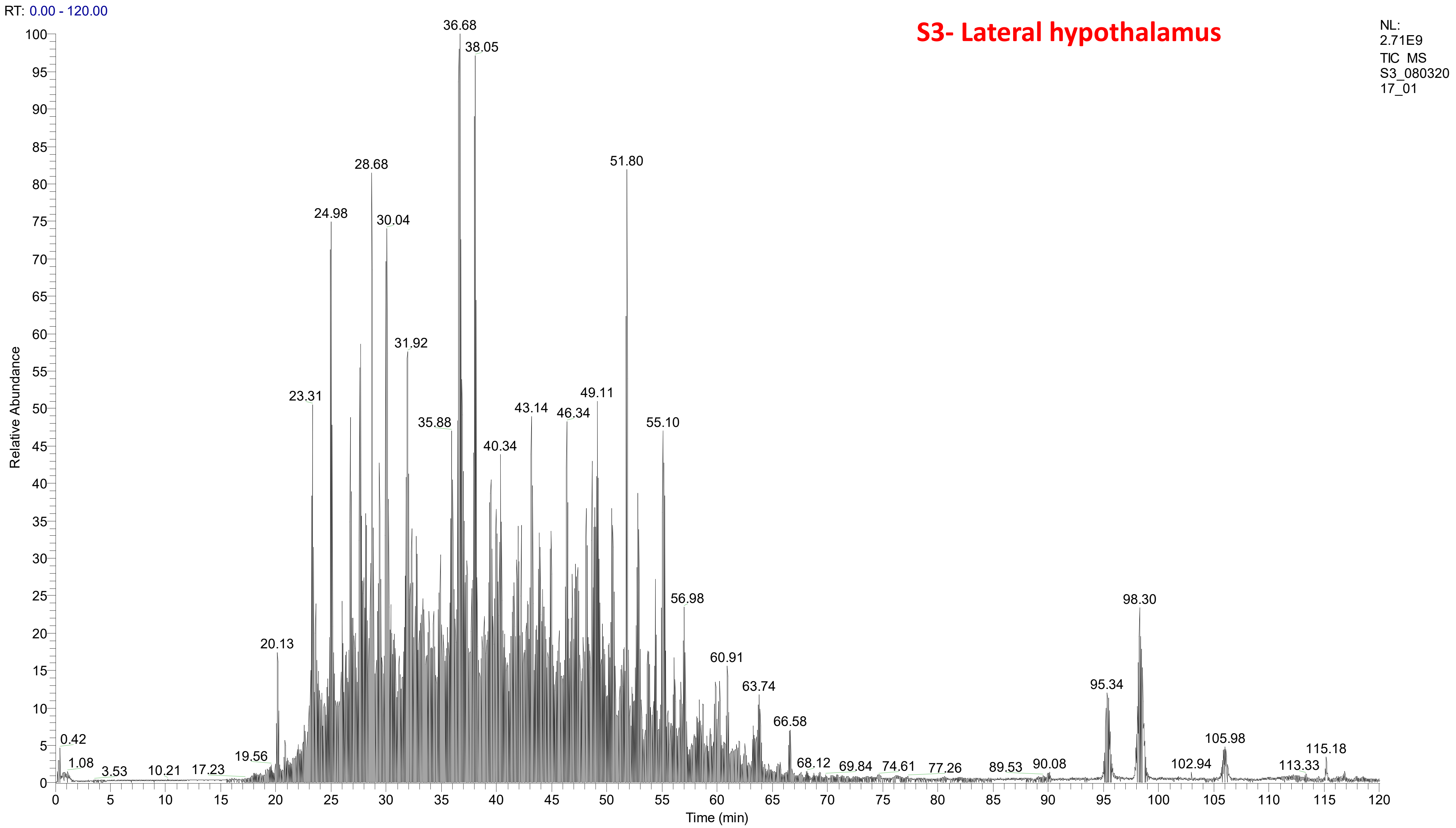
S3- Lateral hypothalamus
RT: 0.00 - 120.00
NL: 2.71E9
TIC MS
S3_080320
17_01
Relative Abundance
Time (min)
36.68
38.05
28.68
51.80
24.98
30.04
31.92
23.31
35.88
43.14
46.34
49.11
55.10
40.34
56.98
98.30
20.13
60.91
63.74
95.34
66.58
0.42
1.08
3.53
10.21
17.23
19.56
68.12
69.84
74.61
77.26
89.53
90.08
102.94
105.98
113.33
115.18

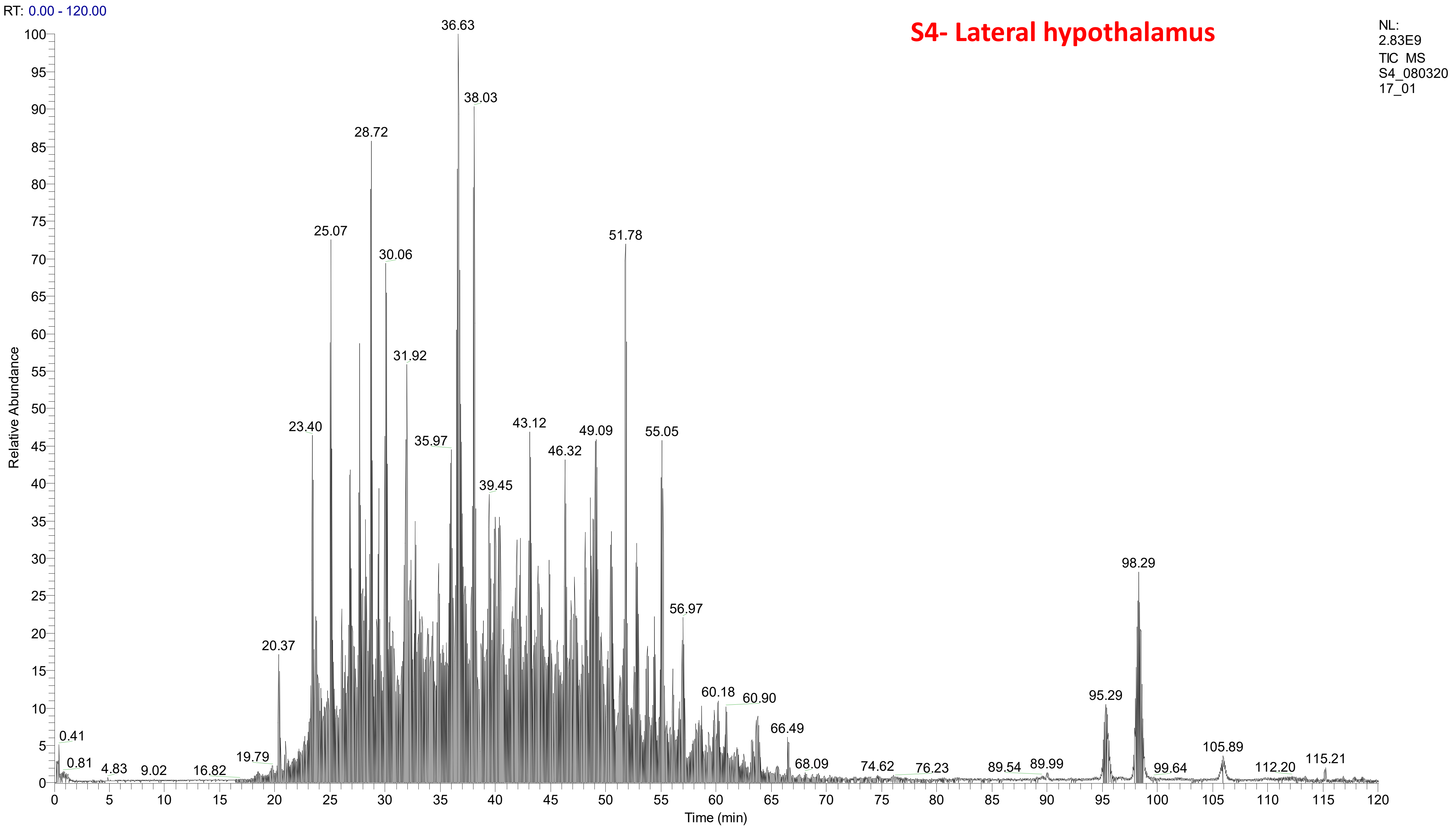

RT: 0.00 - 120.00
S4- Lateral hypothalamus
NL:
2.83E9
TIC MS
S4_080320
17_01
Relative Abundance
Time (min)
36.63
38.03
28.72
25.07
30.06
51.78
31.92
23.40
35.97
43.12
49.09
55.05
46.32
39.45
98.29
56.97
20.37
60.18
60.90
95.29
66.49
0.41
0.81
4.83
9.02
16.82
19.79
68.09
74.62
76.23
89.54
89.99
99.64
105.89
112.20
115.21

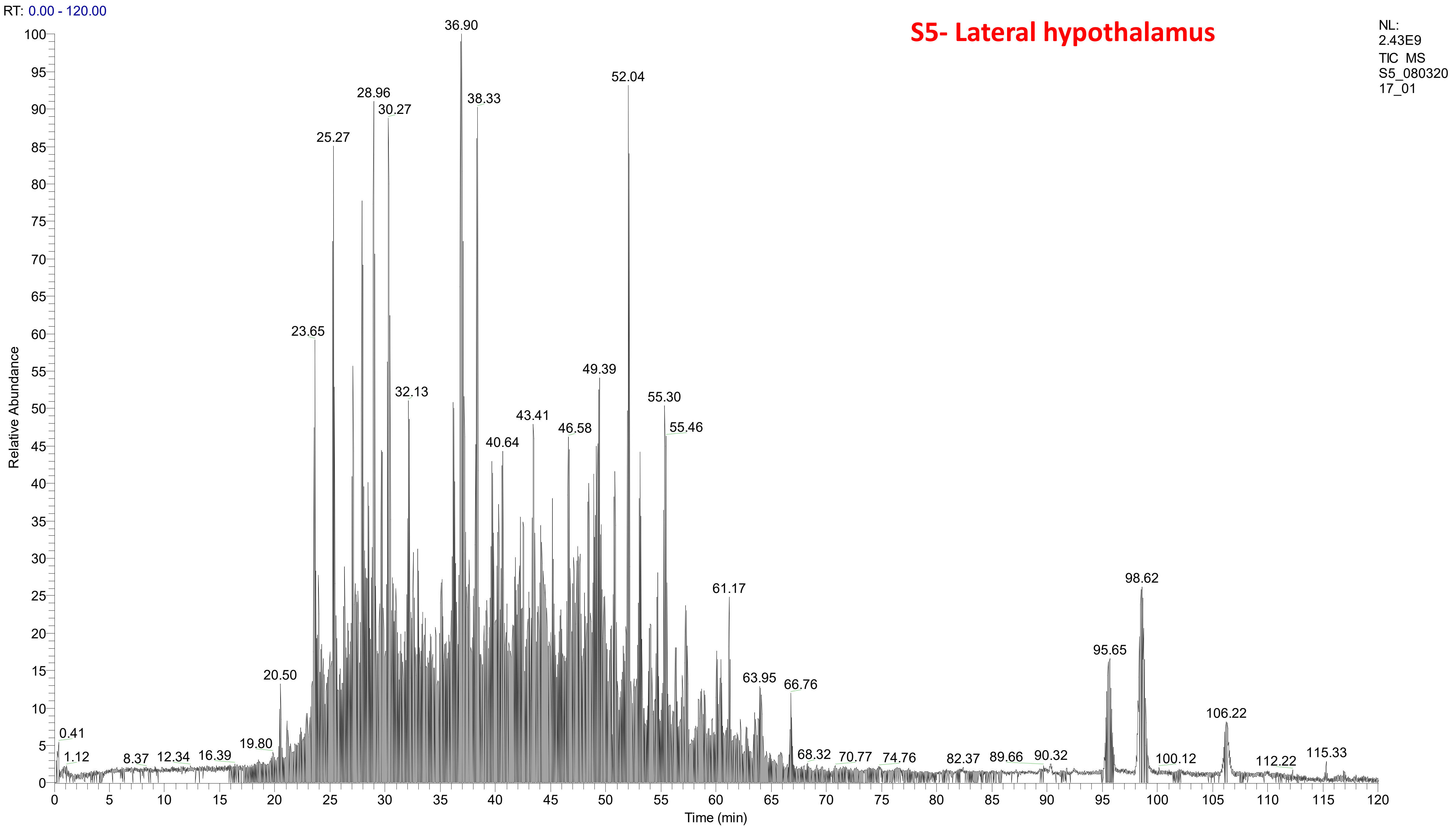
RT: 0.00 - 120.00
S5- Lateral hypothalamus
NL:
2.43E9
TIC MS
S5_080320
17_01
36.90
52.04
38.33
28.96
30.27
25.27
23.65
49.39
55.30
32.13
43.41
46.58
55.46
40.64
61.17
98.62
95.65
63.95
66.76
20.50
106.22
0.41
1.12
8.37
12.34
16.39
19.80
68.32
70.77
74.76
82.37
89.66
90.32
100.12
112.22
115.33
Relative Abundance
Time (min)

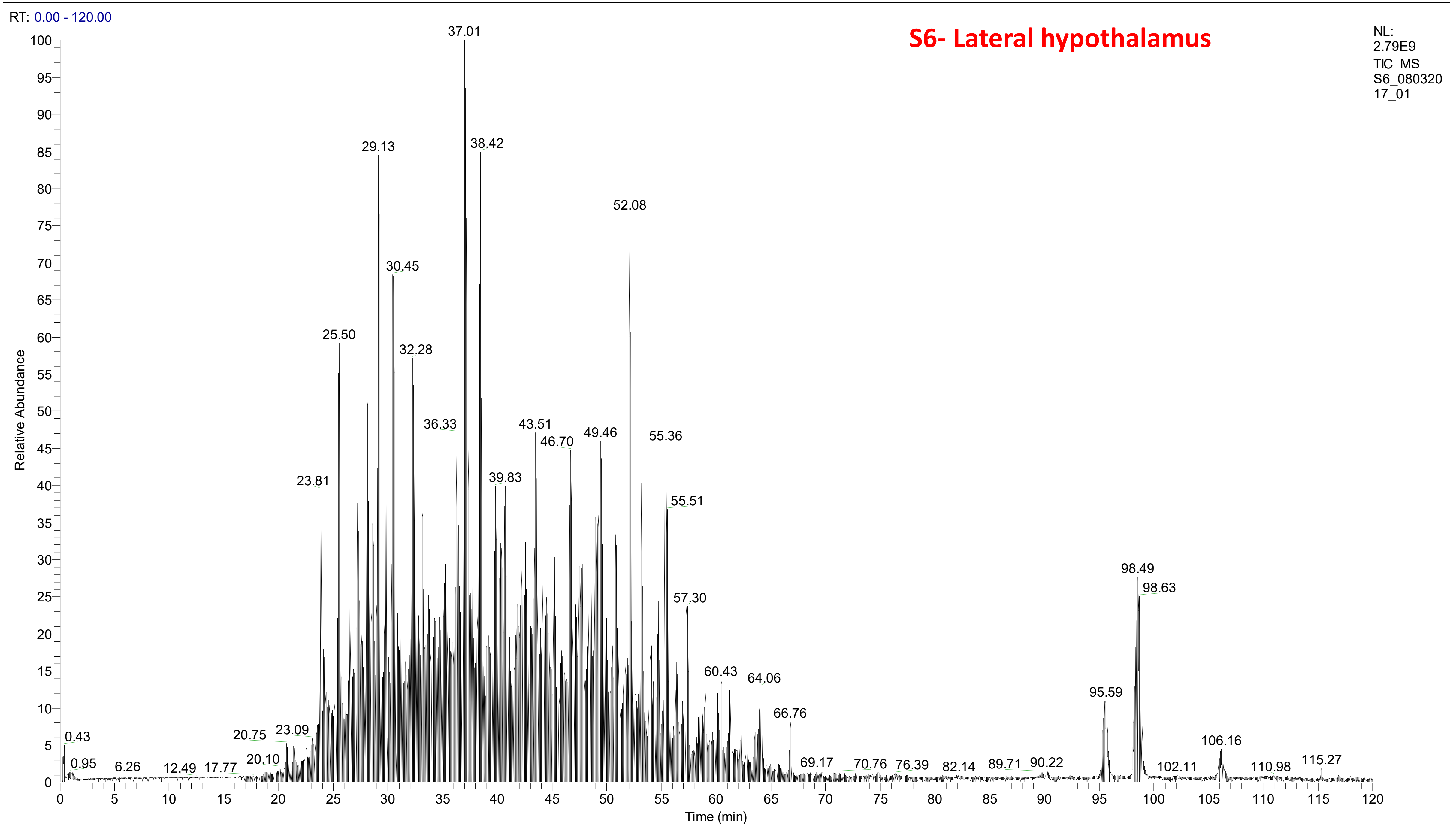
RT: 0.00 - 120.00
S6- Lateral hypothalamus
NL:
2.79E9
TIC MS
S6_080320
17_01
Relative Abundance
Time (min)
37.01
29.13
38.42
52.08
30.45
25.50
32.28
36.33
43.51
46.70
49.46
55.36
23.81
39.83
55.51
57.30
60.43
64.06
66.76
98.49
98.63
95.59
106.16
0.43
0.95
6.26
12.49
17.77
20.75
20.10
23.09
69.17
70.76
76.39
82.14
89.71
90.22
102.11
110.98
115.27
