# Supplementary material for: Comprehensive Glycomic and Proteomic Analysis of Mouse Striatum and Lateral Hypothalamus Following Repeated Exposures to Cocaine or Methamphetamine
Source: Mol Cell Proteomics. 2024 Jun 15;23(8):100803. doi: 10.1016/j.mcpro.2024.100803 (PMC11324981; doi:10.1016/j.mcpro.2024.100803)
Supplement: Supplemental PDF S2 [file mmc12.pdf]

43 PROTEINs - 2019-05-09 12:09 PM

Top Canonical Pathways

Name	p-value	Overlap	
Pentose Phosphate Pathway (Non-oxidative Branch)	7.72E-05	28.6 %	2/7
Signaling by Rho Family GTPases	1.42E-04	1.9 %	5/259
Iron homeostasis signaling pathway	1.45E-04	2.9 %	4/137
Phagosome Maturation	1.95E-04	2.7 %	4/148
Pentose Phosphate Pathway	2.01E-04	18.2 %	2/11

Top Upstream Regulators

Name	p-value	Predicted Activation
MAPT	2.81E-09	
MKNK1	4.58E-08	
APP	1.10E-07	
BDNF	1.61E-07	
PSEN1	8.83E-07	

Top Diseases and Bio Functions

Diseases and Disorders

Name	p-value range	# Molecules
Neurological Disease	2.19E-02 - 8.53E-07	24
Organismal Injury and Abnormalities	2.31E-02 - 8.53E-07	42
Developmental Disorder	2.12E-02 - 3.20E-05	9
Hereditary Disorder	2.12E-02 - 3.20E-05	20
Infectious Diseases	1.93E-02 - 1.19E-04	12

Molecular and Cellular Functions

Name	p-value range	# Molecules
Cellular Assembly and Organization	2.12E-02 - 6.47E-08	21
Cellular Function and Maintenance	2.18E-02 - 6.47E-08	27
Molecular Transport	2.12E-02 - 2.40E-07	22
Cellular Movement	2.23E-02 - 7.21E-07	16
Cell-To-Cell Signaling and Interaction	2.12E-02 - 1.54E-06	18

Physiological System Development and Function

Name	p-value range	# Molecules
Nervous System Development and Function	2.12E-02 - 3.76E-07	21
Tissue Morphology	1.75E-02 - 3.76E-07	17
Embryonic Development	2.12E-02 - 3.69E-05	12
Tissue Development	2.12E-02 - 3.69E-05	19
Organismal Development	2.10E-02 - 1.61E-04	17

Top Tox Functions

Assays: Clinical Chemistry and Hematology

Name	p-value range	# Molecules
Increased Levels of Creatinine	2.69E-02 - 2.69E-02	1

Cardiotoxicity

Name	p-value range	# Molecules
Congenital Heart Anomaly	1.64E-01 - 1.95E-03	2
Cardiac Necrosis/Cell Death	6.96E-02 - 6.96E-02	1
Cardiac Arteriopathy	1.56E-01 - 1.56E-01	2
Cardiac Proliferation	1.74E-01 - 1.74E-01	1
Cardiac Enlargement	4.81E-01 - 1.75E-01	3

Hepatotoxicity

Name	p-value range	# Molecules
Hepatocellular carcinoma	1.92E-01 - 2.62E-03	8
Liver Hyperplasia/Hyperproliferation	3.06E-01 - 2.62E-03	17
Liver Necrosis/Cell Death	9.69E-03 - 9.69E-03	1
Liver Fibrosis	2.84E-01 - 2.84E-01	1
Liver Steatosis	5.55E-01 - 5.55E-01	1

Nephrotoxicity

Name	p-value range	# Molecules
Renal Necrosis/Cell Death	4.50E-01 - 1.56E-02	2
Glomerular Injury	1.10E-01 - 2.12E-02	2
Renal Damage	2.12E-02 - 2.12E-02	1
Kidney Failure	1.10E-01 - 1.10E-01	1
Renal Fibrosis	1.10E-01 - 1.10E-01	1

Top Networks

ID	Associated Network Functions	Score
1	Cellular Assembly and Organization, Cellular Function and Maintenance, Cellular Movement	47
2	Carbohydrate Metabolism, Nucleic Acid Metabolism, Small Molecule Biochemistry	37
3	Cell-To-Cell Signaling and Interaction, Nervous System Development and Function, Hereditary Disorder	16

Top Tox Lists

Name	p-value	Overlap	
Mitochondrial Dysfunction	4.55E-03	1.7 %	3/172
Cell Cycle: G2/M DNA Damage Checkpoint Regulation	4.61E-03	3.8 %	2/52
Glutathione Depletion - CYP Induction and Reactive Metabolites	2.31E-02	8.3 %	1/12
Decreases Respiration of Mitochondria	2.69E-02	7.1 %	1/14
Decreases Depolarization of Mitochondria and Mitochondrial Membrane	6.05E-02	3.1 %	1/32

© 2000-2019 QIAGEN. All rights reserved.
