# Supplementary material for: Comprehensive Glycomic and Proteomic Analysis of Mouse Striatum and Lateral Hypothalamus Following Repeated Exposures to Cocaine or Methamphetamine
Source: Mol Cell Proteomics. 2024 Jun 15;23(8):100803. doi: 10.1016/j.mcpro.2024.100803 (PMC11324981; doi:10.1016/j.mcpro.2024.100803)
Supplement: Supplemental tables and figures [file mmc13.docx]

**Supplemental Tables and Figures**

**Supplemental Table S1.** Chondroitin sulfate (CS) and heparan sulfate (HS) disaccharide Lawrence and traditional nomenclature, Lawrence code designation, chemical structure and mass to charge ratio (m/z). References – [4-8]

| **Type of GAG** | **Disaccharides** | | | | |
| --- | --- | --- | --- | --- | --- |
| Chondroitin Sulfate (CS) | Lawrence code | Lawrence disaccharide code designation taken from [7] | Traditional  names | Chemical Structure | m/z (z=1) |
|  | D0a0 | 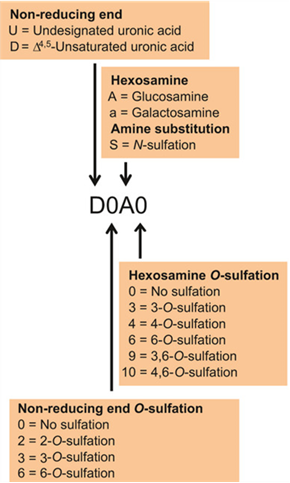 | ΔHexA-GalNAc |  | 378 |
|  | D0a4 |  | ΔHexA-GalNAc4S | 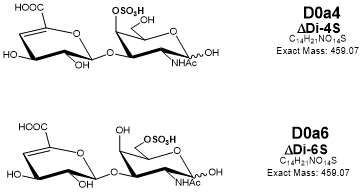 | 458 |
|  | D0a6 |  | ΔHexA-GalNAc6S | 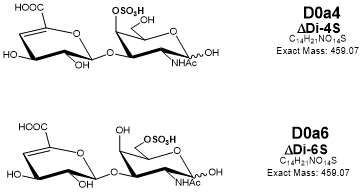 | 458 |
|  | D2a4 |  | ΔHexA2S-GalNAc4S | 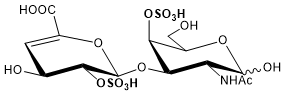 | 538 |
|  | D2a6 |  | ΔHexA2S-GalNAc6S | 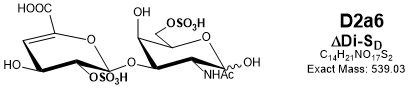 | 538 |
|  | D0a10 |  | ΔHexA-GalNAc4S6S | 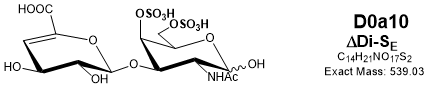 | 538 |
|  | D2a10 |  | ΔHexA2S-GalNAc4S6S | 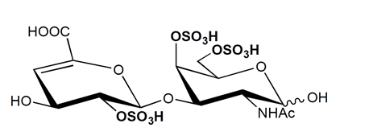 | 617 |
| Heparin Sulfate (HS) | D0A0 |  | ΔHexA-GlcNAc  (IVA ) | 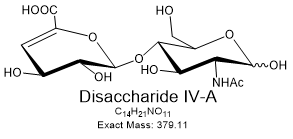 | 378 |
|  | D0A6 |  | ΔHexA-GlcNAc(6S)  (IIA) | 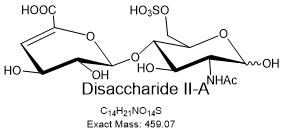 | 458 |
|  | D2A0 |  | ΔHexA(2S)-GlcNAc  (III-A) | 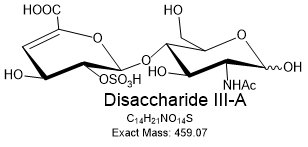 | 458 |
|  | D2A6 |  | ΔHexA(2S)-GlcNAc(6S)  (IA) | 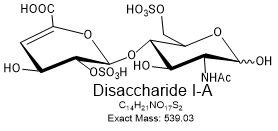 | 538 |
|  | D0S0 |  | ΔHexA-GlcNS  (IVS) | 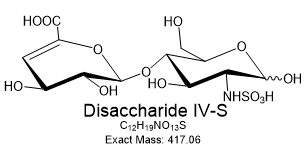 | 416 |
|  | D0S6 |  | ΔHexA-GlcNS(6S)  (IIS) | 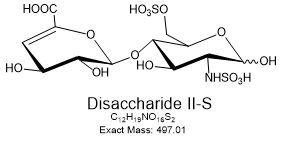 | 496 (247.5; z=2) |
|  | D2S0 |  | ΔHexA(2S)-GlcNS  (IIIS) | 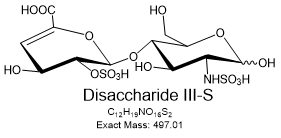 | 496 (247.5; z=2) |
|  | D2S6 |  | ΔHexA(2S)-GlcNS(6S)  (IS) | 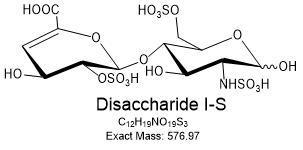 | 576  (287.5; z=2) |

**Supplemental Table S2.** A Qualitative comparison of two brain regions: lateral hypothalamus (LH), and striatum (ST) for relative abundance (%) of heparan sulfate (HS; D0A0, D2A0, D0A6, D2A6, D0S0, D2S0, D0S6, D2S6 and chondroitin sulfate (CS; D0a0, D0a4, D0a6, D0a10) disaccharides and total sulfate content (%) with difference in LH vs. ST indicated by ↑ (higher) or ↓ (lower) or no change.

| Disaccharide | LH  (% relative abundance) | ST  (% relative abundance) | Difference in LH vs. ST |
| --- | --- | --- | --- |
| Heparan Sulfate (HS) | | |  |
| D0A0 | 20.68% | 68.12% | ↓ |
| D2A0 | 11.09% | 1.85% | ↑ |
| D0A6 | 11.51% | 3.32% | ↑ |
| D2A6 | 14.82% | 0.00% | ↑ |
| D0S0 | 19.30% | 22.25% | ↓ |
| D2S0 | 5.38% | 1.58% | ↑ |
| D0S6 | 4.8% | 1.34% | ↑ |
| D2S6 | 12.43% | 1.53% | ↑ |
| Sulfate content | 41.43% | 12.16% | ↑ |
| Chondroitin Sulfate (CS) | | |  |
| D0a0 | 32.72% | 11.46% | ↑ |
| D0a4 | 28.41% | 54.54% | ↓ |
| D0a6 | 15.55% | 29.65% | ↓ |
| D0a10 | 23.33% | 4.35% | ↑ |
| Sulfate content | 45.21% | 46.43% | No change |

**Supplmental Table S3.** A list of proteoglycans and their interacting and binding partners identified in the proteomics data for cocaine, methamphetamine (METH), and saline for lateral hypothalamus (LH) and striatum (ST) with unique peptide ≥ 2.

| **Protein** | **Gene** | **Unique peptides (≥ 2)** | | | | | |
| --- | --- | --- | --- | --- | --- | --- | --- |
|  |  | **LH** | | | **ST** | | |
|  | | **Cocaine** | **METH** | **Saline** | **Cocaine** | **METH** | **Saline** |
| Aggrecan | ACAN | 4 | 2 | - | 4 | 3 | - |
| Neurocan | NCAN | - | - | - | 17 | 20 | 14 |
| Versican  (CSPG2) | VCAN | 9 | 10 | 9 | 13 | 9 | 11 |
| Brevican | BCAN | 15 | 13 | 12 | 20 | 14 | 10 |
| Chondroitin sulfate proteoglycan-4 | CSPG4 | - | 2 | - | - | - | - |
| Chondroitin sulfate proteoglycan-5 | CSPG5 | 3 | 4 | 4 | 5 | 5 | 5 |
| Hyaluronan and proteoglycan link protein 1 | HAPLN1 | - | - | - | - | - | 3 |
| Hyaluronan and proteoglycan link protein 2 | HAPLN2 | 2 | - | 2 | - | - | - |
| Intracellular hyaluronan-binding protein 4 | HABP4 | - | 2 | - | - | - | - |
| Tenascin-R | TENR | 19 | 19 | 20 | 10 | 11 | 11 |
| Neural cell adhesion molecule 1 | NCAM1 | 32 | 31 | 29 | 13 | 10 | 15 |
| Neural cell adhesion molecule 2 | NCAM2 | 2 | 2 | 2 | - | - | - |
| Neuronal cell adhesion molecule | NRCAM | 2 | 2 | 2 | 20 | 19 | 17 |
| Neural cell adhesion molecule L1 | L1CAM | 9 | 9 | 9 | 15 | 17 | 15 |
| Cell adhesion molecule 1 | CADM1 | 3 | 3 | 4 | 11 | 15 | 11 |
| Cell adhesion molecule 2 | CADM2 | 4 | 4 | 5 | 12 | 12 | 10 |
| Cell adhesion molecule 3 | CADM3 | 9 | 8 | 10 | 3 | 4 | 3 |
| Cell adhesion molecule 4 | CADM4 | 7 | - | 6 | - | - | - |
| Intercellular adhesion molecule 5 | ICAM5 | 2 | 2 | - | - | - | 2 |
| Hepatocyte cell adhesion molecule | HECAM | 7 | 7 | 7 | - | - | - |
| Hepatoma-derived growth factor | HDGF | 2 | 4 | 3 | 10 | 10 | 5 |
| Growth factor receptor-bound protein 2 | GRB2 | 2 | - | - | 8 | 8 | 8 |
| Annexin A5 | ANXA5 | 5 | 5 | 5 | 12 | 12 | 10 |
| Annexin A6 | ANXA6 | 4 | 5 | 2 | - | - | 6 |
| Annexin A7 | ANXA7 | 2 | 3 | 2 | 2 | - | - |
| Collagen alpha-1(XII) chain | COL12A1 | - | 2 | - | - | - | - |
| Collagen alpha-1(VII) chain | COL7A1 | - | - | 3 | - | - | - |
| Laminin subunit alpha-3 | LAMA3 | - | - | - | - | 2 | - |

**A,**

**B,**

**Supplemental Figure S1,** A plot of total ion chromatogram (TIC) intensity for the sample set. **A,** For lateral-hypothalamus (LH); 18 samples (with two technical replicate of each, total 36 samples); cocaine treated (1-12), METH (13-24), and saline (25-36). **B,** For striatum (ST); 18 samples (one technical replicate of each); cocaine treated (1-6), METH (7-12), and saline (13-18).

**A,**

**B,**

**Supplemental Figure S2,** Extracted ion chromatograms (EICs) for peptide m/z 496.28 from spiked internal control (pierce retention time calibration peptide mixture) intensity for lateral-hypothalamus (LH); **A,** and striatum (ST); **B.** The LC retention time and intensity over different cocaine (C), methamphetamine (M), and saline samples were consistent.

**Supplemental Figure S3,** Extracted ion chromatograms (EICs) for internal control synthetic disaccharide standard m/z 552 spiked in the lateral-hypothalamus (LH); **A,** and striatum (ST); **B.** samples. The LC retention time and intensity over different cocaine, methamphetamine (Meth), and saline samples were consistent.


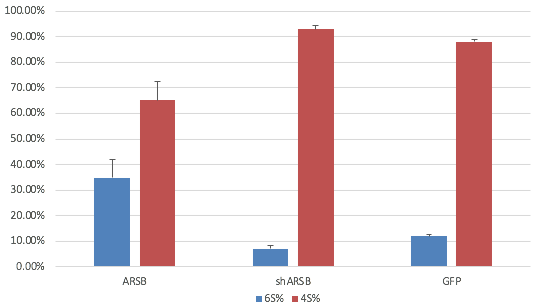


**

**

**

**

**Supplemental Figure S4,** Relative abundance of CS *6-0* (%6S) vs CS *4-0* (%4S) in the LH of mice microinjected with AAV to either overexpress Arylsulfatase B (N-acetylgalactosamine-4-sulfatase, ARSB) or to knock it down through shARSB and GFP as control using mass spectrometry CS disaccharide glycomics analysis (**= *p* ≥ 0.001).

**Supplemental Figure S5.** The overall methodology for mass spectrometry-based glycomics and proteomics using lateral hypothalamus (LH) and striatum (ST) tissue punches from mice with cocaine, methamphetamine (meth), and saline (as control) treatments.


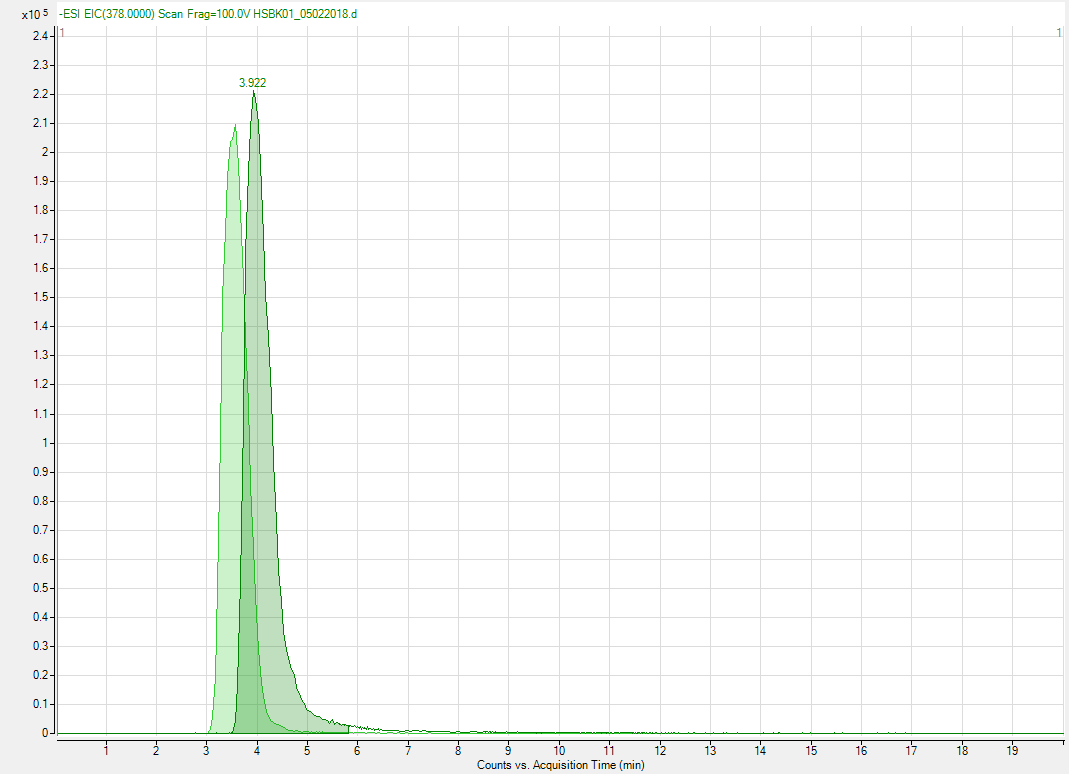


**HSBK-ST**

**HSBK-LH**

**m/z 378**

**D0A0**


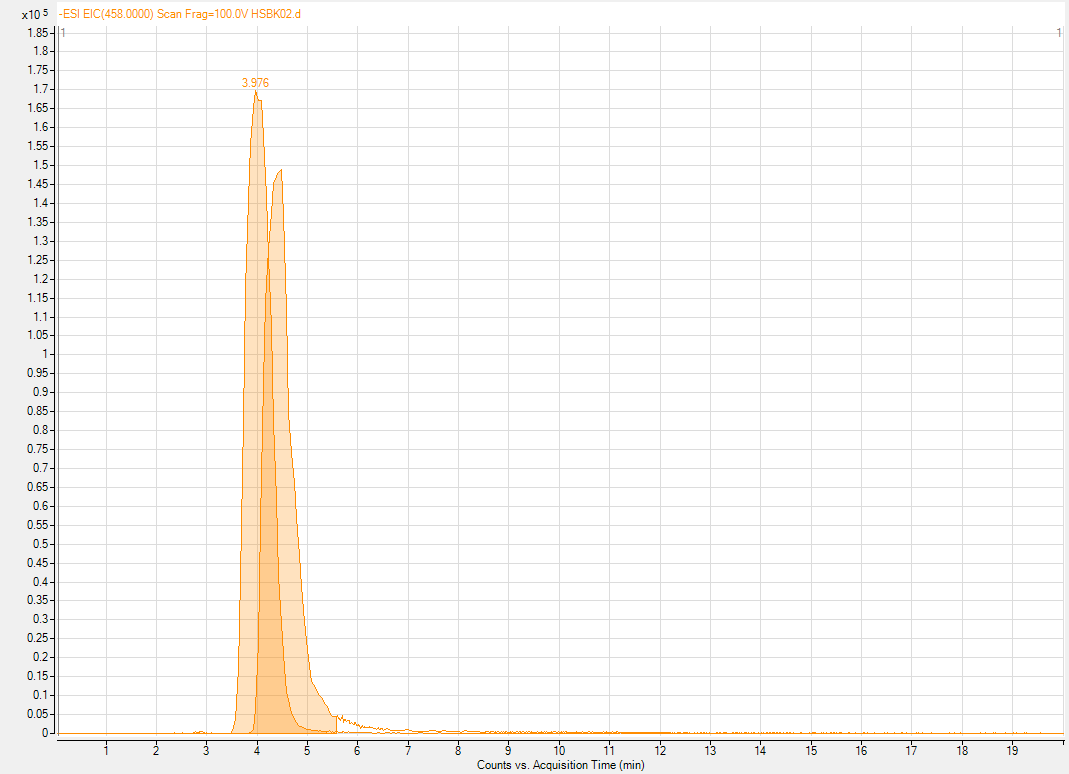


**HSBK-LH**

**HSBK-ST**

**m/z 458**

**D2A0/D0A6**

**Supplemental Figure S6.** Extracted ion chromatogram (EIC) for HS disaccharides D0A0 (above), and D2A0/D0A6 (below) for standard control sample HSBK (heparan sulfate sodium salt from bovine kidney), processed and acquired on LC-MS/MS at two separate times along with two brain regions (lateral hypothalamus (LH), and striatum (ST)) samples.

**Supplemental Figure S7.** A Venn diagram for common and exclusive proteins for saline samples for lateral hypothalamus (LH) and striatum (ST) to determine the brain region-specific proteome. A gene ontology (GO) analysis was performed using DAVID, and top biological processes (BP), cellular compartment (CC), molecular function (MF), and KEGG pathways are displayed.

**Supplemental Figure S8. A,** Venn diagram representation of common and unique proteins for cocaine (C-LH), METH (M-LH) and saline (S-LH) in lateral hypothalamus (LH). **B,** Venn diagram representation of common and unique proteins for cocaine (C-ST), METH (M-ST), and saline (S-ST) in striatum (ST). **C,** Gene ontology (GO) annotation using DAVID Bioinformatics resource 6.8 (<https://david.ncifcrf.gov/>) for **C,** cellular components, **D,** biological process, and **E,** molecular functions**, F,** KEGG pathways for cocaine (red), METH (green), saline (blue), in LH (left panel), and ST (right panel). The x-axis shows % redundant proteins i.e., number of proteins out of the total proteins per each GO term.

**Supplemental Figure S9.** An unsupervised hierarchical clustering and principal component analysis (PCA) of complete proteomics data for six biological replicates for cocaine (C1-C6), methamphetamine (M1-M6), and saline (S1-S6) for lateral hypothalamus (LH; **A, and B**), and striatum (ST; **C and D**), using R program.

**Supplemental Figure S10.** Oxidative phosphorylation as a top aberrant pathway common to both brain regions (lateral hypothalamus (LH) and striatum (ST), and both drug treatments (cocaine and METH) vs. saline obtained by KEGG pathway analysis using DAVID. Stars= differentially expressed proteins observed in this study, red; cocaine and METH vs. Saline, blue; cocaine vs. saline, and green; METH vs. saline.


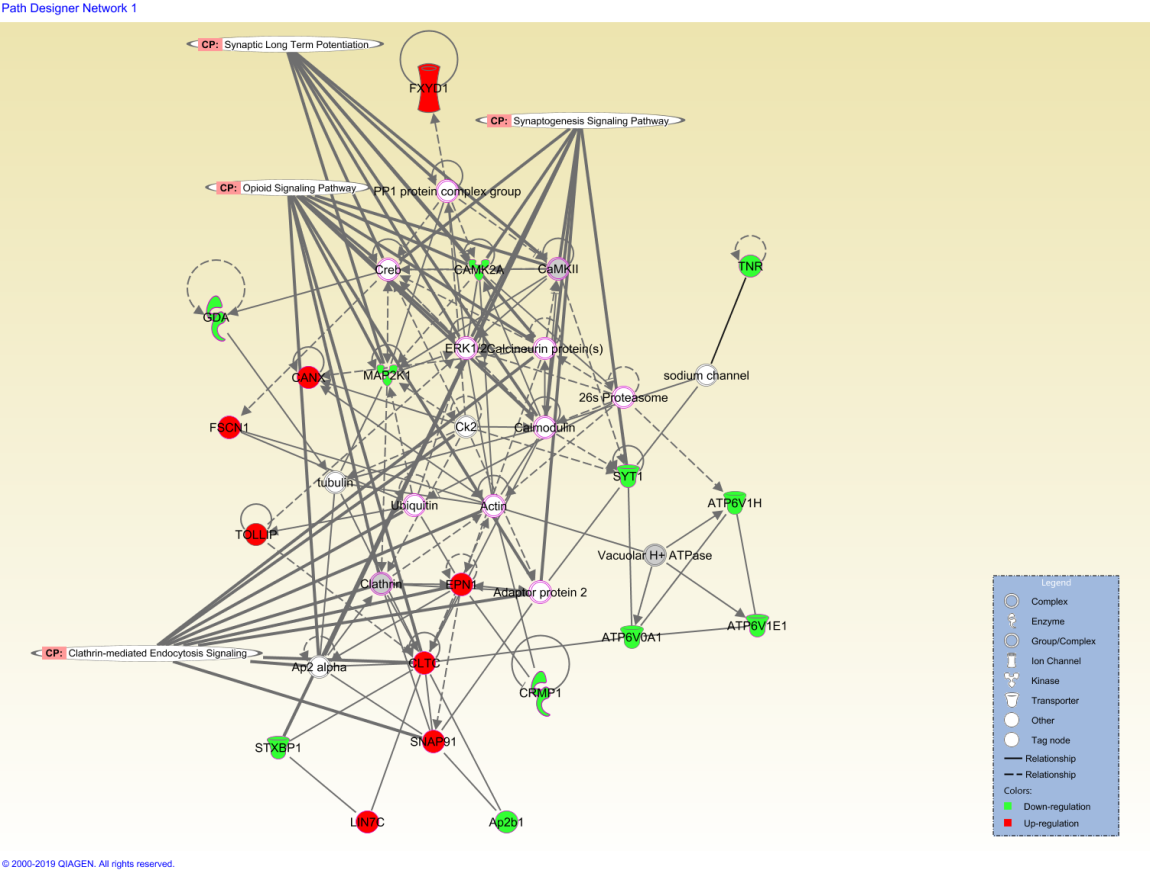


**Supplemental Figure S11.** A network analysis using Ingenuity pathway analysis (IPA) showed cellular assembly and organization, cellular function and maintenance, cellular movement as the top network. The network was overlayed with diseases & biological functions (nervous system and development function, neurological disease, developmental disorder, and psychological disorder; pink border), and canonical pathways (opioid signaling pathway, clathrin-mediated endocytosis signaling, synaptic long-term potentiation, and synaptogenesis signaling pathway). Legend is displayed on the figure.
